# Supplementary material for: High diversity of coralline algae in New Zealand revealed: Knowledge gaps and implications for future research
Source: PLoS One. 2019 Dec 2;14(12):e0225645. doi: 10.1371/journal.pone.0225645 (PMC6886753; doi:10.1371/journal.pone.0225645)
Supplement: S1 Table — Specimens are listed alphabetically within the three orders.— = no data available. (PDF) [file pone.0225645.s001.pdf]

S1 Table. List of global coralline algal type specimens sourced from GenBank for use in concatenated *psb*A and *rbc*L phylogenetic analyses. Specimens are listed alphabetically within the three orders. - = no data available

Species	Order	Reference	Type	Voucher	Location	GenBank accession number	
						*psb*A	*rbc*L
Alatocladia modesta	Corallinales	Hind & Saunders 2013	Topotype	GWS013775	Japan: Chiba-ken	JQ422193	-
Amphiroa zonata	Corallinales	Hind et al. 2018	Topotype	GWS013768	Japan: Kanto, Chiba-ken, Katsuura	MH777612	MH777616
Arthrocardia corymbosa	Corallinales	Hind et al. 2014	Topotype	NCU_594345	Cape of Good Hope, South Africa	JQ917408	JN701475
Bossiella plumosa	Corallinales	Hind et al. 2015	Identical to Holotype	UBC_A89956	Canada: British Columbia, Vancouver Island,	KT782167	KT782053
Chamberlainium tumidum	Corallinales	Van der Merwe et al. 2015	Identical to Lectotype	NCU_600394	USA: California, Monterey Co., Pacific Grove	KT184830	KT184844
Chiharaea bodegensis	Corallinales	Martone et al. 2012	Topotype	UC_1944755	USA: California, Marin Co., Bodega Head	-	HQ322332
Corallina ferreyrae	Corallinales	Bustamante et al. 2019	Isotype	UC_1404138	Peru: Pucusana, al Sur de Lima	MK408748	MK408748
Corallina officinalis	Corallinales	Hind et al. 2014	Identical to Isolectotype	NCU_588625	USA: Alaska, Kodiak Island, Deadman	KJ637650	KJ591668
Dawsoniolithon conicum	Corallinales	Caragnano et al. 2018	-	NOU_87006	French Polynesia: Tahuata, Marquesas	MG851076	-
Harveylithon rupestre	Corallinales	Rosler et al. 2016	-		Australia	KM407535	-
Hydrolithon reinboldii	Corallinales	Caragnano et al. 2018	-	NOU_87011	Maldives: Baa	MG851060	-
Johansenia macmillanii	Corallinales	Hind et al. 2014	Topotype	NCU_588188	Canada: British Columbia, Vancouver Island, Port Renfrew	KJ637657	HQ322338
Lithophyllum hibernicum	Corallinales	Hernandez-Kantun et al. 2016	Identical to Lectotype	US170957	Ireland: Clare Co., Black Head	KR708594	KR708550
Lithophyllum incrustans	Corallinales	Hernandez-Kantun et al. 2016	Identical to Holotype	GALW13587	Ireland: Cork Co., Roaringwater Bay	KR708604	KR708583
Mastophora rosea	Corallinales	Genbank - Saunders	Topotype	PAGO0003B	USA: Guam, Pago Bay	KP224287	KP224278
Pneophyllum fragile	Corallinales	Zuljevic et al. 2016	Topotype	BM001033381	Spain: Almadra	KT783426	-
Pneophyllum limitatum	Corallinales	Zuljevic et al. 2016	Identical to holotype	BM000771741	United Kingdom: Bembridge, Isle of Wight	KT783427	-
Porolithon onkodes	Corallinales	Gabrielson et al. 2018	Lectotype	TRH A26-1494	Papua New Guinea: Tami Island	KY212106	KY212106
Callilithophytum parcum	Hapalidales	Adey et al. 2015	Identical to Holotype	UC_1918246	USA: California, San Mateo Co., Moss Beach	KP142739	KP142781
Clathromorphum compactum	Hapalidales	Adey et al. 2015	Lectotype	US_170929	Canada: Labrador, south of Windy Tickle Island	KP142730	KP142774
Clathromorphum nereostratum	Hapalidales	Adey et al. 2015	Isotype	US_170931	USA: Alaska, Aleutian Islands, Alaid Island	KP142733	KP142777
Crustaphytum pacificum	Hapalidales	Liu et al. 2018	Holotype	NTOU_1359	Taiwan: Xinwu, Taoyuan County	MH376921	-
Lithothamnion corallioides	Hapalidales	Melbourne et al. 2017	-	BM001150587	United Kingdom: Falmouth	KX828386	-
Lithothamnion glaciale	Hapalidales	Adey et al. 2015	-	US_170935	Canada: Quebec, off Havre Cumberland, Center Island	KP142721	KP142766
Lithothamnion lemoineae	Hapalidales	Adey et al. 2015	Identical to Holotype	US_169116	Canada: Labrador, Hopedale Run, Crab Island	KP142723	KP142768
Lithothamnion tophiforme	Hapalidales	Adey et al. 2015	-	US_170938	Canada: Labrador, Porcupine Bay	KP142720	KP142765
Mesophyllum lichenoides	Hapalidales	Gabrielson et al. 2019	Close to type locality	NCU_590286	United Kingdom: England, South Devon	MF034552	KY994129
Neopolyporolithon arcticum	Hapalidales	Gabrielson et al. 2019	Identical to Lectotype	AM-CC-I	USA: Alaska, Amchitka Island, Aleutian Islands	KP142748	KP142784
Neopolyporolithon reclinatum	Hapalidales	Adey et al. 2015	Identical to Holotype	UBC_A88609	Canada: British Columbia, Victoria, Harling	KP142762	KP142806
Phymatolithon calcareum	Hapalidales	Adey et al. 2015	Neotype	BM00712373	United Kingdom: England, Falmouth	JQ896231	KX020487
Phymatolithon rugulosum	Hapalidales	Adey et al. 2018	Identical to Holotype	BM000659095	Germany: Helgoland	MH252261	MH274810
Heydrichia cerasina	Sporolithales	Maneveldt et al. 2017	Isotype	NCU_617165	South Africa: Cape Agulhas, Western Cape	MF034551	KY994128
Heydrichia woelkerlingii	Sporolithales	Maneveldt et al. 2017	Topotype	NCU_597127	South Africa: Cape Province, Oudekraal	JQ917415	KP142788
Sporolithon durum	Sporolithales	Nelson et al. 2015	-	LTB21140	Australia	DQ168023	KM369121
Sporolithon indopacificum	Sporolithales	Maneveldt et al. 2018	Holotype	L_3964509	Tanzania: Zanzibar, Chwaka Bay	MG051270	MG051266
Sporolithon molle	Sporolithales	Maneveldt et al. 2017	Topotype	NCU_606657	Egypt: Gulf of Suez, El Tor,	MG051272	KY994120
Sporolithon ptychoides	Sporolithales	Maneveldt et al. 2017	Topotype	NCU_606660	Egypt: Gulf of Suez, El Tor	MF034541	KY994117
Sporolithon tenue	Sporolithales	Maneveldt et al. 2017	Isotype	US_170943	Brazil: Salvador, Itapua	KP142751	KP142785
Sporolithon yoneshigueae	Sporolithales	Maneveldt et al. 2017	Isotype	TBA1	Brazil: Abrolhos	MF034545	KY994122

Adey, W. H., Hernandez-Kantun, J. J., Johnson, G., & Gabrielson, P. W. (2015). DNA sequencing, anatomy, and calcification patterns support a monophyletic, subarctic, carbonate reef-forming Clathromorphum (Hapalidiaceae, Corallinales, Rhodophyta). *Journal of Phycology*, *51*, 189–203.

Bustamante, D. E., Calderon, M. S., & Hughey, J. R. (2019). Conspecificity of the Peruvian *Corallina ferreyrae* with *C. caespitosa* (Corallinaceae, Rhodophyta) inferred from genomic analysis of the type specimen. *Mitochondrial DNA Part B*, *4*(1), 1285–1286.

Caragnano, A., Foetisch, A., Maneveldt, G. W., Millet, L., Liu, L., Lin, S., Rodondi, G., & Payri, C. E. (2018). Revision of Corallinaceae (Corallinales, Rhodophyta): recognizing *Dawsoniolithon* gen. nov., *Parvicellularium* gen. nov. and Chamberlainoideae subfam. nov. containing *Chamberlainium* gen. nov. and *Pneophyllum*. *Journal of Phycology*, *54*, 391–409.

Gabrielson, P. W., Hughey, J. R., & Diaz-Pulido, G. (2018). Genomics reveals abundant speciation in the coral reef building alga *Porolithon onkodes* (Corallinales, Rhodophyta). *Journal of Phycology*, *54*, 429–434.

Gabrielson, P. W., Lindstrom, S. C., & Hughey, J. R. (2019). *Neopolyporolithon loculosum* is a junior synonym of *N. arcticum* comb. nov.(Hapalidiales, Rhodophyta), based on sequencing type material. *Phycologia*, 1–5.

Hernandez-Kantun, J. J., Gabrielson, P., Hughey, J. R., Pezzolesi, L., Rindi, F., Robinson, N. M., … Adey, W. H. (2016). Reassessment of branched *Lithophyllum* spp.(Corallinales, Rhodophyta) in the Caribbean Sea with global implications. *Phycologia*, *55*, 619–639.

Hind, K. R., Gabrielson, P. W., Lindstrom, S. C., & Martone, P. T. (2014). Misleading morphologies and the importance of sequencing type specimens for resolving coralline taxonomy (Corallinales, Rhodophyta): *Pachyarthron cretaceum* is *Corallina officinalis*. *Journal of Phycology*, *50*, 760–764.

Hind, K. R., & Saunders, G. W. (2013). A molecular phylogenetic study of the tribe Corallineae (Corallinales, Rhodophyta) with an assessment of genus-level taxonomic features and descriptions of novel genera. *Journal of Phycology*, *49*, 103–114.

Liu, L.-C., Lin, S.-M., Caragnano, A., & Payri, C. (2018). Species diversity and molecular phylogeny of non-geniculate coralline algae (Corallinophycidae, Rhodophyta) from Taoyuan algal reefs in northern Taiwan, including *Crustaphytum* gen. nov. and three new species. *Journal of Applied Phycology*, *30*, 3455–3469.

Maneveldt, G. W., Gabrielson, P. W., & Kangwe, J. (2017). *Sporolithon indopacificum* sp. nov. (Sporolithales, Rhodophyta) from tropical western Indian and western Pacific oceans: First report, confirmed by DNA sequence data, of a widely distributed species of *Sporolithon*. *Phytotaxa*, *326*, 115–128.

Maneveldt, G. W., Puckree-Padua, C., & Gabrielson, P. W. (2018). Inspired by the joy of new discoveries—uncovering cryptic coralline algal diversity. *SANCOR Newsletter*, 4–6.

Martone, P. T., Lindstrom, S. C., Miller, K. A., & Gabrielson, P. W. (2012). *Chiharaea* and *yamadaia* (Corallinales, Rhodophyta) represent reduced and recently derived articulated coralline morphologies. *Journal of Phycology*, *48*, 859–868.

Melbourne, L. A., Hernández-Kantún, J. J., Russell, S., & Brodie, J. (2017). There is more to maerl than meets the eye: DNA barcoding reveals a new species in Britain, *Lithothamnion erinaceum* sp. nov.(Hapalidiales, Rhodophyta). *European Journal of Phycology*, *52*, 166–178.

Nelson, W. A., Sutherland, J. E., Farr, T. J., Hart, D. R., Neill, K. F., Kim, H. J., & Yoon, H. S. (2015). Multi-gene phylogenetic analyses of New Zealand coralline algae: *Corallinapetra novaezelandiae* gen. et sp. nov. and recognition of the Hapalidiales ord. nov. *Journal of Phycology*, *51*, 454–468.

Rösler, A., Perfectti, F., Peña, V., Braga, J. C., & Gabrielson, P. (2016). Phylogenetic relationships of corallinaceae (Corallinales, Rhodophyta): taxonomic implications for reef-building corallines. *Journal of Phycology*, *52*, 412–431. https://doi.org/10.1111/jpy.12404

van der Merwe, E., Miklasz, K., Channing, A., Maneveldt, G. W., & Gabrielson, P. W. (2015). DNA sequencing resolves species of *Spongites* (Corallinales, Rhodophyta) in the Northeast Pacific and South Africa, including *S. agulhensis* sp. nov. *Phycologia*, *54*, 471–490.

Žuljević, A., Kaleb, S., Peña, V., Despalatović, M., Cvitković, I., De Clerck, O., … Braga, J. C. (2016). First freshwater coralline alga and the role of local features in a major biome transition. *Scientific Reports*, *6*, 19642.
