# Supplementary material for: High diversity of coralline algae in New Zealand revealed: Knowledge gaps and implications for future research
Source: PLoS One. 2019 Dec 2;14(12):e0225645. doi: 10.1371/journal.pone.0225645 (PMC6886753; doi:10.1371/journal.pone.0225645)
Supplement: S2 Table — (PDF) [file pone.0225645.s002.pdf]

S2 Table. Partition schemes and sequence evolution models used for combined *psb*A and *rbc*L datasets in implementing (A) maximum likelihood phylogenetic analysis and (B) Bayesian phylogenetic analysis

(A)

Dataset	Partition	Model of sequence evolution
Sporolithales	*psb*A codon 1	TNe + I + G4
	*psb*A codon 2 + *rbc*L codon 2	TN + F + I
	*psb*A codon 3	TPM3u + F + I + G4
	*rbc*L codon 1	GTR + F + G4
	*rbc*L codon 3	TIM + F + G4
Corallinales	*psb*A codon 1	TNe + I + G4
	*psb*A codon 2	JC + I
	*psb*A codon 3	TIM3 + F + I + G4
	*rbc*L codon 1	GTR + F + I + G4
	*rbc*L codon 2	TIM + F + I + G4
	*rbc*L codon 3	TIM2 + F + I + G4
Hapalidiales	*psb*A codon 1 + *rbc*L Codon 1 + *psb*A codon 3	GTR + F + I + G4
	*psb*A codon 2 + *rbc*L codon 2 + *rbc*L codon 3	TIM + F + I + G4

(B)

Dataset	Partition	Model of sequence evolution
Sporolithales	*psb*A codon 1 + *rbc*L codon 1 + *psb*A codon 3	GTR + I
	*psb*A codon 2 + *rbc*L codon 2 + *rbc*L codon 3	GTR + I + G4
Corallinales	*psb*A codon 1 + *rbc*L codon 1	SYM + I + G4
	*psb*A codon 2 + *rbc*L codon 2 + *psb*A codon 3 + *rbc*L codon 3	GTR + I + G4

Hapalidiales	*psb*A codon 1 + *rbc*L codon 1 + *psb*A codon 3 + *rbc*L codon 3	GTR + I + G4
	*psb*A codon 2 + *rbc*L codon 2	F81 + I + G4
