# Supplementary material for: High diversity of coralline algae in New Zealand revealed: Knowledge gaps and implications for future research
Source: PLoS One. 2019 Dec 2;14(12):e0225645. doi: 10.1371/journal.pone.0225645 (PMC6886753; doi:10.1371/journal.pone.0225645)
Supplement: S3 Table — Species is the phylogenetically derived primary species hypothesis (PSH) the specimen (identified by unique algae number) belongs to. Depth is the metres below mean low water. (PDF) [file pone.0225645.s003.pdf]

S3 Table. Collection data for coralline algae samples successfully amplified with *psb*A gene from the New Zealand region. Species is the phylogenetically derived primary species hypothesis (PSH) the specimen (identified by unique algae number) belongs to. Depth is the metres below mean low water

**Species**	**Order**	**Algae Number**	**psbA GenBank Ascension**	**Location in NZ**	**Lat/Long**	**Depth**	**Morphology**	**Reproduction**
Amphiroa anceps	Corallinales	NZC2207	MK413613	Auckland, Bland Bay	35.346 S 174.367 E	-	Geniculate	-
Amphiroa anceps	Corallinales	NZC2215	FJ361371	Auckland, Rangiatea	35.217 S 174.181 E	13m		-
Amphiroa anceps	Corallinales	NZC2233	FJ361491	Auckland, Okahu Is.	35.201 S 174.205 E	14m	Geniculate	-
Amphiroa anceps	Corallinales	NZC2301	FJ361367	Auckland, Okahu Channel	35.197 S 174.216 E	14m	Geniculate	-
Amphiroa anceps	Corallinales	NZC2322	FJ361594	Auckland, Wekarua Is.	34.936 S 173.654 E	10m	Geniculate	-
Amphiroa anceps	Corallinales	NZC2322	FJ361594	Auckland, Wekarua Is.	34.936 S 173.654 E	10m	Geniculate	-
Amphiroa anceps	Corallinales	NZC2344	FJ361619	Auckland, Pihakoa Point	34.83 S 173.452 E	10m	Geniculate	-
Amphiroa anceps	Corallinales	NZC2361	FJ361601	Auckland, North Cape	34.418 S 173.052 E	8m	-	-
Amphiroa anceps	Corallinales	NZC2384	FJ361587	Auckland, Stephenson Is.	34.97 S 173.79 E	17m	Geniculate	-
Amphiroa anceps	Corallinales	NZC2394	FJ361585	Auckland, Motuharakeke	35.003 S 173.97 E	16m	Geniculate	Uniporates
Arthrocardia sp. A	Corallinales	NZC2088	MK413623	Auckland, Ocean Beach	35.835 S 174.573 E	-	-	-
Arthrocardia sp. A	Corallinales	NZC2157	FJ361466	Auckland, Kiritehere Beach	38.325 S 174.703 E	-	Geniculate	-
Arthrocardia sp. A	Corallinales	NZC2491	FJ361732	Auckland, Ahipara	35.175 S 173.117 E	-	Geniculate	-
Arthrocardia sp. A	Corallinales	NZC2536	FJ361703	Auckland, Kapowairua	34.421 S 172.856 E	-	Geniculate	-
Arthrocardia sp. A	Corallinales	NZC2539	KM369028	Auckland, Kapowairua	34.421 S 172.856 E	-	Geniculate	-
Arthrocardia sp. A	Corallinales	NZC2540	FJ361684	Auckland, Kapowairua	34.421 S 172.856 E	-	Geniculate	-
Arthrocardia sp. B	Corallinales	ASD229	MK413638	Auckland, Kapowairua	34.42 S 172.858 E	-	-	-
Arthrocardia sp. B	Corallinales	NZC2021	FJ361361	Auckland, Te Henga	36.887 S 174.437 E	-	Geniculate	Conceptacle
Arthrocardia sp. B	Corallinales	NZC2343	FJ361618	Auckland, Pihakoa Point	34.83 S 173.452 E	10m	Geniculate	-
Arthrocardia sp. B	Corallinales	NZC2360	FJ361607	Auckland, North Cape	34.418 S 173.052 E	8m	Geniculate	-
Arthrocardia sp. B	Corallinales	NZC2594	FJ361718	Gisborne, Lottin Point	37.553 S 178.164 E	-	Geniculate	-
Arthrocardia sp. B	Corallinales	NZC2598	KM369087	Gisborne, Lottin Point	37.553 S 178.164 E	-	Geniculate	-
Arthrocardia sp. C	Corallinales	ASE107	KM369030	Otago, Kaka Point	-	-	-	-
Arthrocardia sp. C	Corallinales	NZC5029	MK413576	Otago, Butterfly Bay	45.638 S 170.672 E	1m	Geniculate	-
Arthrocardia sp. C	Corallinales	NZC5071	MK413556	Southland, Snout	46.26 S 167.194 E	0m	Geniculate	Conceptacle
Arthrocardia sp. C	Corallinales	NZC5220	MK413505	Otago, Kaka Point, Tawhiri	46.431 S 169.798 E	0m	Geniculate	-
Arthrocardia sp. C	Corallinales	NZC5239	MK413492	Southland, Waikawa, Waipapa	46.661 S 168.846 E	0m	Geniculate	-
Arthrocardia sp. C	Corallinales	NZC5268	MK413473	Otago, Butterfly Bay	45.638 S 170.672 E	1m	Geniculate	Conceptacle
Arthrocardia sp. C	Corallinales	NZC5271	MK413470	Otago, Butterfly Bay	45.638 S 170.672 E	1m	Geniculate	-

Arthrocardia sp. C	Corallinales	NZC5280	MK413466	Otago, Moeraki, Tawhiroko N	45.37 S 170.867 E	0m	Geniculate	Conceptacle
Arthrocardia sp. C	Corallinales	NZC5286	MK413461	Otago, Moeraki, Okahau South	45.382 S 170.868 E	3m	Geniculate	Uniporates
Arthrocardia sp. C	Corallinales	NZC5309A	MK413449	Otago, Moeraki, Katiki Point W	45.397 S 170.868 E	3m	Geniculate	-
Arthrocardia sp. C	Corallinales	NZC5374	MK413407	Stewart Island, Horseshoe Bay	46.878 S 168.148 E	0m	Geniculate	-
Arthrocardia sp. C	Corallinales	NZC5392	MK413397	Stewart Island, Passage Rock	47.078 S 168.205 E	2m	Geniculate	Uniporates- Dome
Arthrocardia sp. C	Corallinales	NZC5424	MK413382	Stewart Is., Black & White Bay	46.692 S 167.888 E	0m	Geniculate	-
Arthrocardia sp. C	Corallinales	NZC5491A	MK413349	Otago, Dundein, Akatore	46.112 S 170.192 E	0m	Geniculate	Conceptacle
Corallina sp.	Corallinales	ASD026	KM369036	Otago, Kaka Point	46.408 S 169.793 E	-	-	-
Corallina sp.	Corallinales	ASD027	DQ168011	Otago, Campbell Point	46.408 S 169.793 E	-	-	-
Corallina sp.	Corallinales	ASD153	DQ167882	Gisborne, Raukokore	37.639 S 177.879 E	-	-	-
Corallina sp.	Corallinales	ASD200	EF628230	Auckland, Te Werahi Beach	34.438 S 172.678 E	-	-	-
Corallina sp.	Corallinales	ASE049	DQ167911	Chatham Island, Wharekauri	43.707 S 176.574 E	-	-	-
Corallina sp.	Corallinales	ASE050	DQ167912	Chatham Island, Wharekauri	43.707 S 176.574 E	-	-	-
Corallina sp.	Corallinales	ASE090	DQ168015	Wellington, Is. Bay	-	-	-	-
Corallina sp.	Corallinales	ASG241	FJ361566	Chatham Island, Wharekauri	-	-	-	-
Corallina sp.	Corallinales	ASG242	FJ361567	Chatham Island, Wharekauri	-	-	-	-
Corallina sp.	Corallinales	ASN188B	MK413636	Auckland Islands, Derry Castle	50.484 S 166.304 E	0m	Geniculate	Conceptacle
Corallina sp.	Corallinales	ASN209	MK413632	Auckland Islands, Derry Castle	50.484 S 166.304 E	0m	Geniculate	-
Corallina sp.	Corallinales	GS8	MK413630	Marlborough, Oaro	42.515 S 173.51 E	0m	-	-
Corallina sp.	Corallinales	NZC0073	DQ167910	Marlborough, Halfmoon Bay	42.267 S 173.8 E	0m	Lumpy	Multiporates
Corallina sp.	Corallinales	NZC2010	FJ361379	Auckland, Little Huia	37.011 S 174.562 E	-	Geniculate	Uniporates
Corallina sp.	Corallinales	NZC2038	FJ361399	Auckland, Rocky Bay	36.819 S 175.053 E	-	Geniculate	-
Corallina sp.	Corallinales	NZC2060	MK413624	Auckland, Mathesons Bay	36.302 S 174.798 E	-	Lumpy	Uniporates
Corallina sp.	Corallinales	NZC2067	FJ361417	Auckland, Lang's Beach	36.043 S 174.532 E	-	Geniculate	-
Corallina sp.	Corallinales	NZC2068	FJ361447	Auckland, Lang's Beach	36.043 S 174.532 E	-	Geniculate	-
Corallina sp.	Corallinales	NZC2069	FJ361413	Auckland, Lang's Beach	36.043 S 174.532 E	-	Geniculate	-
Corallina sp.	Corallinales	NZC2075	FJ361429	Auckland, Ngunguru Harbour	35.636 S 174.5 E	-	Geniculate	-
Corallina sp.	Corallinales	NZC2082	FJ361553	Auckland, Sandy Bay	35.557 S 174.479 E	-	Geniculate	-
Corallina sp.	Corallinales	NZC2086	FJ361451	Auckland, Ocean Beach	35.835 S 174.573 E	-	Geniculate	-
Corallina sp.	Corallinales	NZC2097	MK413622	Auckland, Opononi	35.518 S 173.388 E	-	Encrusting	Uniporates
Corallina sp.	Corallinales	NZC2098	MK413621	Auckland, Opononi	35.518 S 173.388 E	-	-	Calcified Compartments
Corallina sp.	Corallinales	NZC2102	FJ361431	Auckland, Opononi	35.518 S 173.388 E	-	Geniculate	-
Corallina sp.	Corallinales	NZC2113	KM369037	Auckland, Matai Bay	34.828 S 173.411 E	-	Geniculate	Uniporates
Corallina sp.	Corallinales	NZC2118	FJ361416	Auckland, Matai Bay	34.828 S 173.411 E	-	Geniculate	-

Corallina sp.	Corallinales	NZC2123	FJ361415	Auckland, Rangiputa	34.88 S 173.289 E	-	Geniculate	-
Corallina sp.	Corallinales	NZC2126	MK413620	Auckland, Matauri Bay	35.027 S 173.914 E	-	Layered	Uniporates
Corallina sp.	Corallinales	NZC2129	FJ361449	Auckland, Matauri Bay	35.027 S 173.914 E	-	Encrusting	Multiporates-Volcano
Corallina sp.	Corallinales	NZC2131	FJ361464	Auckland, Matauri Bay	35.027 S 173.914 E	-	Geniculate	-
Corallina sp.	Corallinales	NZC2136	FJ361565	Auckland, Cable Bay	34.99 S 173.48 E	-	Geniculate	-
Corallina sp.	Corallinales	NZC2150	FJ361564	Taranaki, Waitara	38.988 S 174.219 E	-	-	-
Corallina sp.	Corallinales	NZC2158	FJ361472	Auckland, Kiritehere Beach	38.325 S 174.703 E	-	Geniculate	-
Corallina sp.	Corallinales	NZC2171	FJ361473	Auckland, Whale Bay	37.822 S 174.802 E	-		-
Corallina sp.	Corallinales	NZC2179	FJ361510	Auckland, Waitata Rocks	35.256 S 174.132 E	-		-
Corallina sp.	Corallinales	NZC2196	FJ361532	Auckland, Bland Bay	35.346 S 174.367 E	-	-	-
Corallina sp.	Corallinales	NZC2217	FJ361496	Auckland, Rangiatea	35.217 S 174.181 E	13m		-
Corallina sp.	Corallinales	NZC2219	FJ361521	Auckland, Rangiatea	35.217 S 174.181 E	13m		-
Corallina sp.	Corallinales	NZC2231	FJ361490	Auckland, Okahu Is.	35.201 S 174.205 E	14m	-	-
Corallina sp.	Corallinales	NZC2232	FJ361485	Auckland, Moturoa & Motu channel	35.213 S 174.197 E	14m		-
Corallina sp.	Corallinales	NZC2293	KM369038	Auckland, Urupukapuka	35.205 S 174.234 E	14m	Geniculate	-
Corallina sp.	Corallinales	NZC2294	FJ361499	Auckland, Okahu Channel	35.197 S 174.216 E	14m	Geniculate	-
Corallina sp.	Corallinales	NZC2296	FJ361537	Auckland, Urupukapuka	35.205 S 174.234 E	14m	Geniculate	-
Corallina sp.	Corallinales	NZC2297	FJ361526	Auckland, Okahu Channel	35.197 S 174.216 E	14m	Geniculate	-
Corallina sp.	Corallinales	NZC2298	FJ361479	Auckland, Okahu Channel	35.197 S 174.216 E	14m	Geniculate	-
Corallina sp.	Corallinales	NZC2324	FJ361588	Auckland, Wekarua Is.	34.936 S 173.654 E	10m	Geniculate	-
Corallina sp.	Corallinales	NZC2333	FJ361591	Auckland, Wekarua Is.	34.936 S 173.654 E	10m	Geniculate	Uniporates
Corallina sp.	Corallinales	NZC2362	FJ361604	Auckland, North Cape	34.418 S 173.052 E	8m	Geniculate	Uniporates
Corallina sp.	Corallinales	NZC2383	FJ361582	Auckland, Stephenson Is.	34.97 S 173.79 E	17m	Geniculate	-
Corallina sp.	Corallinales	NZC2390	FJ361580	Auckland, Motuharakeke	35.003 S 173.97 E	5m	Geniculate	-
Corallina sp.	Corallinales	NZC2397	FJ361593	Auckland, Haraweka Is.	34.98 S 173.952 E	9m	Geniculate	-
Corallina sp.	Corallinales	NZC2399	FJ361579	Auckland, Urupukapuka	35.221 S 174.24 E	5m	Geniculate	-
Corallina sp.	Corallinales	NZC2470	FJ361645	Auckland, Fletcher Bay	36.475 S 175.392 E	-	Geniculate	-
Corallina sp.	Corallinales	NZC2473	FJ361651	Auckland, Fletcher Bay	36.475 S 175.392 E	-	Geniculate	-
Corallina sp.	Corallinales	NZC2482	FJ361627	Auckland, Wilson Bay	36.887 S 175.426 E	-	Geniculate	-
Corallina sp.	Corallinales	NZC2493	FJ361731	Auckland, Ahipara	35.175 S 173.117 E	-	Geniculate	Uniporates
Corallina sp.	Corallinales	NZC2496	KM369039	Auckland, Ahipara	35.175 S 173.117 E	-	Geniculate	Uniporates
Corallina sp.	Corallinales	NZC2525	FJ361733	Auckland, Henderson Point	34.741 S 173.118 E	-	Geniculate	Uniporates
Corallina sp.	Corallinales	NZC2527	FJ361742	Auckland, Henderson Point	34.741 S 173.118 E	-	Geniculate	-
Corallina sp.	Corallinales	NZC2537	KM369094	Auckland, Kapowairua	34.421 S 172.856 E	-	Geniculate	Uniporates

Corallina sp.	Corallinales	NZC2555	FJ361747	Auckland, The Bluff	34.685 S 172.89 E	-	Geniculate	Uniporates
Corallina sp.	Corallinales	NZC2566	MK413599	Auckland, Tapotupotu Bay	34.435 S 172.717 E	-	Geniculate	Uniporates
Corallina sp.	Corallinales	NZC2566	MK413599	Auckland, Tapotupotu Bay	34.435 S 172.717 E	-	Geniculate	Uniporates
Corallina sp.	Corallinales	NZC2567	FJ361726	Auckland, Tapotupotu Bay	34.435 S 172.717 E	-	Geniculate	Uniporates
Corallina sp.	Corallinales	NZC2569	FJ361701	Auckland, Tapotupotu Bay	34.435 S 172.717 E	-	Geniculate	Uniporates
Corallina sp.	Corallinales	NZC2571	FJ361687	Auckland, Tapotupotu Bay	34.435 S 172.717 E	-	Geniculate	Uniporates
Corallina sp.	Corallinales	NZC2596	FJ361717	Gisborne, Lottin Point	37.553 S 178.164 E	-	Geniculate	Uniporates
Corallina sp.	Corallinales	NZC2599	FJ361721	Gisborne, Lottin Point	37.553 S 178.164 E	-	Geniculate	Uniporates
Corallina sp.	Corallinales	NZC4003	MK413593	Antipodes Islands, Anchorage	-	-	Geniculate	-
Corallina sp.	Corallinales	NZC5001	MK413589	Otago, Karitane	45.642 S 170.678 E	10m	Geniculate	-
Corallina sp.	Corallinales	NZC5004	MK413587	Otago, Murdering Beach West	45.761 S 170.666 E	0m	Geniculate	-
Corallina sp.	Corallinales	NZC5005	MK413586	Otago, Murdering Beach West	45.761 S 170.666 E	0m	Geniculate	-
Corallina sp.	Corallinales	NZC5017	MK413583	Otago, Brighton	45.949 S 170.335 E	0m	Geniculate	-
Corallina sp.	Corallinales	NZC5030	MK413575	Otago, Butterfly Bay	45.638 S 170.672 E	1m	Geniculate	-
Corallina sp.	Corallinales	NZC5032	MK413574	Otago, Butterfly Bay	45.638 S 170.672 E	1m	Geniculate	-
Corallina sp.	Corallinales	NZC5044	MK413570	Otago, Wellers Rock	45.798 S 170.715 E	0m	Geniculate	-
Corallina sp.	Corallinales	NZC5055A	MK413565	Southland, Crombie	46.257 S 167.164 E	0m	Geniculate	-
Corallina sp.	Corallinales	NZC5055B	MK413564	Southland, Crombie	46.257 S 167.164 E	0m	Geniculate	-
Corallina sp.	Corallinales	NZC5055C	MK413563	Southland, Crombie	46.257 S 167.164 E	0m	Geniculate	-
Corallina sp.	Corallinales	NZC5131	MK413537	Fiordland, Anchor Island North	45.761 S 166.537 E	5m	Geniculate	-
Corallina sp.	Corallinales	NZC5134	MK413536	Fiordland, Five Fingers Pen.	45.712 S 166.5 E	11m	Geniculate	-
Corallina sp.	Corallinales	NZC5151	MK413527	Auckland Islands, Shag Rock	50.71 S 166.201 E	0m	Geniculate	-
Corallina sp.	Corallinales	NZC5158	MK413523	Fiordland, Turn Round Point	44.8 S 167.544 E	5m	Geniculate	-
Corallina sp.	Corallinales	NZC5161	MK413521	Fiordland, Five Fingers Pen.	45.712 S 166.5 E	6m	Geniculate	-
Corallina sp.	Corallinales	NZC5166	MK413520	Fiordland, Whidby Point	45.698 S 166.547 E	5m	Geniculate	Conceptacle
Corallina sp.	Corallinales	NZC5203	MK413513	Fiordland, Sunday Cove	45.594 S 166.741 E	1m	Geniculate	-
Corallina sp.	Corallinales	NZC5206	MK413511	Fiordland, Sunday Cove	45.594 S 166.741 E	1m	Geniculate	-
Corallina sp.	Corallinales	NZC5208	MK413509	Fiordland, Sunday Cove	45.594 S 166.741 E	1m	Geniculate	Conceptacle
Corallina sp.	Corallinales	NZC5233	MK413495	Southland, Waikawa, Waipapa	46.661 S 168.846 E	0m	Geniculate	-
Corallina sp.	Corallinales	NZC5246	MK413488	Southland, Waikawa, Curio Bay	46.662 S 169.103 E	0m	Geniculate	-
Corallina sp.	Corallinales	NZC5261	MK413477	Southland, Riverton, Cosy Nook	46.331 S 167.72 E	0m	Geniculate	-
Corallina sp.	Corallinales	NZC5273	MK413469	Otago, Moeraki, Tawhiroko N	45.367 S 170.866 E	0m	Geniculate	Conceptacle
Corallina sp.	Corallinales	NZC5284	MK413463	Otago, Moeraki, Seal Nook	45.386 S 170.868 E	0m	Geniculate	-
Corallina sp.	Corallinales	NZC5302	MK413452	Otago, Moeraki, Paitu Head	45.36 S 170.864 E	3m	Geniculate	-

Corallina sp.	Corallinales	NZC5319	MK413442	Westland, Jackson Bay	43.972 S 168.616 E	0m	Geniculate	-
Corallina sp.	Corallinales	NZC5321	MK413441	Westland, Jackson Bay	43.972 S 168.616 E	0m	Geniculate	-
Corallina sp.	Corallinales	NZC5343	MK413422	Westland, Ocean Beach	43.966 S 168.607 E	0m	Geniculate	-
Corallina sp.	Corallinales	NZC5348	MK413418	Otago, Chaslands	46.625 S 169.361 E	2m	Geniculate	Conceptacle
Corallina sp.	Corallinales	NZC5360	MK413415	Otago, Chaslands	46.625 S 169.361 E	0m	Geniculate	Conceptacle
Corallina sp.	Corallinales	NZC5365	MK413411	Stewart Island, Horseshoe Bay	46.878 S 168.148 E	2m	Geniculate	-
Corallina sp.	Corallinales	NZC5422	MK413383	Stewart Is., Black & White Bay	46.692 S 167.888 E	0m	Geniculate	Conceptacle
Corallina sp.	Corallinales	NZC5481	MK413356	Otago, Dundein, Akatore	46.112 S 170.192 E	0m	Geniculate	Conceptacle
Corallina sp.	Corallinales	NZC5482	MK413355	Otago, Dundein, Akatore	46.112 S 170.192 E	0m	Geniculate	Conceptacle
Corallina sp.	Corallinales	NZC5488	MK413350	Otago, Dundein, Akatore	46.112 S 170.192 E	0m	Geniculate	-
Corallina sp.	Corallinales	NZC5507	MK413338	Otago, Green Island	45.952 S 170.386 E	0m	Geniculate	-
Corallina sp.	Corallinales	NZC5529	MK413330	Otago, Dunedin, Quarantine Is.	45.828 S 170.637 E	0m	Geniculate	-
Corallina sp.	Corallinales	NZC5657	MK413240	Fiordland, Straggle Head	46.032 S 166.546 E	1m	Geniculate	Conceptacle
Corallina sp.	Corallinales	NZC5667	MK413232	Fiordland, Halt Bay	46.028 S 166.547 E	0m	Geniculate	Conceptacle
Corallinales sp. A	Corallinales	NZC5252	MK413483	Southland, Riverton, Monkey Is.	46.3 S 167.725 E	0m	Encrusting	Uniporates- Dome
Corallinales sp. A	Corallinales	NZC5546	MK413318	Otago, Moeraki	45.362 S 170.863 E	1m	Warty	Uniporates- Dome
Corallinales sp. A	Corallinales	NZC5719	MK413213	Fiordland, North Port	46.035 S 166.592 E	1m	Warty	Uniporates- Flush
Corallinales sp. B	Corallinales	NZC5472	MK413358	Southland, Bluff, site 24028	46.583 S 168.329 E	6m	Encrusting	Uniporates- Dome
Corallinales sp. B	Corallinales	NZC5685	MK413224	Fiordland, Little Island	45.973 S 166.589 E	5m	Warty- Epizoic	Uniporates- Dome
Corallinales sp. C	Corallinales	NZC2266	FJ361535	Auckland, Moturoa & Motu channel	35.213 S 174.197 E	14m	Encrusting	Uniporates
Corallinales sp. C	Corallinales	NZC2547	KM369040	Auckland, Kapowairua	34.421 S 172.856 E	-	Encrusting	Uniporates
Corallinales sp. D	Corallinales	NZC5138	MK413535	Auckland Islands, Smiths Harbour	50.706 S 166.131 E	0m	Epilithic	Uniporates
Corallinales sp. E	Corallinales	NZC5257	MK413481	Southland, Riverton, Cosy Nook	46.331 S 167.72 E	0m	Encrusting	Uniporates- Flush
Corallinales sp. E	Corallinales	NZC5282	MK413464	Otago, Moeraki, Tawhiroko N	45.37 S 170.867 E	0m	Encrusting	Uniporates
Corallinales sp. E	Corallinales	NZC5484	MK413353	Otago, Dundein, Akatore	46.112 S 170.192 E	0m	Encrusting	Uniporates- Dome
Corallinales sp. F	Corallinales	NZC2025	FJ361391	Auckland, ManOWar Bay	36.786 S 175.157 E	-	Encrusting	Uniporates
Corallinales sp. F	Corallinales	NZC2027	FJ361392	Auckland, ManOWar Bay	36.786 S 175.157 E	-	Epizoic	Uniporates
Corallinales sp. F	Corallinales	NZC2080	FJ361458	Auckland, Tutukaka	35.612 S 174.532 E	-	Encrusting	Uniporates
Corallinales sp. F	Corallinales	NZC2100	FJ361439	Auckland, Opononi	35.518 S 173.388 E	-	Epilithic	Uniporates
Corallinales sp. F	Corallinales	NZC2101	FJ361437	Auckland, Opononi	35.518 S 173.388 E	-	Encrusting	Uniporates
Corallinales sp. F	Corallinales	NZC2446	FJ361657	Auckland, Little Bay	36.601 S 175.549 E	-	Encrusting	Uniporates
Corallinales sp. G	Corallinales	NZC0751	DQ167933	Chatham Island, Port Hutt	43.816 S 176.705 W	-	Encrusting	Uniporates
Corallinales sp. G	Corallinales	NZC0751	DQ167933	Chatham Island, Port Hutt	43.816 S 176.705 W	-	Encrusting	Uniporates
Corallinales sp. G	Corallinales	NZC2009	KM369046	Auckland, Little Huia	37.011 S 174.562 E	-	Encrusting	Uniporates

Corallinales sp. G	Corallinales	NZC2120	FJ361410	Auckland, Rangiputa	34.88 S 173.289 E	-	Warty	Uniporates
Corallinales sp. G	Corallinales	NZC2140	FJ361445	Auckland, Cable Bay	34.99 S 173.487 E	-	Encrusting	Uniporates
Corallinales sp. G	Corallinales	NZC2500	FJ361755	Auckland, Ahipara	35.175 S 173.117 E	-	Encrusting	Uniporates
Corallinales sp. H	Corallinales	NZC5378	MK413405	Stewart Island, Horseshoe Bay	46.878 S 168.148 E	0m	Encrusting	Uniporates- Dome
Corallinales sp. I	Corallinales	NZC5243	MK413490	Southland, Waikawa, Waipapa	46.661 S 168.846 E	0m	Fruticose	Uniporates- Dome
Corallinales sp. I	Corallinales	NZC5375	MK413406	Stewart Island, Horseshoe Bay	46.878 S 168.148 E	0m	Warty	-
Corallinales sp. I	Corallinales	NZC5402	MK413392	Stewart Island, Tikotatahi	47.087 S 168.152 E	0m	Fruticose	Uniporates- Dome
Corallinales sp. I	Corallinales	NZC5633	MK413248	Fiordland, Spit Islands	46.072 S 166.634 E	0m	Fruticose	Uniporates- Dome
Corallinales sp. I	Corallinales	NZC5671	MK413230	Fiordland, Halt Bay	46.028 S 166.547 E	0m	Warty	Uniporates- Dome
Corallinales sp. J	Corallinales	NZC5021	MK413581	Otago, Brighton	45.949 S 170.335 E	0m	Encusting	-
Corallinales sp. J	Corallinales	NZC5217	MK413507	Otago, Kaka Point, Tawhiri	46.431 S 169.798 E	0m	Epizoic	Uniporates- Dome
Corallinales sp. J	Corallinales	NZC5232A	MK413497	Southland, Waikawa, Waipapa	46.661 S 168.846 E	0m	Encrusting- Epizoic	Uniporates
Corallinales sp. J	Corallinales	NZC5249	MK413486	Southland, Riverton, Monkey Is.	46.3 S 167.725 E	0m	Epizoic	Uniporates- Dome
Corallinales sp. J	Corallinales	NZC5314	MK413447	Westland, Jackson Bay	43.972 S 168.616 E	0m	Warty	Uniporates- Dome
Corallinales sp. J	Corallinales	NZC5332	MK413432	Westland, Ocean Beach	43.966 S 168.607 E	0m	Lumpy	-
Corallinales sp. J	Corallinales	NZC5532	MK413329	Otago, Dunedin, Quarantine Is.	45.828 S 170.637 E	0m	Warty	Uniporates- Dome
Corallinales sp. K	Corallinales	NZC0676	DQ168007	Hawkes Bay, Mangakuri Beach	39.966 S 176.922 E	0m	Encrusting	Uniporates
Corallinales sp. K	Corallinales	NZC2412	FJ361739	Auckland, Sailors Grave	36.961 S 175.844 E	-	Epiphytic	Uniporates
Corallinales sp. L	Corallinales	NZC0482	EF628236	Nelson, Mussel Farm Bay	40.807 S 172.929 E	-	Encrusting	Uniporates
Corallinales sp. L	Corallinales	NZC0488	DQ167896	Nelson, Mussel Farm Bay	40.807 S 172.929 E	-	Epizoic	Multiporates
Corallinales sp. L	Corallinales	NZC0510	DQ167900	Nelson, Mussel Farm Bay	40.807 S 172.929 E	-	Epizoic	Uniporates
Corallinales sp. L	Corallinales	NZC0516	DQ167902	Nelson, Mussel Farm Bay	40.807 S 172.929 E	0m	Encrusting	Uniporates
Corallinales sp. L	Corallinales	NZC2071	FJ361411	Auckland, Lang's Beach	36.043 S 174.532 E	-	Encrusting	-
Corallinales sp. L	Corallinales	NZC2074	FJ361414	Auckland, Lang's Beach	36.043 S 174.532 E	-	Encrusting	Uniporates
Corallinales sp. L	Corallinales	NZC2125	KM369107	Auckland, Matauri Bay	35.027 S 173.914 E	-	Warty	Uniporates
Corallinales sp. L	Corallinales	NZC2511	FJ361699	Auckland, Henderson Point	34.741 S 173.118 E	-	Encrusting	Uniporates
Corallinales sp. M	Corallinales	NZC5333	MK413431	Westland, Ocean Beach	43.966 S 168.607 E	0m	Encrusting	Uniporates- Dome
Corallinales sp. M	Corallinales	NZC5750D	MK413203	Otago, Butterfly Bay	45.638 S 170.672 E	10m	Epilithic	-
Corallinales sp. N	Corallinales	NZC0742	DQ167926	Chatham Island, Waitangi	43.785 S 176.812 W	-	Encrusting	Uniporates
Corallinales sp. N	Corallinales	NZC0749	DQ168003	Chatham Island, Port Hutt	43.816 S 176.705 W	-	Warty	Uniporates
Corallinales sp. N	Corallinales	NZC0761	DQ167940	Chatham Island, Point Durham	44 S 176.675 W	-	Encrusting	-
Corallinales sp. N	Corallinales	NZC0767	DQ167954	Chatham Island, Okawa Point	43.77 S 176.247 W	-	Encrusting	Uniporates
Corallinales sp. N	Corallinales	NZC0777	KM369047	Chatham Island, Te One Creek	44.019 S 176.383 W	-	Encrusting	Multiporates
Corallinales sp. N	Corallinales	NZC0778	DQ167952	Chatham Island, Tommy Solomon Monument	44.032 S 176.337 W	0m	Encrusting	Multiporates

Corallinales sp. N	Corallinales	NZC0781	EF628238	Chatham Island, Heaphy Shoal	43.966 S 176.593 W	-	Encrusting	Uniporates
Corallinales sp. O	Corallinales	NZC2062	FJ361460	Auckland, Chucks Cove	34.99 S 173.489 E	-	Encrusting	Uniporates
Corallinales sp. O	Corallinales	NZC2091	FJ361463	Auckland, Ocean Beach	35.835 S 174.573 E	-	Encrusting	Uniporates
Corallinales sp. O	Corallinales	NZC2107	FJ361448	Auckland, Matai Bay	34.828 S 173.411 E	-	-	Uniporates
Corallinales sp. O	Corallinales	NZC2122	KM369042	Auckland, Rangiputa	34.88 S 173.289 E	-	Encrusting	Uniporates
Corallinales sp. O	Corallinales	NZC2163	FJ361563	Auckland, Kiritehere Beach	38.325 S 174.703 E	-	Encrusting	Uniporates
Corallinales sp. O	Corallinales	NZC2460	FJ361643	Auckland, Fletcher Bay	36.475 S 175.392 E	-	Encrusting	Uniporates
Corallinales sp. O	Corallinales	NZC2477	FJ361628	Auckland, Wilson Bay	36.887 S 175.426 E	-	Encrusting	Uniporates
Corallinales sp. O	Corallinales	NZC2498	FJ361743	Auckland, Ahipara	35.175 S 173.117 E	-	Encrusting	Uniporates
Corallinales sp. P	Corallinales	ASE303	DQ168019	Stewart Island, Port William	46.845 S 168.083 E	-	-	-
Corallinales sp. P	Corallinales	NZC0076	DQ167988	Marlborough, Oaro Reef	42.515 S 173.51 E	-	Encrusting	Uniporates
Corallinales sp. P	Corallinales	NZC0090	EF628237	Marlborough, Halfmoon Bay	42.267 S 173.8 E	0m	Encrusting	Uniporates
Corallinales sp. P	Corallinales	NZC0096	DQ167878	Marlborough, Rakautara Stream	42.27 S 173.8 E	-	Epilithic	Multiporates
Corallinales sp. P	Corallinales	NZC5050	MK413567	Southland, River Mouth	46.261 S 167.204 E	0m	Warty	Uniporates- Flush
Corallinales sp. P	Corallinales	NZC5059	MK413561	Southland, Crombie	46.257 S 167.164 E	0m	Warty	Uniporates- Flush
Corallinales sp. P	Corallinales	NZC5059	MK413561	Southland, Crombie	46.257 S 167.164 E	0m	Warty	Uniporates- Flush
Corallinales sp. P	Corallinales	NZC5059	MK413561	Southland, Crombie	46.257 S 167.164 E	0m	Warty	Uniporates- Flush
Corallinales sp. P	Corallinales	NZC5059	MK413561	Southland, Crombie	46.257 S 167.164 E	0m	Warty	Uniporates- Flush
Corallinales sp. P	Corallinales	NZC5251A	MK413485	Southland, Riverton, Monkey Is.	46.3 S 167.725 E	0m	Epizoic	Uniporates- Flush
Corallinales sp. P	Corallinales	NZC5317	MK413444	Westland, Jackson Bay	43.972 S 168.616 E	0m	Lumpy	Uniporates- Flush
Corallinales sp. P	Corallinales	NZC5334	MK413430	Westland, Ocean Beach	43.966 S 168.607 E	0m	Lumpy	-
Corallinales sp. P	Corallinales	NZC5411	MK413389	Stewart Island, Tikotatahi	47.087 S 168.152 E	0m	Encrusting	Uniporates- Dome
Corallinales sp. P	Corallinales	NZC5430	MK413376	Stewart Is., Black & White Bay	46.692 S 167.888 E	2m	Encrusting	Uniporates- Flush
Corallinales sp. P	Corallinales	NZC5485	MK413352	Otago, Dundein, Akatore	46.112 S 170.192 E	0m	Lumpy- Epizoic	Uniporates- Flush
Corallinales sp. P	Corallinales	NZC5670	MK413231	Fiordland, Halt Bay	46.028 S 166.547 E	0m	Warty	Uniporates- Flush
Corallinales sp. Q	Corallinales	NZC0667	EF628239	Hawkes Bay, Tuingara Point	40.121 S 176.875 E	0m	Encrusting	Uniporates
Corallinales sp. Q	Corallinales	NZC0674	DQ168006	Hawkes Bay, Tuingara Point	40.121 S 176.875 E	-	Encrusting	Uniporates
Corallinales sp. R	Corallinales	NZC2328	FJ361598	Auckland, Wekarua Is.	34.936 S 173.654 E	10m	Epiphytic	Uniporates
Corallinales sp. R	Corallinales	NZC2487	KM369051	Auckland, Pihakoa Point	34.83 S 173.452 E	10m	Epizoic	Uniporates
Corallinales sp. S	Corallinales	ASN051	MK413637	Campbell Island, Beeman Point	52.552 S 169.151 E	0m	Encrusting	-
Corallinales sp. S	Corallinales	ASN200	MK413633	Auckland Islands, Derry Castle	50.484 S 166.304 E	0m	Encrusting	Uniporates- Dome
Corallinales sp. S	Corallinales	NZC4007	MK413591	Bounty Is., South Tunnel Is.	-	12m	Encrusting	Uniporates- Flush
Corallinales sp. T	Corallinales	NZC2309	FJ361370	Auckland, Urupukapuka	35.205 S 174.234 E	14m	Fruticose	Uniporates
Corallinales sp. T	Corallinales	NZC2418	FJ361375	Auckland, Sailors Grave	36.961 S 175.844 E	-	Fruticose	Calcified Compartments

Corallinales sp. T	Corallinales	NZC2545	KM369052	Auckland, Kapowairua	34.421 S 172.856 E	-	Foliose	Uniporates
Corallinales sp. T	Corallinales	NZC2550	FJ361691	Auckland, Kapowairua	34.421 S 172.856 E	-	Warty	Uniporates
Corallinales sp. T	Corallinales	NZC2573	MK413598	Auckland, Tapotupotu Bay	34.435 S 172.717 E	-	Fruticose	Uniporates
Corallinales sp. T	Corallinales	NZC2578	FJ361714	Auckland, Tapotupotu Bay	34.435 S 172.717 E	-	Fruticose	Uniporates
Corallinales sp. T	Corallinales	NZC2583	FJ361727	Auckland, Tapotupotu Bay	34.435 S 172.717 E	-	Encrusting	Uniporates
Corallinales sp. T	Corallinales	NZC2597	MK413596	Gisborne, Lottin Point	37.553 S 178.164 E	-	Geniculate	Uniporates
Corallinales sp. T	Corallinales	NZC2605	FJ361694	Gisborne, Lottin Point	37.553 S 178.164 E	-	Fruticose	Uniporates
Corallinales sp. U	Corallinales	NZC0445	DQ167895	Nelson, Wharariki Beach	40.5 S 172.676 E	-	Encrusting	Uniporates
Corallinales sp. U	Corallinales	NZC0769	DQ167963	Chatham Island, Okawa Point	43.77 S 176.247 W	-	Lumpy	Uniporates
Corallinales sp. U	Corallinales	NZC2046	FJ361408	Auckland, Jones Bay	36.377 S 174.824 E	-	Warty	Uniporates
Corallinales sp. U	Corallinales	NZC2055	KM369057	Auckland, Jones Bay	36.377 S 174.824 E	-	Lumpy	Uniporates
Corallinales sp. U	Corallinales	NZC2083	FJ361407	Auckland, Sandy Bay	35.557 S 174.479 E	-	Encrusting	Uniporates
Corallinales sp. V	Corallinales	NZC2058	FJ361409	Auckland, Mathesons Bay	36.302 S 174.798 E	-	Fruticose	Uniporates
Corallinales sp. V	Corallinales	NZC2191	FJ361528	Auckland, Tapeka Beach	35.243 S 174.118 E	-		-
Corallinales sp. V	Corallinales	NZC2250	KM369056	Auckland, Rangiatea	35.217 S 174.181 E	13m	Encrusting	Uniporates
Corallinales sp. V	Corallinales	NZC2417	FJ361664	Auckland, Sailors Grave	36.961 S 175.844 E	-	Foliose	Uniporates
Corallinales sp. V	Corallinales	NZC2464	FJ361644	Auckland, Fletcher Bay	36.475 S 175.392 E	-	Encrusting	Uniporates
Corallinales sp. V	Corallinales	NZC2522	FJ361745	Auckland, Henderson Point	34.741 S 173.118 E	-	Fruticose	Uniporates
Corallinales sp. V	Corallinales	NZC2523	FJ361695	Auckland, Henderson Point	34.741 S 173.118 E	-	Warty	Uniporates
Corallinales sp. V	Corallinales	NZC2576	FJ361678	Auckland, Tapotupotu Bay	34.435 S 172.717 E	-	Lumpy	Uniporates
Corallinales sp. W	Corallinales	NZC5673	MK413229	Fiordland, Halt Bay	46.028 S 166.547 E	0m	Discoid- Epiphytic	Uniporates- Dome
Corallinales sp. X	Corallinales	NZC0314	EF628240	Nelson, Wharariki Beach	40.5 S 172.677 E	-	Encrusting	Uniporates
Corallinales sp. X	Corallinales	NZC5331	MK413433	Westland, Jackson Bay	43.972 S 168.616 E	0m	Discoid- Epiphytic	Uniporates- Pointy
Corallinales sp. Y	Corallinales	DH3	DQ167941	Northland	-	-	-	-
Corallinales sp. Y	Corallinales	NZC2105	FJ361442	Auckland, Matai Bay	34.828 S 173.411 E	-	Lumpy	Uniporates
Corallinales sp. Y	Corallinales	NZC2143	FJ361749	Auckland, Cable Bay	34.99 S 173.487 E	-	Epiphytic	Uniporates
Corallinales sp. Y	Corallinales	NZC2220	FJ361481	Auckland, Rangiatea	35.217 S 174.181 E	13m		Uniporates
Corallinales sp. Y	Corallinales	NZC2302	FJ361514	Auckland, Urupukapuka	35.205 S 174.234 E	14m	Epiphytic- Foliose	Uniporates
Corallinales sp. Y	Corallinales	NZC2345	FJ361602	Auckland, Pihakoa Point	34.83 S 173.452 E	10m	Epiphytic- Foliose	Uniporates
Corallinales sp. Y	Corallinales	NZC2380	FJ361641	Auckland, Stephenson Is.	34.97 S 173.79 E	17m	Epiphytic	Uniporates
Corallinales sp. Y	Corallinales	NZC2489	FJ361753	Auckland, Ahipara	35.175 S 173.117 E	-	Epiphytic- Foliose	Uniporates
Corallinales sp. Y	Corallinales	NZC2505	KM369053	Auckland, Henderson Point	34.741 S 173.118 E	-	Epiphytic- Foliose	Uniporates
Corallinales sp. Y	Corallinales	NZC2529	FJ361709	Auckland, Kapowairua	34.421 S 172.856 E	-	Epiphytic- Foliose	Uniporates
Corallinales sp. Y	Corallinales	NZC2561	FJ361675	Auckland, Tapotupotu Bay	34.435 S 172.717 E	-	Epiphytic- Foliose	Uniporates

Corallinales sp. Y	Corallinales	NZC2591	FJ361729	Gisborne, Lottin Point	37.553 S 178.164 E	-	Epiphytic- Foliose	Uniporates
Corallinales sp. Z	Corallinales	NZC5418	MK413384	Stewart Island, Tikotatahi	47.087 S 168.152 E	2m	Epilithic	Uniporates- Dome
Corallinales sp. ZA	Corallinales	NZC0870	DQ167984	Wellington, Ranger Point	41.34 S 174.825 E	0m	Epiphytic	Uniporates
Corallinales sp. ZA	Corallinales	NZC2115	KM369054	Auckland, Matai Bay	34.828 S 173.411 E	-	Epiphytic- Foliose	Uniporates
Corallinales sp. ZA	Corallinales	NZC2124	FJ361555	Auckland, Matauri Bay	35.027 S 173.914 E	-	Epiphytic- Foliose	Uniporates
Corallinales sp. ZA	Corallinales	NZC2214	FJ361541	Auckland, Rangiatea	35.217 S 174.181 E	13m	Epiphytic	Uniporates
Corallinales sp. ZA	Corallinales	NZC2273	MK413611	Auckland, Okahu Is.	35.201 S 174.205 E	14m	Encrusting	Calcified Compartments
Corallinales sp. ZA	Corallinales	NZC2303	FJ361516	Auckland, Urupukapuka	35.205 S 174.234 E	14m	Epiphytic- Foliose	Uniporates
Corallinales sp. ZA	Corallinales	NZC2341	FJ361590	Auckland, Wekarua Is.	34.936 S 173.654 E	10m	Epiphytic- Foliose	Uniporates
Corallinales sp. ZA	Corallinales	NZC2352	FJ361617	Auckland, Pihakoa Point	34.83 S 173.452 E	10m	Epiphytic- Foliose	Uniporates
Corallinales sp. ZA	Corallinales	NZC2367	FJ361603	Auckland, North Cape	34.418 S 173.052 E	8m	Epiphytic- Foliose	Uniporates
Corallinales sp. ZA	Corallinales	NZC2452	FJ361646	Auckland, Fletcher Bay	36.475 S 175.392 E	-	Epiphytic- Foliose	Uniporates
Corallinales sp. ZA	Corallinales	NZC2503	FJ361736	Auckland, Henderson Point	34.741 S 173.118 E	-	Epiphytic- Foliose	Uniporates
Corallinales sp. ZA	Corallinales	NZC2528	FJ361706	Auckland, Kapowairua	34.421 S 172.856 E	-	Epiphytic- Foliose	Uniporates
Corallinales sp. ZA	Corallinales	NZC2560	MK413601	Auckland, Tapotupotu Bay	34.435 S 172.717 E	-	Epiphytic- Foliose	Uniporates
Corallinales sp. ZA	Corallinales	NZC2589	FJ361716	Gisborne, Lottin Point	37.553 S 178.164 E	-	Epiphytic- Foliose	Uniporates
Corallinales sp. ZB	Corallinales	NZC2270	FJ361531	Auckland, Okahu Is.	35.201 S 174.205 E	14m	Encrusting	Uniporates
Corallinales sp. ZB	Corallinales	NZC2291	FJ361561	Auckland, Okahu Channel	35.197 S 174.216 E	14m	Encrusting	Uniporates
Corallinales sp. ZC	Corallinales	NZC2340	FJ361592	Auckland, Wekarua Is.	34.936 S 173.654 E	10m	Lumpy	Uniporates
Corallinales sp. ZC	Corallinales	NZC2409	FJ361581	Auckland, Wekarua Is.	34.936 S 173.654 E	10m	Lumpy	Uniporates
Corallinales sp. ZD	Corallinales	NZC2127	MK413619	Auckland, Matauri Bay	35.027 S 173.914 E	-	Encrusting	Uniporates
Corallinales sp. ZD	Corallinales	NZC2130	KM369055	Auckland, Matauri Bay	35.027 S 173.914 E	-	Encrusting	Uniporates
Corallinales sp. ZE	Corallinales	NZC2348	MK413604	Auckland, Pihakoa Point	34.83 S 173.452 E	10m	Epizoic	Uniporates
Corallinales sp. ZE	Corallinales	NZC2348	MK413604	Auckland, Pihakoa Point	34.83 S 173.452 E	10m	Epizoic	Uniporates
Corallinales sp. ZE	Corallinales	NZC2348	MK413604	Auckland, Pihakoa Point	34.83 S 173.452 E	10m	Epizoic	Uniporates
Corallinales sp. ZE	Corallinales	NZC2348	MK413604	Auckland, Pihakoa Point	34.83 S 173.452 E	10m	Epizoic	Uniporates
Corallinales sp. ZE	Corallinales	NZC2348	MK413604	Auckland, Pihakoa Point	34.83 S 173.452 E	10m	Epizoic	Uniporates
Corallinales sp. ZE	Corallinales	NZC2590	FJ361720	Gisborne, Lottin Point	37.553 S 178.164 E	-	Lumpy	Uniporates
Corallinales sp. ZF	Corallinales	NZC0491	DQ167958	Nelson, Mussel Farm Bay	40.807 S 172.929 E	0m	Encrusting	Uniporates
Corallinales sp. ZF	Corallinales	NZC5550	MK413314	Otago, Moeraki	45.362 S 170.863 E	1m	Lumpy	-
Corallinales sp. ZF	Corallinales	NZC5562	MK413304	Otago, Moeraki	45.362 S 170.863 E	1m	Lumpy	Uniporates- Dome
Corallinales sp. ZF	Corallinales	NZC5566	MK413300	Otago, Moeraki	45.362 S 170.863 E	1m	Encrusting	Uniporates- Dome
Corallinales sp. ZF	Corallinales	NZC5571	MK413295	Otago, Moeraki	45.362 S 170.863 E	1m	Encrusting	-
Corallinales sp. ZF	Corallinales	NZC5577	MK413290	Otago, Moeraki	45.362 S 170.863 E	1m	Encrusting	Uniporates- Dome

Corallinales sp. ZF	Corallinales	NZC5593	MK413275	Otago, Moeraki	45.362 S 170.863 E	1m	Lumpy	Uniporates- Dome
Corallinales sp. ZF	Corallinales	NZC5604	MK413264	Otago, Moeraki	45.362 S 170.863 E	1m	Encrusting	-
Corallinales sp. ZF	Corallinales	NZC5608	MK413260	Otago, Moeraki	45.362 S 170.863 E	1m	Lumpy	Uniporates- Dome
Corallinales sp. ZG	Corallinales	NZC2237	FJ361543	Auckland, Moturoa & Motu channel	35.213 S 174.197 E	14m	Epiphytic	Uniporates
Corallinales sp. ZG	Corallinales	NZC2308	FJ361574	Auckland, Urupukapuka	35.205 S 174.234 E	14m	Epilithic	Uniporates
Corallinales sp. ZG	Corallinales	NZC2410	FJ361596	Auckland, Wekarua Is.	34.936 S 173.654 E	10m	Encrusting	Uniporates
Corallinales sp. ZG	Corallinales	NZC5022	MK413580	Otago, Butterfly Bay	45.638 S 170.672 E	1m	Epilithic	Uniporates
Corallinales sp. ZG	Corallinales	NZC5049	MK413568	Southland, River Mouth	46.261 S 167.204 E	0m	Warty	Uniporates
Corallinales sp. ZG	Corallinales	NZC5336	MK413428	Westland, Ocean Beach	43.966 S 168.607 E	0m	Encrusting	-
Corallinales sp. ZG	Corallinales	NZC5542	MK413322	Otago, Moeraki	45.362 S 170.863 E	1m	Warty	Uniporates- Dome
Corallinales sp. ZG	Corallinales	NZC5560	MK413306	Otago, Moeraki	45.362 S 170.863 E	1m	Encrusting	Uniporates- Dome
Corallinales sp. ZG	Corallinales	NZC5563	MK413303	Otago, Moeraki	45.362 S 170.863 E	1m	Encrusting	Uniporates- Dome
Corallinales sp. ZG	Corallinales	NZC5565	MK413301	Otago, Moeraki	45.362 S 170.863 E	1m	Lumpy	Uniporates- Dome
Corallinales sp. ZG	Corallinales	NZC5567	MK413299	Otago, Moeraki	45.362 S 170.863 E	1m	Lumpy	Uniporates- Dome
Corallinales sp. ZG	Corallinales	NZC5578	MK413289	Otago, Moeraki	45.362 S 170.863 E	1m	Lumpy	Uniporates- Dome
Corallinales sp. ZG	Corallinales	NZC5580	MK413287	Otago, Moeraki	45.362 S 170.863 E	1m	Warty	Uniporates- Dome
Corallinales sp. ZG	Corallinales	NZC5582	MK413286	Otago, Moeraki	45.362 S 170.863 E	1m	Encrusting	Uniporates- Flush
Corallinales sp. ZG	Corallinales	NZC5585	MK413283	Otago, Moeraki	45.362 S 170.863 E	1m	Warty	-
Corallinales sp. ZG	Corallinales	NZC5591	MK413277	Otago, Moeraki	45.362 S 170.863 E	1m	Lumpy	Uniporates- Dome
Corallinales sp. ZG	Corallinales	NZC5603	MK413265	Otago, Moeraki	45.362 S 170.863 E	1m	Encrusting	Uniporates- Dome
Corallinales sp. ZG	Corallinales	NZC5605	MK413263	Otago, Moeraki	45.362 S 170.863 E	1m	Lumpy	Uniporates- Dome
Corallinales sp. ZG	Corallinales	NZC5606	MK413262	Otago, Moeraki	45.362 S 170.863 E	1m	Lumpy	Uniporates- Dome
Corallinales sp. ZG	Corallinales	NZC5718	MK413214	Fiordland, North Port	46.035 S 166.592 E	1m	Encrusting	Uniporates- Dome
Jania sagittata	Corallinales	ASD165	EF628226	Gisborne, Tatapouri Boat Ramp	38.648 S 178.146 E	-	-	-
Jania sagittata	Corallinales	NZC2161	FJ361467	Auckland, Kiritehere Beach	38.325 S 174.703 E	-	Geniculate	-
Jania sagittata	Corallinales	NZC2216	FJ361560	Auckland, Rangiatea	35.217 S 174.181 E	13m		-
Jania sagittata	Corallinales	NZC2216	FJ361560	Auckland, Rangiatea	35.217 S 174.181 E	13m		-
Jania sagittata	Corallinales	NZC2225	FJ361484	Auckland, Rangiatea	35.217 S 174.181 E	13m		-
Jania sagittata	Corallinales	NZC2295	FJ361668	Auckland, Urupukapuka	35.205 S 174.234 E	14m	Geniculate	-
Jania sagittata	Corallinales	NZC2325	FJ361571	Auckland, Wekarua Is.	34.936 S 173.654 E	10m	Geniculate	-
Jania sagittata	Corallinales	NZC2339	FJ361578	Auckland, Wekarua Is.	34.936 S 173.654 E	10m	Geniculate	-
Jania sagittata	Corallinales	NZC2364	FJ361600	Auckland, North Cape	34.418 S 173.052 E	8m	Geniculate	-
Jania sagittata	Corallinales	NZC2389	KM369032	Auckland, Motuharakeke	35.003 S 173.97 E	5m	Geniculate	-
Jania sagittata	Corallinales	NZC2532	FJ361674	Auckland, Kapowairua	34.421 S 172.856 E	-	Geniculate	Uniporates

Jania sagittata	Corallinales	NZC2533	FJ361673	Auckland, Kapowairua	34.421 S 172.856 E	-	Geniculate	-
Jania sp. A	Corallinales	NZC5426	MK413379	Stewart Is., Black & White Bay	46.692 S 167.888 E	2m	Geniculate	-
Jania sp. B	Corallinales	NZC2037	FJ361363	Auckland, Rocky Bay	36.819 S 175.053 E	1m	Geniculate	-
Jania sp. B	Corallinales	NZC2049	FJ361547	Auckland, Jones Bay	36.377 S 174.824 E	-	-	-
Jania sp. B	Corallinales	NZC2087A	FJ361457	Auckland, Ocean Beach	35.835 S 174.573 E	-	Geniculate	-
Jania sp. B	Corallinales	NZC2087B	FJ361456	Auckland, Ocean Beach	35.835 S 174.573 E	-	Geniculate	-
Jania sp. B	Corallinales	NZC2492	FJ361741	Auckland, Ahipara	35.175 S 173.117 E	-	Geniculate	Uniporates
Jania sp. B	Corallinales	NZC2538	FJ361677	Auckland, Kapowairua	34.421 S 172.856 E	-	Geniculate	Uniporates
Jania sp. B	Corallinales	NZC2553	FJ361734	Auckland, The Bluff	34.685 S 172.89 E	-	Geniculate	Uniporates
Jania sp. B	Corallinales	NZC2554	KM369034	Auckland, The Bluff	34.685 S 172.89 E	-	Geniculate	Uniporates
Jania sp. B	Corallinales	NZC2568	FJ361737	Auckland, Tapotupotu Bay	34.435 S 172.717 E	-	Geniculate	Uniporates
Jania sp. C	Corallinales	NZC2234	FJ361368	Auckland, Okahu Is.	35.201 S 174.205 E	14m	Geniculate	-
Jania sp. E	Corallinales	NZC5062	MK413560	Southland, Crombie	46.257 S 167.164 E	0m	Geniculate	-
Jania sp. E	Corallinales	NZC5216	MK413508	Otago, Kaka Point, Tawhiri	46.431 S 169.798 E	0m	Geniculate	-
Jania sp. E	Corallinales	NZC5238	MK413493	Southland, Waikawa, Waipapa	46.661 S 168.846 E	0m	Geniculate	-
Jania sp. F	Corallinales	ASE294	EF628228	Stewart Island, Port William	46.845 S 168.083 E	-	-	-
Jania sp. F	Corallinales	ASG243	FJ361569	Chatham Island, Wharekauri	-	-	-	-
Jania sp. F	Corallinales	NZC2022	KM369033	Auckland, Te Henga	36.887 S 174.437 E	-	Geniculate	-
Jania sp. F	Corallinales	NZC2159	FJ361471	Auckland, Kiritehere Beach	38.325 S 174.703 E	-	Geniculate	Uniporates
Jania sp. F	Corallinales	NZC2299	FJ361500	Auckland, Okahu Channel	35.197 S 174.216 E	14m	Geniculate	-
Jania sp. F	Corallinales	NZC2488	FJ361681	Auckland, Ahipara	35.175 S 173.117 E	-	Epiphytic	-
Jania sp. F	Corallinales	NZC5002	MK413588	Otago, Karitane	45.642 S 170.678 E	10m	Geniculate	-
Jania sp. F	Corallinales	NZC5006	MK413585	Otago, Murdering Beach West	45.761 S 170.666 E	0m	Geniculate	-
Jania sp. F	Corallinales	NZC5053B	MK413566	Southland, Crombie	46.257 S 167.164 E	0m	Geniculate	-
Jania sp. F	Corallinales	NZC5127	MK413538	Fiordland, Catseye Bay	44.81 S 167.382 E	0	Geniculate	-
Jania sp. F	Corallinales	NZC5154	MK413526	Fiordland, Anchor Island	45.761 S 166.537 E	5m	Geniculate	Conceptacle
Jania sp. F	Corallinales	NZC5207	MK413510	Fiordland, Sunday Cove	45.594 S 166.741 E	1m	Geniculate	-
Jania sp. F	Corallinales	NZC5218A	MK413506	Otago, Kaka Point, Tawhiri	46.431 S 169.798 E	0m	Geniculate	-
Jania sp. F	Corallinales	NZC5237	MK413494	Southland, Waikawa, Waipapa	46.661 S 168.846 E	0m	Geniculate	-
Jania sp. F	Corallinales	NZC5260	MK413478	Southland, Riverton, Cosy Nook	46.331 S 167.72 E	0m	Geniculate	-
Jania sp. F	Corallinales	NZC5279	MK413467	Otago, Moeraki, Tawhiroko N	45.367 S 170.866 E	0m	Geniculate	-
Jania sp. F	Corallinales	NZC5281	MK413465	Otago, Moeraki, Tawhiroko N	45.37 S 170.867 E	0m	Geniculate	-
Jania sp. F	Corallinales	NZC5301	MK413453	Otago, Moeraki, Paitu Head	45.36 S 170.864 E	3m	Geniculate	-
Jania sp. F	Corallinales	NZC5339	MK413425	Westland, Ocean Beach	43.966 S 168.607 E	0m	Geniculate	-

Jania sp. F	Corallinales	NZC5340	MK413424	Westland, Ocean Beach	43.966 S 168.607 E	0m	Geniculate	Conceptacle
Jania sp. F	Corallinales	NZC5341	MK413423	Westland, Ocean Beach	43.966 S 168.607 E	0m	Geniculate	-
Jania sp. F	Corallinales	NZC5385	MK413402	Stewart Island, Horseshoe Bay	46.878 S 168.148 E	2m	Geniculate	-
Jania sp. F	Corallinales	NZC5435	MK413372	Stewart Is., Black & White Bay	46.692 S 167.888 E	2m	Geniculate	Conceptacle
Jania sp. F	Corallinales	NZC5634	MK413247	Fiordland, Spit Islands	46.074 S 166.633 E	7m	Geniculate	-
Jania sp. J	Corallinales	ASD196	EF628225	Auckland, Te Werahi Beach	34.438 S 172.678 E	-	-	-
Jania sp. J	Corallinales	NZC2020	FJ361388	Auckland, Te Henga	36.887 S 174.437 E	-	Geniculate	-
Jania sp. J	Corallinales	NZC2036	FJ361398	Auckland, Rocky Bay	36.819 S 175.053 E	1m	Geniculate	-
Jania sp. J	Corallinales	NZC2048	FJ361546	Auckland, Jones Bay	36.377 S 174.824 E	-	Geniculate	-
Jania sp. J	Corallinales	NZC2050	FJ361405	Auckland, Jones Bay	36.377 S 174.824 E	-	Geniculate	-
Jania sp. J	Corallinales	NZC2070	FJ361406	Auckland, Lang's Beach	36.043 S 174.532 E	-	Geniculate	-
Jania sp. J	Corallinales	NZC2085	FJ361465	Auckland, Ocean Beach	35.835 S 174.573 E	-	Geniculate	-
Jania sp. J	Corallinales	NZC2149	FJ361562	Taranaki, Waitara	38.988 S 174.219 E	-	Geniculate	-
Jania sp. J	Corallinales	NZC2151	MK413615	Taranaki, Waitara	38.988 S 174.219 E	-	Encrusting	Uniporates
Jania sp. J	Corallinales	NZC2160	FJ361470	Auckland, Kiritehere Beach	38.325 S 174.703 E	-	Geniculate	-
Jania sp. J	Corallinales	NZC2424	FJ361660	Auckland, Sailors Grave	36.961 S 175.844 E	-	Geniculate	-
Jania sp. J	Corallinales	NZC2426	FJ361631	Auckland, Sailors Grave	36.961 S 175.844 E	-	Geniculate	-
Jania sp. J	Corallinales	NZC2449	FJ361656	Auckland, Little Bay	36.601 S 175.549 E	-	Geniculate	-
Jania sp. J	Corallinales	NZC2471	FJ361653	Auckland, Fletcher Bay	36.475 S 175.392 E	-	Geniculate	-
Jania sp. J	Corallinales	NZC2494	FJ361740	Auckland, Ahipara	35.175 S 173.117 E	-	Geniculate	-
Jania sp. J	Corallinales	NZC2513	FJ361756	Auckland, Henderson Point	34.741 S 173.118 E	-	Encrusting	Uniporates
Jania sp. J	Corallinales	NZC2526	FJ361752	Auckland, Henderson Point	34.741 S 173.118 E	-	Geniculate	-
Jania sp. J	Corallinales	NZC2534	FJ361686	Auckland, Kapowairua	34.421 S 172.856 E	-	Geniculate	-
Jania sp. J	Corallinales	NZC2556	FJ361712	Auckland, The Bluff	34.685 S 172.89 E	-	Geniculate	-
Jania sphaeroramosa	Corallinales	ASD163	EF628227	Gisborne, Kaiti Beach	38.684 S 178.032 E	-	-	-
Jania sphaeroramosa	Corallinales	ASE051	DQ167913	Chatham Island, Wharekauri	43.707 S 176.574 E	-	-	-
Jania sphaeroramosa	Corallinales	ASE281	KM369031	Stewart Island, Lee Bay	-	-	-	-
Jania sphaeroramosa	Corallinales	ASG244	FJ361568	Chatham Island, Wharekauri	-	-	-	-
Jania sphaeroramosa	Corallinales	ASN229	MH017054	Auckland Islands, Derry Castle	50.484 S 166.304 E	0m	Geniculate	-
Jania sphaeroramosa	Corallinales	DH4	DQ168020	Otago	-	-	-	-
Jania sphaeroramosa	Corallinales	DH7	DQ168021	Otago	-	-	-	-
Jania sphaeroramosa	Corallinales	NZC5003	MH010584	Otago, Murdering Beach West	45.761 S 170.666 E	0m	Geniculate	Conceptacle
Jania sphaeroramosa	Corallinales	NZC5042	MH010586	Otago, Wellers Rock	45.798 S 170.715 E	0m	Geniculate-Epiphytic	-

Jania sphaeroramosa	Corallinales	NZC5048	MH010585	Southland, Snout	46.26 S 167.194 E	0m	Geniculate-Epiphytic	Conceptacle
Jania sphaeroramosa	Corallinales	NZC5219	MH017058	Otago, Kaka Point, Tawhiri	46.431 S 169.798 E	0m	Geniculate	-
Jania sphaeroramosa	Corallinales	NZC5234	MH010587	Southland, Waikawa, Waipapa	46.661 S 168.846 E	0m	Geniculate-Epiphytic	-
Jania sphaeroramosa	Corallinales	NZC5258	MK413480	Southland, Riverton, Cosy Nook	46.331 S 167.72 E	0m	Geniculate	-
Jania sphaeroramosa	Corallinales	NZC5274	MH017061	Otago, Moeraki, Tikoraki North	45.367 S 170.866 E	0m	Geniculate-Epiphytic	Conceptacle
Jania sphaeroramosa	Corallinales	NZC5358	MH026110	Otago, Chaslands	46.625 S 169.361 E	0m	Geniculate-Epiphytic	Conceptacle
Jania sphaeroramosa	Corallinales	NZC5400	MH026111	Stewart Island, Tikotatahi	47.087 S 168.152 E	0m	Geniculate-Epiphytic	-
Jania sphaeroramosa	Corallinales	NZC5627	MK413251	Fiordland, Spit Islands	46.072 S 166.634 E	0m	Geniculate-Epiphytic	Conceptacle
Jania sphaeroramosa	Corallinales	NZC5666	MK413233	Fiordland, Halt Bay	46.028 S 166.547 E	0m	Geniculate	-
Mastophora pacifica	Corallinales	NZC2000	FJ361365	Kermadec Islands, Denham Bay	29.267 S 177.95 W	13m	Epiphytic	Uniporates
Pneophyllum sp. A	Corallinales	NZC2023	FJ361545	Auckland, Te Henga	36.887 S 174.437 E	-	Epiphytic	Uniporates
Pneophyllum sp. B	Corallinales	NZC0623	DQ167916	Canterbury, Sumner Head	43.571 S 172.772 E	-	Encrusting	Uniporates
Pneophyllum sp. B	Corallinales	NZC5564	MK413302	Otago, Moeraki	45.362 S 170.863 E	1m	Encrusting	Uniporates- Flush
Pneophyllum sp. B	Corallinales	NZC5706B	MK413218	Fiordland, Small Craft Harbour Is.	45.967 S 166.651 E	5m	Encrusting- Epizoic	Uniporates- Flush
Pneophyllum sp. B	Corallinales	NZC5745E	MK413211	Otago, Butterfly Bay	45.638 S 170.672 E	10m	Epilithic	-
Pneophyllum sp. B	Corallinales	NZC5747A	MK413209	Otago, Butterfly Bay	45.638 S 170.672 E	10m	Epilithic	-
Pneophyllum sp. C	Corallinales	ASN195	MK413634	Auckland Islands, Derry Castle	50.484 S 166.304 E	0m	Lumpy- Epiltihic	Uniporates- Flush
Pneophyllum sp. D	Corallinales	NZC0507	EF628233	Nelson, Mussel Farm Bay	40.807 S 172.929 E	-	Epizoic	Uniporates
Pneophyllum sp. E	Corallinales	NZC5232B	MK413496	Southland, Waikawa, Waipapa	46.661 S 168.846 E	0m	Encrusting	Uniporates- Flush
Pneophyllum sp. E	Corallinales	NZC5323	MK413439	Westland, Jackson Bay	43.972 S 168.616 E	0m	Lumpy	Uniporates- Flush
Pneophyllum sp. F	Corallinales	NZC0624	DQ167914	Canterbury, Taylor's Mistake	43.585 S 172.774 E	0m	Epizoic	Uniporates
Pneophyllum sp. F	Corallinales	NZC0627	EF628234	Canterbury, Taylor's Mistake	43.585 S 172.774 E	0m	Epizoic	Uniporates
Pneophyllum sp. F	Corallinales	NZC0645	DQ167901	Canterbury, Taylor's Mistake	43.585 S 172.774 E	5m	Epizoic	Uniporates
Pneophyllum sp. G	Corallinales	NZC0730	KM369048	Chatham Island, Okawa Point	43.77 S 176.247 W	-	Encrusting	Uniporates
Pneophyllum sp. H	Corallinales	NZC5746C	MK413210	Otago, Butterfly Bay	45.638 S 170.672 E	10m	Epilithic	-
Pneophyllum sp. I	Corallinales	NZC2593	FJ361693	Gisborne, Lottin Point	37.553 S 178.164 E	-	Epiphytic	Uniporates
Pneophyllum sp. I	Corallinales	NZC5063	MK413559	Southland, Crombie	46.257 S 167.164 E	0m	Epiphytic	Uniporates- Flush
Pneophyllum sp. J	Corallinales	NZC0686	KM369045	Hawkes Bay, Tuingara Point	40.121 S 176.875 E	-	Encrusting	Multiporates
Pneophyllum sp. J	Corallinales	NZC5720	MK413212	Fiordland, North Port	46.035 S 166.592 E	1m	Encrusting-Epiphytic	Uniporates- Dome
Pneophyllum sp. K	Corallinales	DH5_2	DQ168024	Otago	-	-	-	-
Pneophyllum sp. K	Corallinales	NZC0715	DQ167967	Chatham Island, Te One Creek	44.019 S 176.383 W	0m	Encrusting	Uniporates

Pneophyllum sp. K	Corallinales	NZC0737	DQ167969	Chatham Island, Te One Creek	44.019 S 176.383 W	-	Encrusting	Uniporates
Pneophyllum sp. K	Corallinales	NZC2019	KM369043	Auckland, Te Henga	36.887 S 174.437 E	-	Epiphytic	Uniporates
Pneophyllum sp. K	Corallinales	NZC5285	MK413462	Otago, Moeraki, Okahau South	45.382 S 170.868 E	3m	Epiphytic	Uniporates
Pneophyllum sp. K	Corallinales	NZC5344	MK413421	Otago, Chaslands	46.625 S 169.361 E	2m	Encrusting-Epiphytic	Uniporates- Dome
Pneophyllum sp. K	Corallinales	NZC5431	MK413375	Stewart Is., Black & White Bay	46.692 S 167.888 E	2m	Encrusting-Epiphytic	Uniporates- Dome
Pneophyllum sp. K	Corallinales	NZC5514	MK413335	Otago, Green Island	45.952 S 170.386 E	0m	Encrusting-Epiphytic	Uniporates- Dome
Hapalidiales sp. A	Hapalidiales	NZC2090	KM369026	Auckland, Ocean Beach	35.835 S 174.573 E	-	Encrusting	Multiporates
Hapalidiales sp. A	Hapalidiales	NZC2582	FJ361688	Auckland, Tapotupotu Bay	34.435 S 172.717 E	-	Fruticose	Uniporates
Hapalidiales sp. A	Hapalidiales	NZC5251B	MK413484	Southland, Riverton, Monkey Is.	46.3 S 167.725 E	0m	Epilithic	Multiporates - Flat top
Hapalidiales sp. B	Hapalidiales	NZC5574	MK413293	Otago, Moeraki	45.362 S 170.863 E	1m	Encrusting	Uniporates- Dome
Hapalidiales sp. B	Hapalidiales	NZC5598	MK413270	Otago, Moeraki	45.362 S 170.863 E	1m	Encrusting	-
Hapalidiales sp. B	Hapalidiales	NZC5600	MK413268	Otago, Moeraki	45.362 S 170.863 E	1m	Encrusting	-
Hapalidiales sp. C	Hapalidiales	NZC5470	MK413359	Southland, Bluff, site 24021	46.58 S 168.329 E	6m	Lumpy	Uniporates- Pointy
Hapalidiales sp. C	Hapalidiales	NZC5677	MK413227	Fiordland, Little Island	45.973 S 166.589 E	5m	Fruticose	Multiporates - Flat top
Hapalidiales sp. C	Hapalidiales	NZC5678	MK413226	Fiordland, Little Island	45.973 S 166.589 E	5m	Warty	Uniporates- Pointy
Hapalidiales sp. D	Hapalidiales	NZC0841	DQ167997	Wellington, Makara	41.219 S 174.714 E	10m	Epilithic	Uniporates
Hapalidiales sp. D	Hapalidiales	NZC5396	MK413395	Stewart Island, Passage Rock	47.078 S 168.205 E	2m	Lumpy	-
Hapalidiales sp. D	Hapalidiales	NZC5447	MK413365	Southland, Caroline Bay	46.755 S 168.493 E	2m	Epilithic	Multiporates - Flush
Hapalidiales sp. D	Hapalidiales	NZC5463	MK413362	Southland, Bluff, site 24245	46.576 S 168.312 E	3m	Fruticose	Multiporates - Flush
Hapalidiales sp. D	Hapalidiales	NZC5475	MK413357	Southland, Bluff, site 24028	46.583 S 168.329 E	6m	Epilithic	Multiporates - Flat top
Hapalidiales sp. D	Hapalidiales	NZC5556	MK413309	Otago, Moeraki	45.362 S 170.863 E	1m	Encrusting	-
Hapalidiales sp. D	Hapalidiales	NZC5589	MK413279	Otago, Moeraki	45.362 S 170.863 E	1m	Encrusting	-
Hapalidiales sp. D	Hapalidiales	NZC5601	MK413267	Otago, Moeraki	45.362 S 170.863 E	1m	Encrusting	-
Hapalidiales sp. D	Hapalidiales	NZC5658A	MK413238	Fiordland, Straggle Head	46.032 S 166.546 E	1m	Lumpy	Multiporates - Flat top
Hapalidiales sp. D	Hapalidiales	NZC5681	MK413225	Fiordland, Little Island	45.973 S 166.589 E	5m	Lumpy	Multiporates - Flat top
Hapalidiales sp. D	Hapalidiales	NZC5713	MK413216	Fiordland, North Port	46.035 S 166.592 E	1m	Fruticose	Multiporates - Flat top
Hapalidiales sp. D	Hapalidiales	NZC5717	MK413215	Fiordland, North Port	46.035 S 166.592 E	1m	Lumpy	-
Hapalidiales sp. E	Hapalidiales	ASE278	DQ168018	Stewart Island, Lee Bay	-	-	-	-
Hapalidiales sp. E	Hapalidiales	NZC0092	EF628213	Marlborough, Rakautara BBQ	42.27 S 173.8 E	-	Lumpy	Multiporates
Hapalidiales sp. E	Hapalidiales	NZC0734	DQ167923	Chatham Island, Te One Creek	44.019 S 176.383 W	-	Encrusting	Uniporates
Hapalidiales sp. E	Hapalidiales	NZC0743	DQ167927	Chatham Island, Point Durham	44 S 176.675 W	-	Encrusting	-
Hapalidiales sp. E	Hapalidiales	NZC0756	DQ168013	Chatham Island, Whangatete Inlet	43.798 S 176.684 W	-	Encrusting	Multiporates
Hapalidiales sp. E	Hapalidiales	NZC0763	DQ167964	Chatham Island, Wharekauri	43.707 S 176.574 W	-	Encrusting	Multiporates

Hapalidiales sp. E	Hapalidiales	NZC0764	DQ167981	Chatham Island, Wharekauri	43.707 S 176.574 W	-	Encrusting	Multiporates
Hapalidiales sp. E	Hapalidiales	NZC0768	DQ167965	Chatham Island, Okawa Point	43.77 S 176.247 W	-	Lumpy	Multiporates
Hapalidiales sp. E	Hapalidiales	NZC0773	DQ167948	Chatham Island, Heaphy Shoal	43.966 S 176.593 W	-	Lumpy	-
Hapalidiales sp. E	Hapalidiales	NZC0780	DQ167947	Chatham Island, Tommy Solomon Monument	44.032 S 176.337 W	0m	Encrusting	Multiporates
Hapalidiales sp. E	Hapalidiales	NZC0782	DQ167959	Chatham Island, Te One Creek	44.019 S 176.383 W	-	Encrusting	Multiporates
Hapalidiales sp. E	Hapalidiales	NZC0784	DQ167943	Chatham Island, Te One Creek	44.019 S 176.383 W	-	Epilithic	Multiporates
Hapalidiales sp. E	Hapalidiales	NZC0786	DQ167942	Chatham Island, Te One Creek	44.019 S 176.383 W	-	Encrusting	Multiporates
Hapalidiales sp. E	Hapalidiales	NZC5011	MK413584	Otago, Murdering Beach West	45.761 S 170.666 E	0m	Epilithic	Multiporates - Flat top
Hapalidiales sp. E	Hapalidiales	NZC5066	MK413558	Southland, Snout	46.26 S 167.194 E	0m	Warty	-
Hapalidiales sp. E	Hapalidiales	NZC5224	MK413501	Otago, Kaka Point, Tawhiri	46.428 S 169.796 E	0m	Warty	Multiporates - Flat top
Hapalidiales sp. E	Hapalidiales	NZC5228	MK413499	Otago, Kaka Point, Short Bay	46.408 S 169.797 E	0m	Epilithic	-
Hapalidiales sp. E	Hapalidiales	NZC5230	MK413498	Southland, Waikawa, Waipapa	46.661 S 168.846 E	0m	Epilithic	Multiporates - Flush
Hapalidiales sp. E	Hapalidiales	NZC5247	MK413487	Southland, Riverton, Monkey Is.	46.3 S 167.725 E	0m	Epilithic	Multiporates - Flat top
Hapalidiales sp. E	Hapalidiales	NZC5254	MK413482	Southland, Riverton, Cosy Nook	46.331 S 167.72 E	0m	Epilithic	Multiporates - Flat top
Hapalidiales sp. E	Hapalidiales	NZC5270	MK413471	Otago, Butterfly Bay	45.638 S 170.672 E	1m	Epilithic	Multiporates - Flat top
Hapalidiales sp. E	Hapalidiales	NZC5318	MK413443	Westland, Jackson Bay	43.972 S 168.616 E	0m	Encrusting	-
Hapalidiales sp. E	Hapalidiales	NZC5409	MK413390	Stewart Island, Tikotatahi	47.087 S 168.152 E	0m	Epilithic	-
Hapalidiales sp. E	Hapalidiales	NZC5483	MK413354	Otago, Dundein, Akatore	46.112 S 170.192 E	0m	Encrusting	Uniporates- Dome
Hapalidiales sp. E	Hapalidiales	NZC5568	MK413298	Otago, Moeraki	45.362 S 170.863 E	1m	Encrusting	-
Hapalidiales sp. E	Hapalidiales	NZC5569	MK413297	Otago, Moeraki	45.362 S 170.863 E	1m	Lumpy	-
Hapalidiales sp. E	Hapalidiales	NZC5576	MK413291	Otago, Moeraki	45.362 S 170.863 E	1m	Encrusting	-
Hapalidiales sp. F	Hapalidiales	NZC2238	FJ361372	Auckland, Okahu Is.	35.201 S 174.205 E	14m	Epizoic	Uniporates
Hapalidiales sp. G	Hapalidiales	NZC5173	MK413518	Fiordland, Gaer Arm	45.303 S 167.158 E	14m	Discoid- Epiphytic	-
Hapalidiales sp. G	Hapalidiales	NZC5623	MK413253	Fiordland, Cavern Head	46.081 S 166.648 E	3m	Warty- Epizoic	Multiporates - Flat top
Hapalidiales sp. H	Hapalidiales	NZC5160	MK413522	Fiordland, Five Fingers Pen.	45.712 S 166.5 E	6m	Encusting	Uniporates- Dome
Hapalidiales sp. H	Hapalidiales	NZC5294	MK413456	Otago, Moeraki, Katiki Point E	45.397 S 170.869 E	0m	Encrusting	Multiporates - Flat top
Hapalidiales sp. H	Hapalidiales	NZC5498	MK413345	Otago, Green Island	45.952 S 170.386 E	0m	Encrusting	Multiporates - Flat top
Hapalidiales sp. H	Hapalidiales	NZC5544	MK413320	Otago, Moeraki	45.362 S 170.863 E	1m	Lumpy	-
Hapalidiales sp. H	Hapalidiales	NZC5595	MK413273	Otago, Moeraki	45.362 S 170.863 E	1m	Warty	-
Hapalidiales sp. I	Hapalidiales	NZC0818	DQ167972	Hawkes Bay, Tuingara Point	40.121 S 176.875 E	15m	Encrusting	Multiporates
Hapalidiales sp. I	Hapalidiales	NZC2013	KM369024	Auckland, Te Henga	36.887 S 174.437 E	-	Encrusting	Multiporates
Hapalidiales sp. I	Hapalidiales	NZC2016	FJ361384	Auckland, Te Henga	36.887 S 174.437 E	-	Encrusting	Multiporates
Hapalidiales sp. I	Hapalidiales	NZC2045	FJ361364	Auckland, Jones Bay	36.377 S 174.824 E	-	Epilithic	Multiporates
Hapalidiales sp. I	Hapalidiales	NZC2165	FJ361366	Auckland, Kiritehere Beach	38.325 S 174.703 E	-	Warty	Multiporates

Hapalidiales sp. I	Hapalidiales	NZC2610	FJ361723	Gisborne, Lottin Point	37.553 S 178.164 E	-	Lumpy	Multiporates
Hapalidiales sp. I	Hapalidiales	NZC5023	MK413579	Otago, Butterfly Bay	45.638 S 170.672 E	1m	Epilithic	Multiporates - Volcano
Hapalidiales sp. I	Hapalidiales	NZC5549	MK413315	Otago, Moeraki	45.362 S 170.863 E	1m	Lumpy	Multiporates - Flat top
Hapalidiales sp. I	Hapalidiales	NZC5559	MK413307	Otago, Moeraki	45.362 S 170.863 E	1m	Lumpy	Multiporates - Flat top
Hapalidiales sp. I	Hapalidiales	NZC5584	MK413284	Otago, Moeraki	45.362 S 170.863 E	1m	Warty	-
Hapalidiales sp. I	Hapalidiales	NZC5597	MK413271	Otago, Moeraki	45.362 S 170.863 E	1m	Lumpy	Multiporates - Flat top
Hapalidiales sp. J	Hapalidiales	NZC2041	MK413625	Auckland, Rocky Bay	36.819 S 175.053 E	-	Encrusting	-
Hapalidiales sp. K	Hapalidiales	NZC5440	MK413369	Stewart Is., Black & White Bay	46.692 S 167.888 E	2m	Epiltihic	Multiporates - Flat top
Hapalidiales sp. L	Hapalidiales	NZC0847	DQ167993	Wellington, Makara	41.219 S 174.714 E	10m	Epizoic	Uniporates
Hapalidiales sp. M	Hapalidiales	NZC2369	KM369074	Auckland, North Cape	34.418 S 173.052 E	8m	Epizoic	Multiporates
Hapalidiales sp. M	Hapalidiales	NZC2439-1	FJ361629	Auckland, Little Bay	36.601 S 175.549 E	-	Epiphytic	Multiporates
Hapalidiales sp. N	Hapalidiales	NZC5345	MK413420	Otago, Chaslands	46.625 S 169.361 E	2m	Epiphytic	Multiporates - Volcano
Hapalidiales sp. N	Hapalidiales	NZC5526	MK413333	Otago, Dunedin, Gull Rocks	45.905 S 170.651 E	6m	Discoid- Epiphytic	Uniporates- Dome
Hapalidiales sp. O	Hapalidiales	NZC0823	DQ167976	Wellington, Makara	41.219 S 174.714 E	10m	Encrusting	Multiporates
Hapalidiales sp. O	Hapalidiales	NZC0842	DQ167994	Wellington, Makara	41.219 S 174.714 E	10m	Encrusting	Uniporates
Hapalidiales sp. O	Hapalidiales	NZC2204	FJ361511	Auckland, Bland Bay	35.346 S 174.367 E	-	Encrusting	Multiporates
Hapalidiales sp. O	Hapalidiales	NZC2236	KM369018	Auckland, Moturoa & Motu channel	35.213 S 174.197 E	14m	Epiphytic	Uniporates
Hapalidiales sp. O	Hapalidiales	NZC2248	FJ361480	Auckland, Rangiatea	35.217 S 174.181 E	13m	Encrusting	Multiporates
Hapalidiales sp. O	Hapalidiales	NZC2280	FJ361556	Auckland, Okahu Channel	35.197 S 174.216 E	14m	Encrusting	Uniporates
Hapalidiales sp. O	Hapalidiales	NZC2281	FJ361505	Auckland, Okahu Channel	35.197 S 174.216 E	14m	Encrusting	Uniporates
Hapalidiales sp. O	Hapalidiales	NZC2355	FJ361611	Auckland, Pihakoa Point	34.83 S 173.452 E	10m	Encrusting	Multiporates
Hapalidiales sp. O	Hapalidiales	NZC2363	FJ361614	Auckland, North Cape	34.418 S 173.052 E	8m	Encrusting	-
Hapalidiales sp. O	Hapalidiales	NZC2378	FJ361622	Auckland, Staffa Rock	34.788 S 173.324 E	22m	Epiphytic	Uniporates
Hapalidiales sp. O	Hapalidiales	NZC2406	FJ361608	Auckland, Plate Is.	37.659 S 178.562 E	6m	Epiphytic	Uniporates
Hapalidiales sp. O	Hapalidiales	NZC2484	FJ361620	Auckland, Staffa Rock	34.788 S 173.324 E	22m	Epiphytic	Multiporates
Hapalidiales sp. O	Hapalidiales	NZC2542-1	FJ361671	Auckland, Kapowairua	34.421 S 172.856 E	-	Warty	Uniporates
Hapalidiales sp. O	Hapalidiales	NZC2542-2	FJ361672	Auckland, Kapowairua	34.421 S 172.856 E	-	Warty	Uniporates
Hapalidiales sp. O	Hapalidiales	NZC2544	FJ361676	Auckland, Kapowairua	34.421 S 172.856 E	-	Warty	Uniporates
Hapalidiales sp. O	Hapalidiales	NZC2581-2	FJ361700	Auckland, Tapotupotu Bay	34.435 S 172.717 E	-	Epilithic	Uniporates
Hapalidiales sp. P	Hapalidiales	ASE300	EF628217	Stewart Island, Port William	46.845 S 168.083 E	-	-	-
Hapalidiales sp. P	Hapalidiales	NZC5170	MK413519	Fiordland, Five Fingers Pen.	45.712 S 166.5 E	6m	Epilithic	Multiporates - Volcano

Hapalidiales sp. P	Hapalidiales	NZC5362A	MK413413	Stewart Island, Horseshoe Bay	46.878 S 168.148 E	2m	Discoid- Epizoic	Multiporates - Volcano
Hapalidiales sp. P	Hapalidiales	NZC5499	MK413344	Otago, Green Island	45.952 S 170.386 E	0m	Encrusting	Uniporates- Dome
Hapalidiales sp. P	Hapalidiales	NZC5506	MK413339	Otago, Green Island	45.952 S 170.386 E	0m	Encrusting	Uniporates- Pointy
Hapalidiales sp. P	Hapalidiales	NZC5527	MK413332	Otago, Dunedin, Gull Rocks	45.905 S 170.651 E	6m	Encrusting	Multiporates - Volcano
Hapalidiales sp. P	Hapalidiales	NZC5615	MK413256	Fiordland, Cavern Head	46.081 S 166.648 E	3m	Discoid	Multiporates - Volcano
Hapalidiales sp. P	Hapalidiales	NZC5622A	MK413254	Fiordland, Cavern Head	46.081 S 166.648 E	3m	Discoid- Epizoic	Multiporates - Volcano
Hapalidiales sp. Q	Hapalidiales	NZC5352	MK413417	Otago, Chaslands	46.625 S 169.361 E	2m	Warty	Uniporates- Dome
Hapalidiales sp. Q	Hapalidiales	NZC5379	MK413404	Stewart Island, Horseshoe Bay	46.878 S 168.148 E	2m	Discoid-Struts	Multiporates - Volcano
Hapalidiales sp. Q	Hapalidiales	NZC5380	MK413403	Stewart Island, Horseshoe Bay	46.878 S 168.148 E	2m	Discoid-Struts	Uniporates
Hapalidiales sp. Q	Hapalidiales	NZC5414B	MK413387	Stewart Island, Tikotatahi	47.087 S 168.152 E	2m	Warty- Struts	Multiporates - Volcano
Hapalidiales sp. Q	Hapalidiales	NZC5749A	MK413205	Otago, Butterfly Bay	45.638 S 170.672 E	10m	Epilithic	-
Hapalidiales sp. R	Hapalidiales	NZC5097	MK413543	Southland, Foveaux strait	46.76 S 168.273 E	0	Encusting	-
Hapalidiales sp. R	Hapalidiales	NZC5116	MK413541	Southland, Foveaux strait	46.755 S 168.219 E	0	Encusting	-
Hapalidiales sp. R	Hapalidiales	NZC5266	MK413474	Otago, Butterfly Bay	45.638 S 170.672 E	1m	Warty	Multiporates - Volcano
Hapalidiales sp. R	Hapalidiales	NZC5306	MK413451	Otago, Moeraki, Paitu Head	45.36 S 170.864 E	3m	Epilithic	Multiporates - Volcano
Hapalidiales sp. R	Hapalidiales	NZC5614	MK413257	Fiordland, Cavern Head	46.081 S 166.648 E	3m	Discoid	Uniporates- Dome
Hapalidiales sp. R	Hapalidiales	NZC5624	MK413252	Fiordland, Cavern Head	46.081 S 166.648 E	3m	Encrusting	Multiporates - Volcano
Hapalidiales sp. R	Hapalidiales	NZC5695	MK413222	Fiordland, Small Craft Harbour Is.	45.967 S 166.651 E	5m	Lumpy	Uniporates- Dome
Hapalidiales sp. S	Hapalidiales	CUK17874	MK702009	Stewart Island, Horseshoe Bay	46.878 S 168.148 E	0m	-	-
Hapalidiales sp. T	Hapalidiales	NZC5406	MK413391	Stewart Island, Tikotatahi	47.087 S 168.152 E	0m	Epiltihic	Multiporates - Volcano
Hapalidiales sp. U	Hapalidiales	NZC0095	DQ167877	Marlborough, Halfmoon Bay	42.267 S 173.8 E	0m	Warty	Multiporates
Hapalidiales sp. U	Hapalidiales	NZC5290B	MK413458	Otago, Moeraki, Katiki Point E	45.397 S 170.869 E	0m	Epiltihic	Multiporates - Volcano
Hapalidiales sp. U	Hapalidiales	NZC5602	MK413266	Otago, Moeraki	45.362 S 170.863 E	1m	Warty	Multiporates - Volcano
Hapalidiales sp. V	Hapalidiales	NZC0711	EF628218	Chatham Island, Okawa Point	43.77 S 176.247 W	0m	Epizoic	Uniporates
Hapalidiales sp. V	Hapalidiales	NZC2047	FJ361402	Auckland, Jones Bay	36.377 S 174.824 E	-	Encrusting	Uniporates
Hapalidiales sp. V	Hapalidiales	NZC2057	FJ361550	Auckland, Jones Bay	36.377 S 174.824 E	-	Encrusting	Multiporates- Large
Hapalidiales sp. V	Hapalidiales	NZC2134	FJ361453	Auckland, Matauri Bay	35.027 S 173.914 E	-	Lumpy	Multiporates- Volcano

Hapalidiales sp. V	Hapalidiales	NZC2139	FJ361455	Auckland, Cable Bay	34.99 S 173.487 E	-	Encrusting	Uniporates
Hapalidiales sp. V	Hapalidiales	NZC2211	FJ361489	Auckland, Rangiatea	35.217 S 174.181 E	13m	Epiphytic	Uniporates
Hapalidiales sp. V	Hapalidiales	NZC2261	MK413612	Auckland, Moturoa & Motu channel	35.213 S 174.197 E	14m	Encrusting	Uniporates
Hapalidiales sp. V	Hapalidiales	NZC2271	FJ361493	Auckland, Okahu Is.	35.201 S 174.205 E	14m	Epizoic	Multiporates
Hapalidiales sp. V	Hapalidiales	NZC2286	FJ361513	Auckland, Okahu Channel	35.197 S 174.216 E	14m	Encrusting	Uniporates
Hapalidiales sp. V	Hapalidiales	NZC2288	KM369078	Auckland, Okahu Channel	35.197 S 174.216 E	14m	Encrusting	Multiporates
Hapalidiales sp. V	Hapalidiales	NZC2311	MK413610	Auckland, Urupukapuka	35.205 S 174.234 E	14m	Encrusting	Multiporates
Hapalidiales sp. V	Hapalidiales	NZC2336	FJ361634	Auckland, Wekarua Is.	34.936 S 173.654 E	10m	Encrusting	Uniporates
Hapalidiales sp. V	Hapalidiales	NZC2336	FJ361634	Auckland, Wekarua Is.	34.936 S 173.654 E	10m	Encrusting	Uniporates
Hapalidiales sp. V	Hapalidiales	NZC2346	FJ361613	Auckland, Pihakoa Point	34.83 S 173.452 E	10m	Epizoic	Uniporates
Hapalidiales sp. V	Hapalidiales	NZC2368	FJ361616	Auckland, North Cape	34.418 S 173.052 E	8m	Encrusting	Multiporates
Hapalidiales sp. V	Hapalidiales	NZC2373	FJ361609	Auckland, North Cape	34.418 S 173.052 E	8m	Encrusting	Multiporates
Hapalidiales sp. V	Hapalidiales	NZC2486	FJ361606	Auckland, Pihakoa Point	34.83 S 173.452 E	10m	Epizoic	Multiporates
Hapalidiales sp. V	Hapalidiales	NZC2600	FJ361725	Gisborne, Lottin Point	37.553 S 178.164 E	-	Encrusting	Multiporates-Volcano
Hapalidiales sp. V	Hapalidiales	NZC2609	FJ361722	Gisborne, Lottin Point	37.553 S 178.164 E	-	Encrusting	Multiporates-Volcano
Hapalidiales sp. V	Hapalidiales	NZC2612	FJ361692	Gisborne, Lottin Point	37.553 S 178.164 E	-	Lumpy	Uniporates
Hapalidiales sp. W	Hapalidiales	NZC0875	DQ167990	Wellington, Kapiti Is.	40.87 S 174.921 E	16m	Encrusting	Multiporates
Hapalidiales sp. X	Hapalidiales	NZC4001b	MK413594	Antipodes Islands, Archway Is.	-	-	Warty	Multiporates - Flat top
Hapalidiales sp. X	Hapalidiales	NZC4004	MK413592	Bounty Islands, Ruatara Island	-	0	Warty	Multiporates - Flat top
Hapalidiales sp. Y	Hapalidiales	ASE297	KM369017	Stewart Island, Port William	46.845 S 168.083 E	-	-	-
Hapalidiales sp. Y	Hapalidiales	NZC5202	MK413514	Fiordland, Sunday Cove	45.594 S 166.741 E	1m	Discoid- Epizoic	Multiporates - Flat top
Hapalidiales sp. Y	Hapalidiales	NZC5300	MK413454	Otago, Moeraki, Paitu Head	45.36 S 170.864 E	3m	Discoid- Struts	Multiporates - Volcano
Hapalidiales sp. Y	Hapalidiales	NZC5389A	MK413399	Stewart Island, Passage Rock	47.078 S 168.205 E	2m	Epilithic	Multiporates - Volcano
Hapalidiales sp. Y	Hapalidiales	NZC5391	MK413398	Stewart Island, Passage Rock	47.078 S 168.205 E	2m	Epilithic	Multiporates - Volcano
Hapalidiales sp. Y	Hapalidiales	NZC5398	MK413393	Stewart Island, Port Adventure	47.061 S 168.17 E	7m	Encrusting- Struts	-
Hapalidiales sp. Y	Hapalidiales	NZC5414A	MK413388	Stewart Island, Tikotatahi	47.087 S 168.152 E	2m	Encrusting- Struts	-
Hapalidiales sp. Y	Hapalidiales	NZC5414D	MK413385	Stewart Island, Tikotatahi	47.087 S 168.152 E	2m	Discoid- Struts	Uniporates- Pointy
Hapalidiales sp. Y	Hapalidiales	NZC5432	MK413374	Stewart Is., Black & White Bay	46.692 S 167.888 E	2m	Discoid- Struts	Multiporates - Volcano
Hapalidiales sp. Y	Hapalidiales	NZC5497	MK413346	Otago, Green Island	45.952 S 170.386 E	0m	Discoid- Struts	Uniporates- Pointy
Hapalidiales sp. Y	Hapalidiales	NZC5639	MK413244	Fiordland, Spit Islands	46.074 S 166.633 E	7m	Discoid- Struts	Multiporates - Volcano
Hapalidiales sp. Y	Hapalidiales	NZC5663	MK413234	Fiordland, Straggle Head	46.032 S 166.546 E	1m	Discoid- Struts	Uniporates- Pointy

Hapalidiales sp. YA	Hapalidiales	NZC0747	KM369025	Chatham Island, Whangatete Inlet	43.798 S 176.684 W	-	Encrusting	Multiporates
Hapalidiales sp. YA	Hapalidiales	NZC5036	MK413573	Otago, Butterfly Bay	45.639 S 170.672 E	0m	Epilithic	Multiporates - Flat top
Hapalidiales sp. YA	Hapalidiales	NZC5073	MK413555	Southland, Snout	46.26 S 167.194 E	0m	Epilithic	Uniporates
Hapalidiales sp. YA	Hapalidiales	NZC5269	MK413472	Otago, Butterfly Bay	45.638 S 170.672 E	1m	Warty	Multiporates - Flat top
Hapalidiales sp. YA	Hapalidiales	NZC5316	MK413445	Westland, Jackson Bay	43.972 S 168.616 E	0m	Encrusting	Multiporates - Flat top
Hapalidiales sp. YA	Hapalidiales	NZC5322	MK413440	Westland, Jackson Bay	43.972 S 168.616 E	0m	Lumpy	-
Hapalidiales sp. YA	Hapalidiales	NZC5335	MK413429	Westland, Ocean Beach	43.966 S 168.607 E	0m	Warty	-
Hapalidiales sp. YA	Hapalidiales	NZC5446	MK413366	Stewart Island, White Rock E	46.686 S 167.878 E	5m	Encrusting	Multiporates - Flat top
Hapalidiales sp. YA	Hapalidiales	NZC5540	MK413324	Otago, Moeraki	45.362 S 170.863 E	1m	Lumpy	Multiporates - Volcano
Hapalidiales sp. YA	Hapalidiales	NZC5541	MK413323	Otago, Moeraki	45.362 S 170.863 E	1m	Lumpy	Uniporates- Dome
Hapalidiales sp. YA	Hapalidiales	NZC5543	MK413321	Otago, Moeraki	45.362 S 170.863 E	1m	Lumpy	-
Hapalidiales sp. YA	Hapalidiales	NZC5545	MK413319	Otago, Moeraki	45.362 S 170.863 E	1m	Warty	Uniporates- Dome
Hapalidiales sp. YA	Hapalidiales	NZC5547	MK413317	Otago, Moeraki	45.362 S 170.863 E	1m	Lumpy	-
Hapalidiales sp. YA	Hapalidiales	NZC5561	MK413305	Otago, Moeraki	45.362 S 170.863 E	1m	Encrusting	-
Hapalidiales sp. YA	Hapalidiales	NZC5570	MK413296	Otago, Moeraki	45.362 S 170.863 E	1m	Warty	Uniporates- Dome
Hapalidiales sp. YA	Hapalidiales	NZC5573	MK413294	Otago, Moeraki	45.362 S 170.863 E	1m	Epilithic	-
Hapalidiales sp. YA	Hapalidiales	NZC5575	MK413292	Otago, Moeraki	45.362 S 170.863 E	1m	Warty	-
Hapalidiales sp. YA	Hapalidiales	NZC5579	MK413288	Otago, Moeraki	45.362 S 170.863 E	1m	Lumpy	-
Hapalidiales sp. YA	Hapalidiales	NZC5586	MK413282	Otago, Moeraki	45.362 S 170.863 E	1m	Warty	-
Hapalidiales sp. YA	Hapalidiales	NZC5587	MK413281	Otago, Moeraki	45.362 S 170.863 E	1m	Warty	Uniporates- Dome
Hapalidiales sp. YA	Hapalidiales	NZC5588	MK413280	Otago, Moeraki	45.362 S 170.863 E	1m	Warty	-
Hapalidiales sp. YA	Hapalidiales	NZC5590	MK413278	Otago, Moeraki	45.362 S 170.863 E	1m	Warty	-
Hapalidiales sp. YA	Hapalidiales	NZC5592	MK413276	Otago, Moeraki	45.362 S 170.863 E	1m	Lumpy	-
Hapalidiales sp. YA	Hapalidiales	NZC5594	MK413274	Otago, Moeraki	45.362 S 170.863 E	1m	Lumpy	-
Hapalidiales sp. YA	Hapalidiales	NZC5599	MK413269	Otago, Moeraki	45.362 S 170.863 E	1m	Warty	-
Hapalidiales sp. YA	Hapalidiales	NZC5607	MK413261	Otago, Moeraki	45.362 S 170.863 E	1m	Lumpy	Uniporates- Dome
Hapalidiales sp. YA	Hapalidiales	NZC5610	MK413258	Fiordland, Cavern Head	46.081 S 166.648 E	3m	Lumpy	Multiporates - Flat top
Hapalidiales sp. YA	Hapalidiales	NZC5747B	MK413208	Otago, Butterfly Bay	45.638 S 170.672 E	10m	Epilithic	-
Hapalidiales sp. YA	Hapalidiales	NZC5748C	MK413207	Otago, Butterfly Bay	45.638 S 170.672 E	10m	Epilithic	-
Hapalidiales sp. YA	Hapalidiales	NZC5748D	MK413206	Otago, Butterfly Bay	45.638 S 170.672 E	10m	Epilithic	-
Hapalidiales sp. YA	Hapalidiales	NZC5749B	MK413204	Otago, Butterfly Bay	45.638 S 170.672 E	10m	Epilithic	-
Hapalidiales sp. YB	Hapalidiales	NZC0709	KM369014	Chatham Island, Port Hutt	43.816 S 176.705 W	-	Epizoic	Multiporates
Hapalidiales sp. YB	Hapalidiales	NZC0745	EF628222	Chatham Island, Point Durham	44 S 176.675 W	-	Layered	Multiporates
Hapalidiales sp. YB	Hapalidiales	NZC0750	DQ167932	Chatham Island, Port Hutt	43.816 S 176.705 W	-	Encrusting	Multiporates

Hapalidiales sp. YC	Hapalidiales	ASN188C	MK413635	Auckland Islands, Derry Castle	50.484 S 166.304 E	0m	Discoid- Epiphytic	Multiporates - Flat top
Hapalidiales sp. YC	Hapalidiales	ASN254	MK413631	Auckland Islands, Sandy Bay	50.5 S 166.284 E	0m	Discoid	Multiporates - Flat top
Hapalidiales sp. YC	Hapalidiales	NZC0225	EF628223	Wellington, Is. Bay	41.35 S 174.767 E	0m	Discoid	Uniporates
Hapalidiales sp. YC	Hapalidiales	NZC0898	DQ167985	Wellington, Is. Bay	41.35 S 174.767 E	0m	Discoid	Uniporates
Hapalidiales sp. YC	Hapalidiales	NZC5000	MK413590	Otago, Karitane	45.642 S 170.678 E	10m	Discoid- Epiphytic	Multiporates - Flat top
Hapalidiales sp. YC	Hapalidiales	NZC5028	MK413577	Otago, Butterfly Bay	45.638 S 170.672 E	1m	Discoid- Epiphytic	Multiporates - Flat top
Hapalidiales sp. YC	Hapalidiales	NZC5156B	MK413524	Fiordland, Turn Round Point	44.8 S 167.544 E	5m	Discoid- Epiphytic	Multiporates - Flat top
Hapalidiales sp. YC	Hapalidiales	NZC5289B	MK413460	Otago, Moeraki, Katiki Point E	45.397 S 170.869 E	0m	Discoid- Epiphytic	Multiporates - Flat top
Hapalidiales sp. YC	Hapalidiales	NZC5309B	MK413448	Otago, Moeraki, Katiki Point W	45.397 S 170.868 E	3m	Discoid- Epiphytic	Multiporates - Flat top
Hapalidiales sp. YC	Hapalidiales	NZC5329	MK413435	Westland, Jackson Bay	43.972 S 168.616 E	0m	Epiphytic	Uniporates- Dome
Hapalidiales sp. YC	Hapalidiales	NZC5661	MK413236	Fiordland, Straggle Head	46.032 S 166.546 E	1m	Discoid- Epiphytic	Multiporates - Flat top
Hapalidiales sp. YD	Hapalidiales	NZC0728	DQ167921	Chatham Island, Te One Creek	44.019 S 176.383 W	0m	Epizoic	Multiporates
Hapalidiales sp. YD	Hapalidiales	NZC5263	MK413475	Otago, Butterfly Bay	45.638 S 170.672 E	1m	Encrusting	-
Hapalidiales sp. YD	Hapalidiales	NZC5277	MK413468	Otago, Moeraki, Tikoraki North	45.367 S 170.866 E	0m	Epilithic	-
Hapalidiales sp. YD	Hapalidiales	NZC5290A	MK413459	Otago, Moeraki, Katiki Point E	45.397 S 170.869 E	0m	Warty	Multiporates - Volcano
Hapalidiales sp. YD	Hapalidiales	NZC5292	MK413457	Otago, Moeraki, Katiki Point E	45.397 S 170.869 E	0m	Warty	Multiporates - Flat top
Hapalidiales sp. YD	Hapalidiales	NZC5371	MK413408	Stewart Island, Horseshoe Bay	46.878 S 168.148 E	2m	Lumpy	Multiporates - Flush
Hapalidiales sp. YD	Hapalidiales	NZC5387	MK413401	Stewart Island, Passage Rock	47.078 S 168.205 E	2m	Encrusting- Struts	Multiporates - Flush
Hapalidiales sp. YD	Hapalidiales	NZC5388	MK413400	Stewart Island, Passage Rock	47.078 S 168.205 E	2m	Fruticose	Multiporates - Volcano
Hapalidiales sp. YD	Hapalidiales	NZC5414C	MK413386	Stewart Island, Tikotatahi	47.087 S 168.152 E	2m	Discoid- Struts	-
Hapalidiales sp. YD	Hapalidiales	NZC5439	MK413370	Stewart Is., Black & White Bay	46.692 S 167.888 E	2m	Encrusting	Multiporates - Volcano
Hapalidiales sp. YD	Hapalidiales	NZC5525	MK413334	Otago, Dunedin, Gull Rocks	45.905 S 170.651 E	6m	Lumpy- Epizoic	Multiporates - Volcano
Hapalidiales sp. YD	Hapalidiales	NZC5552	MK413313	Otago, Moeraki	45.362 S 170.863 E	1m	Encrusting	-
Hapalidiales sp. YD	Hapalidiales	NZC5553	MK413312	Otago, Moeraki	45.362 S 170.863 E	1m	Lumpy	Multiporates - Flush
Hapalidiales sp. YD	Hapalidiales	NZC5554	MK413311	Otago, Moeraki	45.362 S 170.863 E	1m	Encrusting	-
Hapalidiales sp. YD	Hapalidiales	NZC5555	MK413310	Otago, Moeraki	45.362 S 170.863 E	1m	Encrusting	-
Hapalidiales sp. YD	Hapalidiales	NZC5636	MK413245	Fiordland, Spit Islands	46.074 S 166.633 E	7m	Lumpy	Multiporates - Flat top
Hapalidiales sp. YD	Hapalidiales	NZC5662	MK413235	Fiordland, Straggle Head	46.032 S 166.546 E	1m	Lumpy	Multiporates - Volcano
Hapalidiales sp. YD	Hapalidiales	NZC5676	MK413228	Fiordland, Halt Bay	46.028 S 166.547 E	0m	Encrusting	Uniporates- Dome
Hapalidiales sp. YE	Hapalidiales	NZC2347	FJ361625	Auckland, Pihakoa Point	34.83 S 173.452 E	10m	Epizoic	Multiporates
Hapalidiales sp. YE	Hapalidiales	NZC2354	FJ361576	Auckland, Pihakoa Point	34.83 S 173.452 E	10m	Epizoic	Multiporates
Hapalidiales sp. YE	Hapalidiales	NZC2365	KM369021	Auckland, North Cape	34.418 S 173.052 E	8m	Epizoic	Uniporates

Hapalidiales sp. YE	Hapalidiales	NZC2365	KM369021	Auckland, North Cape	34.418 S 173.052 E	8m	Epizoic	Uniporates
Hapalidiales sp. YF	Hapalidiales	NZC0234	DQ168001	Nelson, Ataata Point	41.157 S 173.408 E	-	Encrusting	Uniporates
Hapalidiales sp. YF	Hapalidiales	NZC0239	DQ167870	Nelson, Ataata Point	41.157 S 173.408 E	-	Encrusting	Uniporates
Hapalidiales sp. YF	Hapalidiales	NZC0239	DQ167870	Nelson, Ataata Point	41.157 S 173.408 E	-	Encrusting	Uniporates
Hapalidiales sp. YF	Hapalidiales	NZC0251	DQ167871	Nelson, Wharariki Beach	40.5 S 172.677 E	-	Encrusting	Uniporates
Hapalidiales sp. YF	Hapalidiales	NZC0621	DQ168004	Canterbury, Taylor's Mistake	43.585 S 172.774 E	5m	Epizoic	Uniporates
Hapalidiales sp. YF	Hapalidiales	NZC0744	DQ167928	Chatham Island, Port Hutt	43.816 S 176.705 W	-	Encrusting	Multiporates
Hapalidiales sp. YF	Hapalidiales	NZC0746	DQ167930	Chatham Island, Whangatete Inlet	43.798 S 176.684 W	-	Warty	Multiporates
Hapalidiales sp. YF	Hapalidiales	NZC0754	EF628224	Chatham Island, Whangatete Inlet	43.798 S 176.684 W	-	Warty	Multiporates
Hapalidiales sp. YF	Hapalidiales	NZC1060	MK413629	Wellington, Ranger Point	41.34 S 174.825 E	-	Encrusting	Multiporates
Hapalidiales sp. YF	Hapalidiales	NZC1063	MK413628	Wellington, Ranger Point	41.34 S 174.825 E	-	Encrusting	Multiporates
Hapalidiales sp. YF	Hapalidiales	NZC1064	MK413627	Wellington, Ranger Point	41.34 S 174.825 E	-	Encrusting	Multiporates
Hapalidiales sp. YF	Hapalidiales	NZC1065	MK413626	Wellington, Ranger Point	41.34 S 174.825 E	-	Encrusting	Multiporates
Hapalidiales sp. YF	Hapalidiales	NZC2017	FJ361385	Auckland, Te Henga	36.887 S 174.437 E	-	Encrusting	Multiporates
Hapalidiales sp. YF	Hapalidiales	NZC2084	FJ361454	Auckland, Sandy Bay	35.557 S 174.479 E	-	Encrusting	Multiporates-Volcano
Hapalidiales sp. YF	Hapalidiales	NZC2104	FJ361436	Auckland, Matai Bay	34.828 S 173.411 E	-	Encrusting	Multiporates
Hapalidiales sp. YF	Hapalidiales	NZC2112	FJ361435	Auckland, Matai Bay	34.828 S 173.411 E	-	Encrusting	Multiporates
Hapalidiales sp. YF	Hapalidiales	NZC2117	FJ361421	Auckland, Matai Bay	34.828 S 173.411 E	-	Epilithic	Uniporates
Hapalidiales sp. YF	Hapalidiales	NZC2172	FJ361504	Auckland, Whale Bay	37.822 S 174.802 E	-	Warty	Multiporates-Volcano
Hapalidiales sp. YF	Hapalidiales	NZC2176	FJ361665	Auckland, Whale Bay	37.822 S 174.802 E	-	Fruticose	Multiporates
Hapalidiales sp. YF	Hapalidiales	NZC2187	FJ361558	Auckland, Tapeka Beach	35.243 S 174.118 E	-		-
Hapalidiales sp. YF	Hapalidiales	NZC2203	FJ361636	Auckland, Bland Bay	35.346 S 174.367 E	-	Lumpy	Uniporates
Hapalidiales sp. YF	Hapalidiales	NZC2205	FJ361534	Auckland, Bland Bay	35.346 S 174.367 E	-	Warty	Uniporates
Hapalidiales sp. YF	Hapalidiales	NZC2213	FJ361483	Auckland, Rangiatea	35.217 S 174.181 E	13m	Epiphytic	Multiporates
Hapalidiales sp. YF	Hapalidiales	NZC2235	FJ361533	Auckland, Rangiatea	35.217 S 174.181 E	13m	Epizoic	Multiporates
Hapalidiales sp. YF	Hapalidiales	NZC2252	FJ361522	Auckland, Rangiatea	35.217 S 174.181 E	13m	Warty	Multiporates
Hapalidiales sp. YF	Hapalidiales	NZC2253	FJ361498	Auckland, Rangiatea	35.217 S 174.181 E	13m	Encrusting	Multiporates
Hapalidiales sp. YF	Hapalidiales	NZC2292	FJ361542	Auckland, Okahu Channel	35.197 S 174.216 E	14m	Encrusting	Multiporates-Volcano
Hapalidiales sp. YF	Hapalidiales	NZC2305	FJ361482	Auckland, Urupukapuka	35.205 S 174.234 E	14m	Encrusting	Multiporates
Hapalidiales sp. YF	Hapalidiales	NZC2342	KM369020	Auckland, Pihakoa Point	34.83 S 173.452 E	17m	Encrusting	Multiporates
Hapalidiales sp. YF	Hapalidiales	NZC2349	FJ361626	Auckland, Pihakoa Point	34.83 S 173.452 E	10m	Encrusting	Multiporates
Hapalidiales sp. YF	Hapalidiales	NZC2351	FJ361623	Auckland, Pihakoa Point	34.83 S 173.452 E	10m	Encrusting	Multiporates
Hapalidiales sp. YF	Hapalidiales	NZC2408	FJ361595	Auckland, Wekarua Is.	34.936 S 173.654 E	10m	Encrusting	Uniporates

Hapalidiales sp. YF	Hapalidiales	NZC2416	FJ361663	Auckland, Sailors Grave	36.961 S 175.844 E	-	Warty	Uniporates
Hapalidiales sp. YF	Hapalidiales	NZC2422	FJ361632	Auckland, Sailors Grave	36.961 S 175.844 E	-	Lumpy	Multiporates
Hapalidiales sp. YF	Hapalidiales	NZC2440	FJ361659	Auckland, Little Bay	36.601 S 175.549 E	-	Epiphytic	Multiporates
Hapalidiales sp. YF	Hapalidiales	NZC2443	FJ361649	Auckland, Little Bay	36.601 S 175.549 E	-	Warty	Multiporates
Hapalidiales sp. YF	Hapalidiales	NZC2457	FJ361647	Auckland, Fletcher Bay	36.475 S 175.392 E	-	Encrusting	Multiporates
Hapalidiales sp. YF	Hapalidiales	NZC2459	FJ361654	Auckland, Fletcher Bay	36.475 S 175.392 E	-	Lumpy	Uniporates
Hapalidiales sp. YF	Hapalidiales	NZC2466	FJ361648	Auckland, Fletcher Bay	36.475 S 175.392 E	-	Encrusting	Multiporates-Volcano
Hapalidiales sp. YF	Hapalidiales	NZC2467	FJ361650	Auckland, Fletcher Bay	36.475 S 175.392 E	-	Warty	Multiporates
Hapalidiales sp. YF	Hapalidiales	NZC2507	FJ361751	Auckland, Henderson Point	34.741 S 173.118 E	-	Encrusting	Multiporates
Hapalidiales sp. YF	Hapalidiales	NZC2508-1	FJ361696	Auckland, Henderson Point	34.741 S 173.118 E	-	Encrusting	Multiporates
Hapalidiales sp. YF	Hapalidiales	NZC2548	FJ361735	Auckland, Kapowairua	34.421 S 172.856 E	-	Encrusting	Multiporates
Hapalidiales sp. YF	Hapalidiales	NZC2551	FJ361715	Auckland, Kapowairua	34.421 S 172.856 E	-	Warty	Multiporates-Volcano
Hapalidiales sp. YF	Hapalidiales	NZC2577	FJ361711	Auckland, Tapotupotu Bay	34.435 S 172.717 E	-	Warty	Multiporates-Volcano
Hapalidiales sp. YF	Hapalidiales	NZC2580-1	FJ361704	Auckland, Tapotupotu Bay	34.435 S 172.717 E	-	Warty	Multiporates
Hapalidiales sp. YF	Hapalidiales	NZC2580-2	FJ361710	Auckland, Tapotupotu Bay	34.435 S 172.717 E	-	Warty	Multiporates
Hapalidiales sp. YF	Hapalidiales	NZC2588	FJ361690	Auckland, Tapotupotu Bay	34.435 S 172.717 E	-	Warty	Multiporates
Hapalidiales sp. YF	Hapalidiales	NZC2611	FJ361728	Gisborne, Lottin Point	37.553 S 178.164 E	-	Fruticose	Multiporates
Hapalidiales sp. YG	Hapalidiales	NZC5241	MK413491	Southland, Waikawa, Waipapa	46.661 S 168.846 E	0m	Warty	Multiporates - Flat top
Hapalidiales sp. Z	Hapalidiales	DH6	DQ168025	Otago	-	-	-	-
Hapalidiales sp. Z	Hapalidiales	NZC0899	KM369015	Wellington, Is. Bay	41.35 S 174.767 E	0m	Encrusting	Uniporates
Hapalidiales sp. Z	Hapalidiales	NZC4001a	MK413595	Antipodes Islands, Archway Is.	-	-	Epilithic	Multiporates - Flat top
Hapalidiales sp. Z	Hapalidiales	NZC5020	MK413582	Otago, Brighton	45.949 S 170.335 E	0m	Epiphytic	Multiporates - Flat top
Hapalidiales sp. Z	Hapalidiales	NZC5141	MK413533	Auckland Islands, Musgrave	50.65 S 166.154 E	0m	Warty	Multiporates - Flat top
Hapalidiales sp. Z	Hapalidiales	NZC5142A	MK413532	Auckland Islands, Musgrave	50.65 S 166.154 E	0m	Warty	Multiporates - Flush
Hapalidiales sp. Z	Hapalidiales	NZC5142B	MK413531	Auckland Islands, Musgrave	50.65 S 166.154 E	0m	Warty	Uniporates- Dome
Hapalidiales sp. Z	Hapalidiales	NZC5156A	MK413525	Fiordland, Turn Round Point	44.8 S 167.544 E	5m	Discoid- Epiphytic	Uniporates
Hapalidiales sp. Z	Hapalidiales	NZC5222	MK413503	Otago, Kaka Point, Tawhiri	46.431 S 169.798 E	0m	Discoid- Epiphytic	Uniporates- Dome
Hapalidiales sp. Z	Hapalidiales	NZC5245	MK413489	Southland, Waikawa, Porpoise Bay	46.661 S 169.108 E	0m	Discoid- Epiphytic	Uniporates- Dome
Hapalidiales sp. Z	Hapalidiales	NZC5262	MK413476	Southland, Riverton Rocks	46.371 S 168.028 E	0m	Discoid- Epiphytic	-
Hapalidiales sp. Z	Hapalidiales	NZC5330	MK413434	Westland, Jackson Bay	43.972 S 168.616 E	0m	Encrusting-Epiphytic	Multiporates - Flat top
Hapalidiales sp. Z	Hapalidiales	NZC5337	MK413427	Westland, Ocean Beach	43.966 S 168.607 E	0m	Discoid- Epiphytic	Uniporates- Dome

Hapalidiales sp. Z	Hapalidiales	NZC5338	MK413426	Westland, Ocean Beach	43.966 S 168.607 E	0m	Discoid- Epiphytic	Uniporates- Flush
Hapalidiales sp. Z	Hapalidiales	NZC5347	MK413419	Otago, Chaslands	46.625 S 169.361 E	2m	Discoid- Epiphytic	Uniporates- Dome
Hapalidiales sp. Z	Hapalidiales	NZC5492	MK413348	Otago, Dundein, Akatore	46.112 S 170.192 E	0m	Discoid- Epiphytic	Uniporates- Pointy
Hapalidiales sp. Z	Hapalidiales	NZC5493	MK413347	Otago, Dundein, Akatore	46.112 S 170.192 E	0m	Discoid- Epizoic	Uniporates- Pointy
Hapalidiales sp. Z	Hapalidiales	NZC5512	MK413336	Otago, Green Island	45.952 S 170.386 E	0m	Discoid	Uniporates- Dome
Hapalidiales sp. ZA	Hapalidiales	NZC5368	MK413409	Stewart Island, Horseshoe Bay	46.878 S 168.148 E	2m	Epilithic	Multiporates - Volcano
Hapalidiales sp. ZA	Hapalidiales	NZC5433	MK413373	Stewart Is., Black & White Bay	46.692 S 167.888 E	2m	Lumpy	Multiporates - Volcano
Hapalidiales sp. ZB	Hapalidiales	NZC5697	MK413221	Fiordland, Small Craft Harbour Is.	45.967 S 166.651 E	5m	Encrusting	-
Hapalidiales sp. ZB	Hapalidiales	NZC5697	MK413221	Fiordland, Small Craft Harbour Is.	45.967 S 166.651 E	5m	Encrusting	-
Hapalidiales sp. ZC	Hapalidiales	NZC0018	DQ167880	Wellington, Is. Bay	41.349 S 174.763 E	5m	Encrusting	Multiporates
Hapalidiales sp. ZC	Hapalidiales	NZC0476	KM369013	Nelson, Taupo Point	40.787 S 172.957 E	4m	Epizoic	Multiporates
Hapalidiales sp. ZD	Hapalidiales	NZC5080	MK413550	Southland, Foveaux strait	46.68 S 167.999 E	38m	Encusting	Uniporates
Hapalidiales sp. ZD	Hapalidiales	NZC5087	MK413546	Southland, Foveaux strait	46.658 S 167.973 E	48m	Warty	-
Hapalidiales sp. ZD	Hapalidiales	NZC5124	MK413540	Fiordland, Five Fingers Pen.	45.712 S 166.5 E	6m	Encusting	Multiporates - Flat top
Hapalidiales sp. ZD	Hapalidiales	NZC5125A	MK413539	Fiordland, Turn Round Point	44.8 S 167.544 E	5m	Encusting	Multiporates - Flat top
Hapalidiales sp. ZD	Hapalidiales	NZC5654B	MK413241	Fiordland, Narrow Bend	46.067 S 166.734 E	1m	Encrusting	Uniporates- Pointy
Hapalidiales sp. ZE	Hapalidiales	NZC5469	MK413360	Southland, Bluff, site 24023	46.581 S 168.33 E	5m	Fruticose	Multiporates - Flat top
Hapalidiales sp. ZF	Hapalidiales	NZC5361	MK413414	Stewart Island, Horseshoe Bay	46.878 S 168.148 E	2m	Lumpy	-
Hapalidiales sp. ZG	Hapalidiales	NZC5425A	MK413381	Stewart Is., Black & White Bay	46.692 S 167.888 E	0m	Warty	Multiporates - Flush
Hapalidiales sp. ZH	Hapalidiales	NZC5144	MK413530	Auckland Islands, Hanfield	50.736 S 166.142 E	13m	Warty	Uniporates
Hapalidiales sp. ZH	Hapalidiales	NZC5145B	MK413529	Auckland Islands, Norman Inlet	50.711 S 166.145 E	22m	Encusting	Uniporates
Hapalidiales sp. ZH	Hapalidiales	NZC5147	MK413528	Auckland Islands, Norman Inlet	50.711 S 166.145 E	22m	Warty	-
Hapalidiales sp. ZH	Hapalidiales	NZC5501	MK413342	Otago, Green Island	45.952 S 170.386 E	0m	Lumpy	Multiporates - Flush
Hapalidiales sp. ZH	Hapalidiales	NZC5504	MK413341	Otago, Green Island	45.952 S 170.386 E	0m	Lumpy	Multiporates - Flush
Hapalidiales sp. ZH	Hapalidiales	NZC5505	MK413340	Otago, Green Island	45.952 S 170.386 E	0m	Encrusting	Multiporates - Flush
Hapalidiales sp. ZH	Hapalidiales	NZC5508	MK413337	Otago, Green Island	45.952 S 170.386 E	0m	Lumpy	Multiporates - Flat top
Hapalidiales sp. ZH	Hapalidiales	NZC5618	MK413255	Fiordland, Cavern Head	46.081 S 166.648 E	3m	Lumpy	Multiporates - Flat top
Hapalidiales sp. ZH	Hapalidiales	NZC5692	MK413223	Fiordland, North Port	45.977 S 166.586 E	5m	Fruticose	Uniporates- Dome
Hapalidiales sp. ZI	Hapalidiales	NZC5079	MK413551	Southland, Foveaux strait	46.68 S 167.999 E	38m	Warty	Multiporates - Volcano
Hapalidiales sp. ZI	Hapalidiales	NZC5086	MK413547	Southland, Foveaux strait	46.715 S 168.086 E	0	Encusting	Uniporates
Hapalidiales sp. ZI	Hapalidiales	NZC5090	MK413545	Southland, Foveaux strait	46.697 S 168.09 E	0	Encusting	-
Hapalidiales sp. ZJ	Hapalidiales	CUK18093	MK702010	Stewart Is., Black & White Bay	46.692 S 167.888 E	10m	-	-

Hapalidiales sp. ZK	Hapalidiales	NZC5354	MK413416	Otago, Chaslands	46.625 S 169.361 E	2m	Encrusting-Epiphytic	Multiporates - Volcano
Hapalidiales sp. ZL	Hapalidiales	NZC5429	MK413377	Stewart Is., Black & White Bay	46.692 S 167.888 E	0m	Epilithic	Uniporates- Dome
Hapalidiales sp. ZM	Hapalidiales	CUK18097	MK702011	Stewart Island, Caroline Bay	46.755 S 168.493 E	0m	-	-
Hapalidiales sp. ZN	Hapalidiales	NZC5548	MK413316	Otago, Moeraki	45.362 S 170.863 E	1m	Warty	-
Hapalidiales sp. ZO	Hapalidiales	NZC2371	FJ361610	Auckland, North Cape	34.418 S 173.052 E	8m	Encrusting	Multiporates-Volcano
Hapalidiales sp. ZP	Hapalidiales	NZC5698A	MK413220	Fiordland, Small Craft Harbour Is.	45.967 S 166.651 E	5m	Warty	Multiporates - Flat top
Hapalidiales sp. ZQ	Hapalidiales	NZC5095	MK413544	Southland, Foveaux strait	46.742 S 168.14 E	0	Encusting	-
Hapalidiales sp. ZR	Hapalidiales	NZC0260	DQ167873	Nelson, Wharariki Beach	40.5 S 172.676 E	-	Encrusting	Multiporates
Hapalidiales sp. ZR	Hapalidiales	NZC0785	DQ167945	Chatham Island, Te One Creek	44.019 S 176.383 W	-	Encrusting	Uniporates
Hapalidiales sp. ZR	Hapalidiales	NZC5084B	MK413548	Southland, Foveaux strait	46.697 S 168.045 E	0	Encusting	-
Hapalidiales sp. ZR	Hapalidiales	NZC5308B	MK413450	Otago, Moeraki, Katiki Point W	45.397 S 170.868 E	3m	Encrusting	-
Hapalidiales sp. ZR	Hapalidiales	NZC5427	MK413378	Stewart Is., Black & White Bay	46.692 S 167.888 E	2m	Epilithic	Multiporates - Flush
Hapalidiales sp. ZR	Hapalidiales	NZC5468B	MK413361	Southland, Bluff, site 24250	46.601 S 168.351 E	5m	Encrusting	Multiporates - Flush
Hapalidiales sp. ZS	Hapalidiales	NZC0762	DQ167950	Chatham Island, Okawa Point	43.77 S 176.247 W	-	Encrusting	Multiporates
Hapalidiales sp. ZS	Hapalidiales	NZC0772	EF628215	Chatham Island, Heaphy Shoal	43.966 S 176.593 W	-	Warty	Multiporates
Hapalidiales sp. ZS	Hapalidiales	NZC2008	FJ361377	Auckland, Little Huia	37.011 S 174.562 E	-	Encrusting	Uniporates
Hapalidiales sp. ZS	Hapalidiales	NZC2011	FJ361380	Auckland, Cornwallis Wharf	37.012 S 174.605 E	-	Encrusting	Uniporates
Hapalidiales sp. ZS	Hapalidiales	NZC2059	FJ361424	Auckland, Mathesons Bay	36.302 S 174.798 E	-	Encrusting	Uniporates
Hapalidiales sp. ZS	Hapalidiales	NZC2076	FJ361452	Auckland, Ngunguru Harbour	35.636 S 174.5 E	-	Warty	Uniporates
Hapalidiales sp. ZS	Hapalidiales	NZC2093	FJ361434	Auckland, Urquhart's Bay	35.502 S 174.533 E	-	Encrusting	Uniporates
Hapalidiales sp. ZS	Hapalidiales	NZC2156	MK413614	Auckland, Kiritehere Beach	38.325 S 174.703 E	-	Geniculate	-
Hapalidiales sp. ZS	Hapalidiales	NZC2208	FJ361506	Auckland, Bland Bay	35.346 S 174.367 E	-	Encrusting	Uniporates
Hapalidiales sp. ZS	Hapalidiales	NZC5327	MK413436	Westland, Jackson Bay	43.972 S 168.616 E	0m	Lumpy	Multiporates - Flat top
Hapalidiales sp. ZS	Hapalidiales	NZC5534	MK413328	Otago, Dunedin, Quarantine Is.	45.828 S 170.637 E	0m	Warty	Uniporates- Dome
Hapalidiales sp. ZT	Hapalidiales	NZC0087	EF628216	Marlborough, Halfmoon Bay	42.267 S 173.8 E	-	Encrusting	Multiporates
Hapalidiales sp. ZT	Hapalidiales	NZC0094	DQ167908	Marlborough, Rakautara BBQ	42.27 S 173.8 E	0m	Encrusting	Multiporates
Hapalidiales sp. ZT	Hapalidiales	NZC0396	DQ167890	Auckland, Kaiti Beach	38.684 S 178.032 E	-	Encrusting	Uniporates
Hapalidiales sp. ZT	Hapalidiales	NZC0404	DQ167891	Auckland, Kaiti Beach	38.684 S 178.032 E	-	Encrusting	Multiporates
Hapalidiales sp. ZT	Hapalidiales	NZC0450	DQ167899	Nelson, Cable Bay	41.156 S 173.403 E	7m	Encrusting	Uniporates
Hapalidiales sp. ZT	Hapalidiales	NZC0457	DQ167920	Nelson, Taupo Rocks	40.784 S 172.961 E	8m	Encrusting	Uniporates
Hapalidiales sp. ZT	Hapalidiales	NZC0514	DQ167904	Nelson, Tata Rocks Limestone	40.791 S 172.952 E	5m	Encrusting	Multiporates
Hapalidiales sp. ZT	Hapalidiales	NZC0740	DQ167924	Chatham Island, Port Hutt	43.816 S 176.705 W	-	Encrusting	Multiporates
Hapalidiales sp. ZT	Hapalidiales	NZC0741	DQ167925	Chatham Island, Point Durham	44 S 176.675 W	-	Encrusting	Multiporates

Hapalidiales sp. ZT	Hapalidiales	NZC0774	DQ167953	Chatham Island, Tommy Solomon Monument	44.032 S 176.337 W	0m	Encrusting	Multiporates
Hapalidiales sp. ZT	Hapalidiales	NZC0776	DQ167949	Chatham Island, Wharekauri	43.707 S 176.574 W	-	Encrusting	-
Hapalidiales sp. ZT	Hapalidiales	NZC0812	DQ167973	Hawkes Bay, Mangakuri Beach	39.966 S 176.922 E	0m	Encrusting	Multiporates
Hapalidiales sp. ZT	Hapalidiales	NZC2032	FJ361395	Auckland, Rocky Bay	36.819 S 175.053 E	0m	Encrusting	Multiporates-Volcano
Hapalidiales sp. ZT	Hapalidiales	NZC2072	FJ361462	Auckland, Lang's Beach	36.043 S 174.532 E	-	Lumpy	-
Hapalidiales sp. ZT	Hapalidiales	NZC2094	FJ361423	Auckland, Urquhart's Bay	35.502 S 174.533 E	-	Encrusting	Uniporates
Hapalidiales sp. ZT	Hapalidiales	NZC2137	MK413617	Auckland, Cable Bay	34.99 S 173.487 E	-	Epiphytic	Uniporates
Hapalidiales sp. ZT	Hapalidiales	NZC2137	MK413617	Auckland, Cable Bay	34.99 S 173.487 E	-	Epiphytic	Uniporates
Hapalidiales sp. ZT	Hapalidiales	NZC2146	FJ361450	Auckland, Cable Bay	34.99 S 173.487 E	-	Warty	Uniporates
Hapalidiales sp. ZT	Hapalidiales	NZC2335	MK413608	Auckland, Wekarua Is.	34.936 S 173.654 E	10m	Encrusting	Uniporates
Hapalidiales sp. ZT	Hapalidiales	NZC2335	MK413608	Auckland, Wekarua Is.	34.936 S 173.654 E	10m	Encrusting	Uniporates
Hapalidiales sp. ZT	Hapalidiales	NZC2495	MK413602	Auckland, Ahipara	35.175 S 173.117 E	-	Geniculate	-
Hapalidiales sp. ZT	Hapalidiales	NZC2501	KM369023	Auckland, Ahipara	35.175 S 173.117 E	-	Encrusting	Multiporates
Hapalidiales sp. ZT	Hapalidiales	NZC2557	FJ361679	Auckland, The Bluff	34.685 S 172.89 E	-	Encrusting	Uniporates
Hapalidiales sp. ZT	Hapalidiales	NZC5024	MK413578	Otago, Butterfly Bay	45.638 S 170.672 E	1m	Epilithic	Multiporates - Flat top
Hapalidiales sp. ZT	Hapalidiales	NZC5039	MK413571	Otago, Butterfly Bay	45.639 S 170.672 E	0m	Epilithic	-
Hapalidiales sp. ZT	Hapalidiales	NZC5056	MK413562	Southland, Crombie	46.257 S 167.164 E	0m	Epilithic	-
Hapalidiales sp. ZT	Hapalidiales	NZC5069	MK413557	Southland, Snout	46.26 S 167.194 E	0m	Encusting	-
Hapalidiales sp. ZT	Hapalidiales	NZC5076B	MK413552	Southland, Bluff	46.601 S 168.358 E	6m	Encusting	-
Hapalidiales sp. ZT	Hapalidiales	NZC5259	MK413479	Southland, Riverton, Cosy Nook	46.331 S 167.72 E	0m	Epilithic	Multiporates - Flush
Hapalidiales sp. ZT	Hapalidiales	NZC5325	MK413438	Westland, Jackson Bay	43.972 S 168.616 E	0m	Encrusting	-
Hapalidiales sp. ZT	Hapalidiales	NZC5326	MK413437	Westland, Jackson Bay	43.972 S 168.616 E	0m	Encrusting	Multiporates - Flush
Hapalidiales sp. ZT	Hapalidiales	NZC5394	MK413396	Stewart Island, Passage Rock	47.078 S 168.205 E	2m	Encrusting	Multiporates - Flush
Hapalidiales sp. ZT	Hapalidiales	NZC5436	MK413371	Stewart Is., Black & White Bay	46.692 S 167.888 E	2m	Epilithic	Multiporates - Flush
Hapalidiales sp. ZT	Hapalidiales	NZC5441	MK413368	Stewart Island, White Rock E	46.69 S 167.882 E	4m	Epilithic	Multiporates - Flush
Hapalidiales sp. ZT	Hapalidiales	NZC5445	MK413367	Stewart Island, White Rock E	46.686 S 167.878 E	5m	Epilithic	Multiporates - Flush
Hapalidiales sp. ZT	Hapalidiales	NZC5486	MK413351	Otago, Dundein, Akatore	46.112 S 170.192 E	0m	Encrusting	Multiporates - Volcano
Hapalidiales sp. ZT	Hapalidiales	NZC5539	MK413325	Otago, Moeraki	45.362 S 170.863 E	1m	Encrusting	Multiporates - Flush
Hapalidiales sp. ZT	Hapalidiales	NZC5558	MK413308	Otago, Moeraki	45.362 S 170.863 E	1m	Encrusting	Multiporates - Flush
Hapalidiales sp. ZT	Hapalidiales	NZC5583	MK413285	Otago, Moeraki	45.362 S 170.863 E	1m	Epilithic	-
Hapalidiales sp. ZT	Hapalidiales	NZC5629	MK413249	Fiordland, Spit Islands	46.072 S 166.634 E	0m	Epilithic	Multiporates - Flush
Hapalidiales sp. ZT	Hapalidiales	NZC5650	MK413243	Fiordland, Narrow Bend	46.067 S 166.734 E	1m	Encrusting	Multiporates - Flush
Hapalidiales sp. ZT	Hapalidiales	NZC5657B	MK413239	Fiordland, Straggle Head	46.032 S 166.546 E	1m	Encrusting	Multiporates - Flush

Hapalidiales sp. ZT	Hapalidiales	NZC5659	MK413237	Fiordland, Straggle Head	46.032 S 166.546 E	1m	Encrusting	Multiporates - Flush
Hapalidiales sp. ZT	Hapalidiales	NZC5703	MK413219	Fiordland, Small Craft Harbour Is.	45.967 S 166.651 E	5m	Warty- Epizoic	Multiporates - Flush
Hapalidiales sp. ZT	Hapalidiales	NZC5711	MK413217	Fiordland, North Port	46.035 S 166.592 E	1m	Lumpy	Multiporates - Flush
Hapalidiales sp. ZT	Hapalidiales	P11	MK413201	Wellington, Ranger Point	41.34 S 174.825 E	0m	-	-
Hapalidiales sp. ZT	Hapalidiales	P23	MK413200	Wellington, Ranger Point	41.34 S 174.825 E	0m	-	-
Hapalidiales sp. ZT	Hapalidiales	P4	MK413202	Wellington, Ranger Point	41.34 S 174.825 E	0m	-	-
Hapalidiales sp. ZU	Hapalidiales	NZC5140	MK413534	Auckland Islands, Musgrave	50.65 S 166.154 E	0m	Encusting	Uniporates
Hapalidiales sp. ZU	Hapalidiales	NZC5176	MK413517	Auckland Islands, Tagua Bay	50.809 S 166.07 E	0m	Warty	Uniporates
Hapalidiales sp. ZU	Hapalidiales	NZC5177	MK413516	Auckland Islands, Tagua Bay	50.809 S 166.07 E	0m	Encrusting	Uniporates
Hapalidiales sp. ZU	Hapalidiales	NZC5180	MK413515	Auckland Islands, Tagua Bay	50.809 S 166.07 E	0m	Encrusting	Uniporates
Hapalidiales sp. ZU	Hapalidiales	NZC5449	MK413364	Southland, Caroline Bay	46.755 S 168.493 E	0m	Epilithic	Uniporates- Dome
Hapalidiales sp. ZU	Hapalidiales	NZC5628	MK413250	Fiordland, Spit Islands	46.072 S 166.634 E	0m	Encrusting	Uniporates- Dome
Hapalidiales sp. ZV	Hapalidiales	NZC5221	MK413504	Otago, Kaka Point, Tawhiri	46.431 S 169.798 E	0m	Lumpy	Multiporates - Volcano
Hapalidiales sp. ZW	Hapalidiales	NZC2317	MK413609	Southland, Foveaux Strait	46.683 S 168.15 E	-	-	-
Hapalidiales sp. ZX	Hapalidiales	NZC5038	MK413572	Otago, Butterfly Bay	45.639 S 170.672 E	0m	Epilithic	Multiporates - Volcano
Hapalidiales sp. ZX	Hapalidiales	NZC5045	MK413569	Otago, Wellers Rock	45.798 S 170.715 E	0m	Epizoic	-
Hapalidiales sp. ZX	Hapalidiales	NZC5223	MK413502	Otago, Kaka Point, Tawhiri	46.431 S 169.798 E	0m	Warty	Multiporates - Flat top
Hapalidiales sp. ZX	Hapalidiales	NZC5227	MK413500	Otago, Kaka Point, Tawhiri	46.428 S 169.796 E	0m	Epizoic	Multiporates - Volcano
Hapalidiales sp. ZX	Hapalidiales	NZC5297	MK413455	Otago, Moeraki, Katiki Point E	45.397 S 170.869 E	0m	Epilithic	Uniporates- Flush
Hapalidiales sp. ZX	Hapalidiales	NZC5315	MK413446	Westland, Jackson Bay	43.972 S 168.616 E	0m	Warty	-
Hapalidiales sp. ZX	Hapalidiales	NZC5425B	MK413380	Stewart Is., Black & White Bay	46.692 S 167.888 E	0m	Warty- Epizoic	Multiporates - Volcano
Hapalidiales sp. ZY	Hapalidiales	NZC0240	DQ168002	Nelson, Cable Bay	41.156 S 173.403 E	11m	Epizoic	Multiporates
Hapalidiales sp. ZY	Hapalidiales	NZC0265	EF628220	Nelson, Patons Rock	40.786 S 172.766 E	0m	Encrusting	Multiporates
Hapalidiales sp. ZY	Hapalidiales	NZC0452	EF628221	Nelson, Taupo Point	40.787 S 172.957 E	4m	Epizoic	Multiporates
Hapalidiales sp. ZY	Hapalidiales	NZC0710	DQ167978	Chatham Island, Port Hutt	43.816 S 176.705 W	-	Encrusting	Multiporates
Hapalidiales sp. ZY	Hapalidiales	NZC0732	DQ167922	Chatham Island, Te One Creek	44.019 S 176.383 W	0m	Epizoic	Multiporates
Hapalidiales sp. ZY	Hapalidiales	NZC0755	DQ167936	Chatham Island, Whangatete Inlet	43.798 S 176.684 W	-	Encrusting	Multiporates
Hapalidiales sp. ZY	Hapalidiales	NZC0827	DQ167974	Wellington, Kapiti Is.	40.87 S 174.921 E	7m	Encrusting	Uniporates
Hapalidiales sp. ZY	Hapalidiales	NZC0882	DQ167986	Wellington, Kapiti Is.	40.87 S 174.921 E	7m	Warty	Multiporates
Hapalidiales sp. ZY	Hapalidiales	NZC2079	FJ361420	Auckland, Tutukaka	35.612 S 174.532 E	-	Epizoic	Multiporates
Hapalidiales sp. ZY	Hapalidiales	NZC2433-A	FJ361633	Auckland, Little Bay	36.601 S 175.549 E	-	Epizoic	Uniporates

Hapalidiales sp. ZY	Hapalidiales	NZC2433-A	FJ361633	Auckland, Little Bay	36.601 S 175.549 E	-	Epizoic	Uniporates
Hapalidiales sp. ZY	Hapalidiales	NZC2450	MK413603	Auckland, Little Bay	36.601 S 175.549 E	-	Geniculate	-
Hapalidiales sp. ZY	Hapalidiales	NZC2450	MK413603	Auckland, Little Bay	36.601 S 175.549 E	-	Geniculate	-
Hapalidiales sp. ZY	Hapalidiales	NZC2585	FJ361724	Auckland, Tapotupotu Bay	34.435 S 172.717 E	-	Warty	Multiporates
Hapalidiales sp. ZY	Hapalidiales	NZC5076A	MK413553	Southland, Bluff	46.601 S 168.358 E	6m	Warty	Multiporates - Volcano
Hapalidiales sp. ZY	Hapalidiales	NZC5397B	MK413394	Stewart Island, Port Adventure	47.07 S 168.219 E	15m	Epiltihic	Multiporates - Flat top
Hapalidiales sp. ZY	Hapalidiales	NZC5454	MK413363	Southland, Caroline Bay	46.755 S 168.493 E	2m	Warty	Multiporates - Flat top
Hapalidiales sp. ZY	Hapalidiales	NZC5609	MK413259	Otago, Moeraki	45.362 S 170.863 E	1m	Lumpy	Multiporates - Flat top
Hapalidiales sp. ZZ	Hapalidiales	NZC0233	DQ167889	Nelson, Ataata Point	41.157 S 173.408 E	-	Epizoic	Multiporates
Hapalidiales sp. ZZ	Hapalidiales	NZC0244	DQ167888	Nelson, Wharariki Beach	40.5 S 172.676 E	-	Encrusting	Multiporates
Hapalidiales sp. ZZ	Hapalidiales	NZC0312	EF628219	Nelson, Wharariki Beach	40.5 S 172.677 E	-	Encrusting	Multiporates
Hapalidiales sp. ZZ	Hapalidiales	NZC0320	DQ167956	Nelson, Ataata Point	41.157 S 173.408 E	0m	Encrusting	Uniporates
Hapalidiales sp. ZZ	Hapalidiales	NZC0444	DQ167898	Nelson, Cable Bay	41.156 S 173.403 E	11m	Epizoic	Multiporates
Hapalidiales sp. ZZ	Hapalidiales	NZC0446	DQ167957	Nelson, Tata Rocks Limestone	40.791 S 172.952 E	5m	Epizoic	Multiporates
Hapalidiales sp. ZZ	Hapalidiales	NZC0451	DQ167894	Nelson, Cable Bay	41.156 S 173.403 E	7m	Epizoic	Multiporates
Hapalidiales sp. ZZ	Hapalidiales	NZC0458	DQ167977	Nelson, Taupo Point	40.787 S 172.957 E	4m	Encrusting	Multiporates
Hapalidiales sp. ZZ	Hapalidiales	NZC0484	DQ167892	Nelson, Whare North Rocks	40.781 S 172.983 E	8m	Epizoic	Multiporates
Hapalidiales sp. ZZ	Hapalidiales	NZC0788	DQ167951	Hawkes Bay, Tuingara Point	40.121 S 176.875 E	5m	Encrusting	Multiporates
Hapalidiales sp. ZZ	Hapalidiales	NZC0794	DQ167980	Hawkes Bay, Tuingara Point	40.121 S 176.875 E	15m	Encrusting	Multiporates
Hapalidiales sp. ZZ	Hapalidiales	NZC0835	DQ167996	Wellington, Kapiti Is.	40.87 S 174.921 E	16m	Warty	Uniporates
Hapalidiales sp. ZZ	Hapalidiales	NZC0858	DQ167983	Wellington, Kapiti Is.	40.87 S 174.921 E	7m	Encrusting	Uniporates
Hapalidiales sp. ZZ	Hapalidiales	NZC2018	FJ361386	Auckland, Te Henga	36.887 S 174.437 E	-	Encrusting	Uniporates
Hapalidiales sp. ZZ	Hapalidiales	NZC2030	FJ361394	Auckland, Rocky Bay	36.819 S 175.053 E	-	-	Uniporates
Hapalidiales sp. ZZ	Hapalidiales	NZC2031	FJ361396	Auckland, Rocky Bay	36.819 S 175.053 E	-	Encrusting	-
Hapalidiales sp. ZZ	Hapalidiales	NZC2033	FJ361397	Auckland, Rocky Bay	36.819 S 175.053 E	-	Lumpy	Multiporates-Volcano
Hapalidiales sp. ZZ	Hapalidiales	NZC2035	FJ361670	Auckland, Rocky Bay	36.819 S 175.053 E	-	Encrusting	Multiporates- Flat top
Hapalidiales sp. ZZ	Hapalidiales	NZC2051	KM369019	Auckland, Jones Bay	36.377 S 174.824 E	-	Warty	Multiporates
Hapalidiales sp. ZZ	Hapalidiales	NZC2053	FJ361427	Auckland, Jones Bay	36.377 S 174.824 E	-	Encrusting	Uniporates
Hapalidiales sp. ZZ	Hapalidiales	NZC2162	KM369073	Auckland, Kiritehere Beach	38.325 S 174.703 E	-	Lumpy	Multiporates-Volcano
Hapalidiales sp. ZZ	Hapalidiales	NZC2164	FJ361475	Auckland, Kiritehere Beach	38.325 S 174.703 E	-	Lumpy	Multiporates-Volcano
Hapalidiales sp. ZZ	Hapalidiales	NZC2254	FJ361486	Auckland, Rangiatea	35.217 S 174.181 E	13m	Encrusting	Multiporates
Hapalidiales sp. ZZ	Hapalidiales	NZC2255	FJ361487	Auckland, Rangiatea	35.217 S 174.181 E	13m	Encrusting	Multiporates

Hapalidiales sp. ZZ	Hapalidiales	NZC2272	FJ361518	Auckland, Okahu Is.	35.201 S 174.205 E	14m	Warty	Multiporates
Hapalidiales sp. ZZ	Hapalidiales	NZC2374	FJ361624	Auckland, North Cape	34.418 S 173.052 E	8m	Encrusting	Multiporates
Hapalidiales sp. ZZ	Hapalidiales	NZC2478	FJ361630	Auckland, Wilson Bay	36.887 S 175.426 E	-	Encrusting	Multiporates
Hapalidiales sp. ZZ	Hapalidiales	NZC5083	MK413549	Southland, Foveaux strait	46.707 S 168.058 E	0	Warty	Multiporates - Volcano
Hapalidiales sp. ZZ	Hapalidiales	NZC5204	MK413512	Fiordland, Sunday Cove	45.594 S 166.741 E	1m	Encrusting	Multiporates - Volcano
Hapalidiales sp. ZZ	Hapalidiales	NZC5367	MK413410	Stewart Island, Horseshoe Bay	46.878 S 168.148 E	2m	Epilithic	Multiporates - Volcano
Hapalidiales sp. ZZ	Hapalidiales	NZC5500	MK413343	Otago, Green Island	45.952 S 170.386 E	0m	Encrusting	Multiporates - Volcano
Hapalidiales sp. ZZ	Hapalidiales	NZC5528	MK413331	Otago, Dunedin, Gull Rocks	45.905 S 170.651 E	6m	Encrusting	Multiporates - Volcano
Hapalidiales sp. ZZ	Hapalidiales	NZC5654A	MK413242	Fiordland, Narrow Bend	46.067 S 166.734 E	1m	Warty	Multiporates - Volcano
Lithothamnion crispatum	Hapalidiales	NZC2315	FJ361502	Auckland, Whau Point	35.233 S 174.245 E	-	Rhodolith	Multiporates- Volcano
Lithothamnion crispatum	Hapalidiales	NZC2411	FJ361589	Auckland, Cavalli Passage	35.02 S 173.948 E	13m	Rhodolith	Uniporates
Synarthrophyton patena	Hapalidiales	NZC5074	MK413554	Southland, Snout	46.26 S 167.194 E	0m	Discoid- Epiphytic	-
Synarthrophyton patena	Hapalidiales	NZC5113	MK413542	Southland, Foveaux strait	46.68 S 167.999 E	38m	Discoid- Epiphytic	-
Synarthrophyton patena	Hapalidiales	NZC5537A	MK413327	North Island, Glenburn Station	41.329 S 175.843 E	0m	Discoid	Uniporates- Pointy
Synarthrophyton patena	Hapalidiales	NZC5537B	MK413326	North Island, Glenburn Station	41.329 S 175.843 E	0m	Discoid	Multiporates - Flat top
Synarthrophyton patena	Hapalidiales	NZC5635	MK413246	Fiordland, Spit Islands	46.074 S 166.633 E	7m	Discoid- Epiphytic	Multiporates - Flat top
Corallinapetra novaezelandiae	incertae sedis	NZC2381	FJ361637	Auckland, Stephenson Is.	34.97 S 173.79 E	17m	Encrusting	Uniporates
Heydrichia homalopasta	Sporolithales	NZC0748	EF628210	Chatham Island, Port Hutt	43.816 S 176.705 W	-	Encrusting	Uniporates
Heydrichia homalopasta	Sporolithales	NZC0753	DQ167934	Chatham Island, Port Hutt	43.816 S 176.705 W	-	Encrusting	Uniporates
Heydrichia homalopasta	Sporolithales	NZC0757	DQ167937	Chatham Island, Port Hutt	43.816 S 176.705 W	-	Encrusting	Uniporates
Heydrichia homalopasta	Sporolithales	NZC2015	FJ361383	Auckland, Te Henga	36.887 S 174.437 E	-	Encrusting	Uniporates
Heydrichia homalopasta	Sporolithales	NZC2029	FJ361393	Auckland, Rocky Bay	36.819 S 175.053 E	-	Encrusting	Uniporates- Jelly
Heydrichia homalopasta	Sporolithales	NZC2052	FJ361548	Auckland, Jones Bay	36.377 S 174.824 E	-	Encrusting	Uniporates
Heydrichia homalopasta	Sporolithales	NZC2078	FJ361430	Auckland, Tutukaka	35.612 S 174.532 E	-	Encrusting	Uniporates
Heydrichia homalopasta	Sporolithales	NZC2092	FJ361403	Auckland, Ocean Beach	35.835 S 174.573 E	-	Encrusting	Multiporates
Heydrichia homalopasta	Sporolithales	NZC2111	FJ361438	Auckland, Matai Bay	34.828 S 173.411 E	-	Encrusting	Uniporates
Heydrichia homalopasta	Sporolithales	NZC2141	MK413616	Auckland, Cable Bay	34.99 S 173.487 E	-	Warty	Uniporates
Heydrichia homalopasta	Sporolithales	NZC2142	FJ361444	Auckland, Cable Bay	34.99 S 173.487 E	-	Encrusting	Uniporates
Heydrichia homalopasta	Sporolithales	NZC2184	FJ361570	Auckland, Tapeka Beach	35.243 S 174.118 E	-		-
Heydrichia homalopasta	Sporolithales	NZC2190	FJ361512	Auckland, Tapeka Beach	35.243 S 174.118 E	-		-

Heydrichia homalopasta	Sporolithales	NZC2194	FJ361539	Auckland, Bland Bay	35.346 S 174.367 E	-		-
Heydrichia homalopasta	Sporolithales	NZC2202	FJ361520	Auckland, Bland Bay	35.346 S 174.367 E	-	Encrusting	Uniporates
Heydrichia homalopasta	Sporolithales	NZC2206	FJ361503	Auckland, Bland Bay	35.346 S 174.367 E	-		Uniporates
Heydrichia homalopasta	Sporolithales	NZC2209	FJ361507	Auckland, Bland Bay	35.346 S 174.367 E	-	Encrusting	Uniporates
Heydrichia homalopasta	Sporolithales	NZC2264	FJ361559	Auckland, Moturoa & Motu channel	35.213 S 174.197 E	14m	Encrusting	Uniporates
Heydrichia homalopasta	Sporolithales	NZC2307	FJ361536	Auckland, Urupukapuka	35.205 S 174.234 E	14m	Encrusting	Uniporates
Heydrichia homalopasta	Sporolithales	NZC2436	FJ361652	Auckland, Little Bay	36.601 S 175.549 E	-	Encrusting	Calcified Compartments
Heydrichia homalopasta	Sporolithales	NZC2499	FJ361754	Auckland, Ahipara	35.175 S 173.117 E	-	Encrusting	Calcified Compartments
Heydrichia homalopasta	Sporolithales	NZC2516	FJ361748	Auckland, Henderson Point	34.741 S 173.118 E	-	Encrusting	Calcified Compartments
Heydrichia homalopasta	Sporolithales	NZC2549	FJ361707	Auckland, Kapowairua	34.421 S 172.856 E	-	Encrusting	Calcified Compartments
Heydrichia homalopasta	Sporolithales	NZC2595	MK413597	Gisborne, Lottin Point	37.553 S 178.164 E	-	Geniculate	-
Sporolithales sp. A	Sporolithales	NZC2014	FJ361360	Auckland, Te Henga	36.887 S 174.437 E	-	Encrusting	Uniporates
Sporolithales sp. A	Sporolithales	NZC2438	FJ361655	Auckland, Little Bay	36.601 S 175.549 E	-	Encrusting	Calcified Compartments
Sporolithales sp. A	Sporolithales	NZC2521	FJ361698	Auckland, Henderson Point	34.741 S 173.118 E	-	Encrusting	Calcified Compartments
Sporolithales sp. A	Sporolithales	NZC2541	FJ361702	Auckland, Kapowairua	34.421 S 172.856 E	-	Encrusting	Calcified Compartments
Sporolithon sp. A	Sporolithales	NZC0228	DQ167955	Nelson, Ataata Point	41.157 S 173.408 E	-	Encrusting	Calcified Compartments
Sporolithon sp. A	Sporolithales	NZC0249	EF628211	Nelson, Cable Bay	41.156 S 173.403 E	7m	Lumpy	Calcified Compartments
Sporolithon sp. A	Sporolithales	NZC0310	DQ167887	Nelson, Cable Bay	41.156 S 173.403 E	11m	Encrusting	Calcified Compartments
Sporolithon sp. A	Sporolithales	NZC0480	DQ167897	Nelson, Mussel Farm Bay	40.807 S 172.929 E	-	Encrusting	-
Sporolithon sp. A	Sporolithales	NZC2168	FJ361469	Auckland, Kiritehere Beach	38.325 S 174.703 E	-		Uniporates
Sporolithon sp. A	Sporolithales	NZC2173	FJ361478	Auckland, Whale Bay	37.822 S 174.802 E	-	Warty	Uniporates
Sporolithon sp. A	Sporolithales	NZC2175	KM369012	Auckland, Whale Bay	37.822 S 174.802 E	-	Lumpy	Calcified Compartments
Sporolithon sp. A	Sporolithales	NZC2177	FJ361476	Auckland, Whale Bay	37.822 S 174.802 E	-	Epilithic	Calcified Compartments
Sporolithon sp. A	Sporolithales	NZC2249	FJ361530	Auckland, Rangiatea	35.217 S 174.181 E	13m	Fruticose	Calcified Compartments
Sporolithon sp. A	Sporolithales	NZC2257	FJ361529	Auckland, Rangiatea	35.217 S 174.181 E	13m	Warty	Calcified Compartments
Sporolithon sp. A	Sporolithales	NZC2282	FJ361495	Auckland, Okahu Channel	35.197 S 174.216 E	14m	Encrusting	Calcified Compartments

Sporolithon sp. A	Sporolithales	NZC2284	FJ361540	Auckland, Okahu Channel	35.197 S 174.216 E	14m		Calcified Compartments
Sporolithon sp. A	Sporolithales	NZC2420	FJ361661	Auckland, Sailors Grave	36.961 S 175.844 E	-	Warty	Calcified Compartments
Sporolithon sp. A	Sporolithales	NZC2509	FJ361744	Auckland, Henderson Point	34.741 S 173.118 E	-	Encrusting	Calcified Compartments
Sporolithon sp. A	Sporolithales	NZC5596	MK413272	Otago, Moeraki	45.362 S 170.863 E	1m	Epilithic	-
Sporolithon sp. B	Sporolithales	NZC0137	EF628212	Nelson, Catherine Cove	40.863 S 173.891 E	20m	Rhodolith	-
Sporolithon sp. B	Sporolithales	NZC0497	DQ167962	Auckland, Ritch Bitch Hole	36.637 S 174.763 E	3m	Rhodolith	-
Sporolithon sp. B	Sporolithales	NZC0787	DQ167960	Hawkes Bay, Tuingara Point	40.121 S 176.875 E	15m	Encrusting	Multiporates
Sporolithon sp. B	Sporolithales	NZC0804	DQ167966	Wellington, Kapiti Is.	40.87 S 174.921 E	16m	Rhodolith	Uniporates
Sporolithon sp. B	Sporolithales	NZC0805	DQ167961	Wellington, Kapiti Is.	40.87 S 174.921 E	16m	Rhodolith	Uniporates
Sporolithon sp. B	Sporolithales	NZC0824	DQ167975	Wellington, Kapiti Is.	40.87 S 174.921 E	7m	Rhodolith	-
Sporolithon sp. B	Sporolithales	NZC0830	DQ167971	Wellington, Kapiti Is.	40.87 S 174.921 E	16m	Rhodolith	Uniporates
Sporolithon sp. B	Sporolithales	NZC0833	DQ167989	Wellington, Kapiti Is.	40.87 S 174.921 E	16m	Rhodolith	Uniporates
Sporolithon sp. B	Sporolithales	NZC0836	DQ167999	Wellington, Kapiti Is.	40.87 S 174.921 E	7m	Rhodolith	-
Sporolithon sp. B	Sporolithales	NZC0837	DQ167995	Wellington, Kapiti Is.	40.87 S 174.921 E	16m	Rhodolith	Uniporates
Sporolithon sp. B	Sporolithales	NZC0838	DQ167987	Wellington, Kapiti Is.	40.87 S 174.921 E	7m	Rhodolith	Uniporates
Sporolithon sp. B	Sporolithales	NZC0839	DQ167992	Wellington, Kapiti Is.	40.87 S 174.921 E	16m	Rhodolith	Uniporates
Sporolithon sp. B	Sporolithales	NZC0840	DQ167991	Wellington, Kapiti Is.	40.87 S 174.921 E	7m	Rhodolith	Uniporates
Sporolithon sp. B	Sporolithales	NZC0851	DQ167998	Wellington, Kapiti Is.	40.87 S 174.921 E	16m	Rhodolith	Uniporates
Sporolithon sp. B	Sporolithales	NZC2042	FJ361400	Auckland, Jones Bay	36.377 S 174.824 E	-	Rhodolith	Calcified Compartments
Sporolithon sp. B	Sporolithales	NZC2268	FJ361515	Auckland, Okahu Is.	35.201 S 174.205 E	14m	Rhodolith	Calcified Compartments
Sporolithon sp. B	Sporolithales	NZC2313	FJ361666	Auckland, Whau Point	35.233 S 174.245 E	-	Rhodolith	Calcified Compartments
Sporolithon sp. B	Sporolithales	NZC2314	FJ361527	Auckland, Whau Point	35.233 S 174.245 E	-	Rhodolith	Uniporates
Sporolithon sp. B	Sporolithales	NZC2323	FJ361573	Auckland, Wekarua Is.	34.936 S 173.654 E	10m	Rhodolith	Calcified Compartments
Sporolithon sp. B	Sporolithales	NZC2331	FJ361575	Auckland, Wekarua Is.	34.936 S 173.654 E	10m	Rhodolith	Calcified Compartments
Sporolithon sp. B	Sporolithales	NZC2375	KM369066	Auckland, Karikari Bay	34.827 S 173.378 E	11m	Rhodolith	Calcified Compartments
Sporolithon sp. B	Sporolithales	NZC2379	FJ361584	Auckland, Stephenson Is.	34.97 S 173.79 E	17m	Rhodolith	Calcified Compartments
Sporolithon sp. B	Sporolithales	NZC2385-1	FJ361639	Auckland, Cavalli Passage	35.02 S 173.948 E	13m	Rhodolith	Calcified Compartments
Sporolithon sp. B	Sporolithales	NZC2385-2	FJ361640	Auckland, Cavalli Passage	35.02 S 173.948 E	13m	Rhodolith	Calcified Compartments

Sporolithon sp. B	Sporolithales	NZC2388	FJ361586	Auckland, Motuharakeke	35.003 S 173.97 E	34m	Encrusting	Uniporates
Sporolithon sp. B	Sporolithales	NZC2396	FJ361599	Auckland, Haraweka Is.	34.98 S 173.952 E	9m	Rhodolith	Calcified Compartments
