# Supplementary material for: High diversity of coralline algae in New Zealand revealed: Knowledge gaps and implications for future research
Source: PLoS One. 2019 Dec 2;14(12):e0225645. doi: 10.1371/journal.pone.0225645 (PMC6886753; doi:10.1371/journal.pone.0225645)
Supplement: S4 Table — Hypothetical genera were based on well supported clades containing closely species within the New Zealand dataset. Specimens are listed alphabetically within the three orders.— = no data available. (PDF) [file pone.0225645.s004.pdf]

S4 Table. List of representative New Zealand coralline algal species, identified using species delimitation methods, used in concatenated *psb*A and *rbc*L phylogenetic analyses. Hypothetical genera were based on well supported clades containing closely species within the New Zealand dataset. Specimens are listed alphabetically within the three orders. - = no data available

				GenBank accession number	
**Name on tree**	**Order**	**Genus**	**Algae number**	***psb*A**	***rbc*L**
Amphiroa anceps NZC2361	Corallinales	*Amphiroa*	NZC2361	FJ361601	KM369162
Arthrocardia sp A NZC2157	Corallinales	*Arthrocardia*	NZC2157	FJ361466	MK674172
Arthrocardia sp B NZC2598	Corallinales	*Arthrocardia*	NZC2598	KM369087	KM369137
Arthrocardia sp C ASE107	Corallinales	*Arthrocardia*	ASE107	KM369030	KM369139
Corallina sp ASD027	Corallinales	*Corallina*	ASD027	DQ168011	KM369146
Corallinales sp A NZC5546	Corallinales	Genus 1	NZC5546	MK413318	-
Corallinales sp B NZC5472	Corallinales	Genus 2	NZC5472	MK413358	MK674185
Corallinales sp C NZC2547	Corallinales	Genus 3	NZC2547	KM369040	KM369154
Corallinales sp D NZC5138	Corallinales	Genus 4	NZC5138	MK413535	-
Corallinales sp E NZC5484	Corallinales	Genus 4	NZC5484	MK413353	MK674186
Corallinales sp F NZC2025	Corallinales	Genus 5	NZC2025	FJ361391	-
Corallinales sp G NZC2009	Corallinales	Genus 5	NZC2009	KM369046	KM369159
Corallinales sp H NZC5378	Corallinales	Genus 6	NZC5378	MK413405	MK674184
Corallinales sp I NZC5243	Corallinales	Genus 6	NZC5243	MK413490	MK674182
Corallinales sp J NZC5217	Corallinales	Genus 6	NZC5217	MK413507	MK674181
Corallinales sp K NZC2412	Corallinales	Genus 7	NZC2412	FJ361739	-
Corallinales sp L NZC2125	Corallinales	Genus 8	NZC2125	KM369107	KM369160
Corallinales sp M NZC5333	Corallinales	Genus 8	NZC5333	MK413431	MK674183
Corallinales sp N NZC0777	Corallinales	Genus 8	NZC0777	KM369047	KM369157
Corallinales sp O NZC2122	Corallinales	Genus 8	NZC2122	KM369042	KM369161
Corallinales sp P NZC0090	Corallinales	Genus 8	NZC0090	EF628237	KM369156
Corallinales sp Q NZC0667	Corallinales	Genus 9	NZC0667	EF628239	KM369153
Corallinales sp R NZC2487	Corallinales	Genus 10	NZC2487	KM369051	MK674179
Corallinales sp S ASN200	Corallinales	Genus 11	ASN200	MK413633	MK674173

Corallinales sp T NZC2545	Corallinales	Genus 12	NZC2545	KM369052	KM369165
Corallinales sp U NZC2055	Corallinales	Genus 13	NZC2055	KM369057	KM369166
Corallinales sp V NZC2250	Corallinales	Genus 14	NZC2250	KM369056	MK674176
Corallinales sp W NZC5673	Corallinales	Genus 15	NZC5673	MK413229	MK674187
Corallinales sp X NZC0314	Corallinales	Genus 15	NZC0314	EF628240	MK674174
Corallinales sp Y NZC2505	Corallinales	Genus 15	NZC2505	KM369053	KM369168
Corallinales sp Z NZC5418	Corallinales	Genus 15	NZC5418	MK413384	-
Corallinales sp ZA NZC2115	Corallinales	Genus 15	NZC2115	KM369054	KM369164
Corallinales sp ZB NZC2270	Corallinales	Genus 16	NZC2270	FJ361531	-
Corallinales sp ZC NZC2409	Corallinales	Genus 16	NZC2409	FJ361581	-
Corallinales sp ZD NZC2130	Corallinales	Genus 16	NZC2130	KM369055	KM369167
Corallinales sp ZE NZC2590	Corallinales	Genus 16	NZC2590	FJ361720	MK674180
Corallinales sp ZF NZC5562	Corallinales	Genus 16	NZC5562	MK413304	-
Corallinales sp ZG NZC5022	Corallinales	Genus 16	NZC5022	MK413580	-
Jania sagittata NZC2389	Corallinales	*Jania*	NZC2389	KM369032	MK674229
Jania sp A NZC5426	Corallinales	*Jania*	NZC5426	MK413379	MK674232
Jania sp B NZC2554	Corallinales	*Jania*	NZC2554	KM369034	KM369142
Jania sp C NZC2234	Corallinales	*Jania*	NZC2234	FJ361368	-
Jania sp E NZC5062	Corallinales	*Jania*	NZC5062	MK413560	MK674230
*Jani*a sp F NZC2022	Corallinales	*Jania*	NZC2022	KM369033	KM369143
Jania sp J ASD196	Corallinales	*Jania*	ASD196	EF628225	KM369140
Jania sphaeroramosa NZC5234	Corallinales	*Jania*	NZC5234	MH010587	MK674231
Mastophora pacifica NZC2000	Corallinales	*Mastophora*	NZC2000	FJ361365	KM369152
Neogoniolithon sp NZ2043	Corallinales	*Neogoniolithon*	NZC2043	FJ361401	KM369151
Pneophyllum sp A NZC2023	Corallinales	*Pneophyllum*	NZC2023	FJ361545	-
Pneophyllum sp B NZC5564	Corallinales	*Pneophyllum*	NZC5564	MK413302	-
Pneophyllum sp C ASN195	Corallinales	*Pneophyllum*	ASN195	MK413634	MK674236
Pneophyllum sp D NZC0507	Corallinales	*Pneophyllum*	NZC0507	EF628233	MK674237
*Pneophyllu*m sp E NZC5323	Corallinales	*Pneophyllum*	NZC5323	MK413439	MK674239
Pneophyllum sp F NZC0627	Corallinales	*Pneophyllum*	NZC0627	EF628234	MK674238

Pneophyllum sp G NZC0730	Corallinales	*Pneophyllum*	NZC0730	KM369048	-
Pneophyllum sp H NZC5746C	Corallinales	*Pneophyllum*	NZC5746C	MK413210	-
Pneophyllum sp I NZC5063	Corallinales	*Pneophyllum*	NZC5063	MK413559	-
Pneophyllum sp J NZC0686	Corallinales	*Pneophyllum*	NZC0686	KM369045	KM369158
Pneophyllum sp K NZC2019	Corallinales	*Pneophyllum*	NZC2019	KM369043	KM369155
Hapalidiales sp A NZC2090	Hapalidiales	Genus 1	NZC2090	KM369026	KM369136
Hapalidiales sp B NZC5574	Hapalidiales	Genus 1	NZC5574	MK413293	MK674225
Hapalidiales sp C NZC5470	Hapalidiales	Genus 1	NZC5470	MK413359	MK674222
Hapalidiales sp D NZC5447	Hapalidiales	Genus 1	NZC5447	MK413365	MK674219
Hapalidiales sp E NZC5224	Hapalidiales	Genus 1	NZC5224	MK413501	MK674205
Hapalidiales sp F NZC2238	Hapalidiales	Genus 2	NZC2238	FJ361372	-
Hapalidiales sp G NZC5623	Hapalidiales	Genus 2	NZC5623	MK413253	MK674226
Hapalidiales sp H NZC5294	Hapalidiales	Genus 3	NZC5294	MK413456	MK674207
Hapalidiales sp I NZC2013	Hapalidiales	Genus 3	NZC2013	KM369024	KM369134
Hapalidiales sp J NZC2041	Hapalidiales	Genus 4	NZC2041	MK413625	-
Hapalidiales sp K NZC5440	Hapalidiales	Genus 5	NZC5440	MK413369	MK674218
Hapalidiales sp L NZC0847	Hapalidiales	Genus 6	NZC0847	DQ167993	MK674190
Hapalidiales sp M NZC2439-1	Hapalidiales	Genus 6	NZC2439-1	FJ361629	MK674196
Hapalidiales sp N NZC5345	Hapalidiales	Genus 6	NZC5345	MK413420	MK674210
Hapalidiales sp O NZC2236	Hapalidiales	Genus 6	NZC2236	KM369018	KM369126
Hapalidiales sp P NZC5362A	Hapalidiales	Genus 7	NZC5362A	MK413413	MK674213
Hapalidiales sp Q NZC5379	Hapalidiales	Genus 7	NZC5379	MK413404	MK674215
Hapalidiales sp R NZC5306	Hapalidiales	Genus 7	NZC5306	MK413451	MK674208
Hapalidiales sp S CUK17874	Hapalidiales	Genus 7	CUK17874	MK702009	MK702013
Hapalidiales sp T NZC5406	Hapalidiales	Genus 7	NZC5406	MK413391	-
Hapalidiales sp U NZC5290B	Hapalidiales	Genus 7	NZC5290B	MK413458	-
Hapalidiales sp V NZC2288	Hapalidiales	Genus 7	NZC2288	KM369078	KM369127
Hapalidiales sp W NZC0875	Hapalidiales	Genus 8	NZC0875	DQ167990	MK674191
Hapalidiales sp X NZC4004	Hapalidiales	Genus 9	NZC4004	MK413592	MK674197
Hapalidiales sp Y NZC5202	Hapalidiales	Genus 10	NZC5202	MK413514	-

Hapalidiales sp YA NZC0747	Hapalidiales	Genus 29	NZC0747	KM369025	KM369135
Hapalidiales sp YB NZC0709	Hapalidiales	Genus 30	NZC0709	KM369014	KM369128
Hapalidiales sp YC NZC5028	Hapalidiales	Genus 30	NZC5028	MK413577	MK674198
Hapalidiales sp YD NZC5292	Hapalidiales	Genus 30	NZC5292	MK413457	MK674206
Hapalidiales sp YE NZC2365	Hapalidiales	Genus 30	NZC2365	KM369021	MK674193
Hapalidiales sp YF NZC2342	Hapalidiales	Genus 30	NZC2342	KM369020	KM369131
Hapalidiales sp YG NZC5241	Hapalidiales	Genus 31	NZC5241	MK413491	MK674241
Hapalidiales sp Z NZC0899	Hapalidiales	Genus 11	NZC0899	KM369015	KM369132
Hapalidiales sp ZA NZC5368	Hapalidiales	Genus 12	NZC5368	MK413409	MK674214
Hapalidiales sp ZB NZC5697	Hapalidiales	Genus 12	NZC5697	MK413221	MK674227
Hapalidiales sp ZC NZC0476	Hapalidiales	Genus 12	NZC0476	KM369013	KM369125
Hapalidiales sp ZD NZC5080	Hapalidiales	Genus 13	NZC5080	MK413550	MK674200
Hapalidiales sp ZE NZC5469	Hapalidiales	Genus 14	NZC5469	MK413360	MK674221
Hapalidiales sp ZF NZC5361	Hapalidiales	Genus 15	NZC5361	MK413414	MK674212
Hapalidiales sp ZG NZC5425A	Hapalidiales	Genus 16	NZC5425A	MK413381	MK674216
Hapalidiales sp ZH NZC5501	Hapalidiales	Genus 17	NZC5501	MK413342	MK674223
Hapalidiales sp ZI NZC5079	Hapalidiales	Genus 18	NZC5079	MK413551	MK674199
Hapalidiales sp ZJ CUK18093	Hapalidiales	Genus 19	CUK18093	MK702010	MK702012
Hapalidiales sp ZK NZC5354	Hapalidiales	Genus 20	NZC5354	MK413416	MK674211
Hapalidiales sp ZL NZC5429	Hapalidiales	Genus 21	NZC5429	MK413377	MK674217
Hapalidiales sp ZM CUK18097	Hapalidiales	Genus 21	CUK18097	MK702011	MK702014
Hapalidiales sp ZN NZC5548	Hapalidiales	Genus 22	NZC5548	MK413316	MK674224
Hapalidiales sp ZO NZC2371	Hapalidiales	Genus 22	NZC2371	FJ361610	-
Hapalidiales sp ZP NZC5698A	Hapalidiales	Genus 22	NZC5698A	MK413220	-
Hapalidiales sp ZQ NZC5095	Hapalidiales	Genus 23	NZC5095	MK413544	MK674201
Hapalidiales sp ZR NZC5308B	Hapalidiales	Genus 24	NZC5308B	MK413450	MK674209
Hapalidiales sp ZS NZC0772	Hapalidiales	Genus 25	NZC0772	EF628215	MK674189
Hapalidiales sp ZT NZC2501	Hapalidiales	Genus 25	NZC2501	KM369023	KM369133
Hapalidiales sp ZU NZC5140	Hapalidiales	Genus 26	NZC5140	MK413534	MK674202
Hapalidiales sp ZV NZC5221	Hapalidiales	Genus 27	NZC5221	MK413504	MK674203

Hapalidiales sp ZW NZC2317	Hapalidiales	Genus 27	NZC2317	MK413609	MK674192
Hapalidiales sp ZX NZC5223	Hapalidiales	Genus 27	NZC5223	MK413502	MK674204
Hapalidiales sp ZY NZC2433	Hapalidiales	Genus 28	NZC2433	FJ361750	MK674195
Hapalidiales sp ZZ NZC2051	Hapalidiales	Genus 28	NZC2051	KM369019	KM369129
Lithothamnion crispatum NZC2411	Hapalidiales	*Lithothamnion*	NZC2411	FJ361589	MK674234
Synarthrophyton patena NZC5537A	Hapalidiales	*Synarthrophyton*	NZC5537A	MK413327	MK674242
Heydrichia homalopasta NZC2015	Sporolithales	*Heydrichia*	NZC2015	FJ361383	MK674228
Sporolithales sp A NZC2014	Sporolithales	Genus 1	NZC2014	FJ361360	KM369120
Sporolithon sp A NZC2175	Sporolithales	*Sporolithon*	NZC2175	KM369012	KM369123
Sporolithon sp B NZC2375	Sporolithales	*Sporolithon*	NZC2375	KM369066	KM369122
