# Supplementary material for: High diversity of coralline algae in New Zealand revealed: Knowledge gaps and implications for future research
Source: PLoS One. 2019 Dec 2;14(12):e0225645. doi: 10.1371/journal.pone.0225645 (PMC6886753; doi:10.1371/journal.pone.0225645)
Supplement: S5 Table — (PDF) [file pone.0225645.s005.pdf]

S5 Table. The average percentage cover and relative abundance ± 1 Standard Error (SE) of coralline algal species across a series of six boulders in Moeraki, Otago, New Zealand.

Species	Average cover (%) ± 1 SE	Relative abundance (%) ± 1 SE
Corallinales sp. A	1.8 ± 1.2	2.3 ± 1.6
Corallinales sp. ZF	12.3 ± 3.6	14.4 ± 3.8
Corallinales sp. ZG	19.2 ± 6.2	22.5 ± 7.3
Hapalidiales sp. B	3 ± 2.5	3.3 ± 2.6
Hapalidiales sp. D	4.2 ± 2.1	5.1 ± 2.7
Hapalidiales sp. E	3 ± 2.5	3.7 ± 3
Hapalidiales sp. ZN	0.8 ± 0.8	0.8 ± 0.9
Hapalidiales sp. ZT	4.6 ± 3.1	6.2 ± 4.3
Hapalidiales sp. ZX	0.7 ± 0.7	1 ± 1
Hapalidiales sp. ZY	0.5 ± 0.5	0.5 ± 0.5
Hapalidiales sp. YA	21.5 ± 9.6	24.2 ± 10.2
Hapalidiales sp. H	3.2 ± 2.5	3.3 ± 2.6
Hapalidiales sp. I	4.5 ± 2	5.4 ± 2.4
Hapalidiales sp. YD	3.7 ± 3.7	5.1 ± 5.1
Hapalidiales sp. U	1 ± 1	1 ± 1
Pneophyllum sp. B	0.5 ± 0.5	0.6 ± 0.6
Sporolithon sp. A	0.7 ± 0.7	0.7 ± 0.7
