# Supplementary material for: High diversity of coralline algae in New Zealand revealed: Knowledge gaps and implications for future research
Source: PLoS One. 2019 Dec 2;14(12):e0225645. doi: 10.1371/journal.pone.0225645 (PMC6886753; doi:10.1371/journal.pone.0225645)
Supplement: Attachment — Submitted filename: Response to Reviewers.docx [file pone.0225645.s006.docx]

**RESPONSE LETTER**

Manuscript ID number: PONE-D-19-23390

Manuscript title: High diversity of coralline algae in New Zealand revealed: knowledge gaps and implications for research on the roles of these foundation species

Author(s): Twist B, Neill K, Bilewitch J, Jeong SY, Sutherland J & Nelson W

Dear Tzen-Yuh Chiang,

We are submitting a revised version of manuscript PONE-D-19-23390, in which we have made changes in response to the comments we received.

Those comments are pasted in the table below with our response. All changes made to the manuscript are shown through the track change feature in MS word and page and line numbers relating to specific comments indicated on the table below. A ‘clean’ version of this manuscript has also been submitted with all track changes accepted.

We would like to thank the reviewers for their constructive feedback and their time in reviewing the manuscript.

Sincerely,

Brenton and Co-authors

| Comment no. | Reviewer comment | Line no. in original ms | Line no. in revised track change ms | Response |
| --- | --- | --- | --- | --- |
|  | Journal Requirements: |  |  |  |
| 1 | Please ensure that your manuscript meets PLOS ONE's style requirements, including those for file naming. The PLOS ONE style templates can be found at  http://www.journals.plos.org/plosone/s/file?id=wjVg/PLOSOne_formatting_sample_main_body.pdf and http://www.journals.plos.org/plosone/s/file?id=ba62/PLOSOne_formatting_sample_title_authors_affiliations.pdf |  |  | The only issue found with style requirements was:  Fig. has been changed to Fig  Name of manuscript has been changed to just “manuscript” |
| 2 | In your Methods section, please provide additional information regarding the permits you obtained for the work. Please ensure you have included the full name of the authority that approved the field site access and, if no permits were required, a brief statement explaining why. |  | 114 | The following statement has been added to the start of the of the Methods  “All collections were made under Special Permit 665 issued by the New Zealand Ministry for Primary Industries (MPI) which allows the taking of seaweed for the purposes of education and investigative research.” |
| 3 | We note that Figure(s) 1 in your submission contain [map/satellite] images which may be copyrighted. All PLOS content is published under the Creative Commons Attribution License (CC BY 4.0), which means that the manuscript, images, and Supporting Information files will be freely available online, and any third party is permitted to access, download, copy, distribute, and use these materials in any way, even commercially, with proper attribution. For these reasons, we cannot publish previously copyrighted maps or satellite images created using proprietary data, such as Google software (Google Maps, Street View, and Earth). For more information, see our copyright guidelines: http://journals.plos.org/plosone/s/licenses-and-copyright. | Figure 1 | Figure 1 pg149 | Figure one was created by B Twist using freely available software QGIS. The Coast NZTM2000 vector layer was used in the creation <https://www.linz.govt.nz/data/geodetic-system/datums-projections-and-heights/projections/new-zealand-transverse-mercator-2000> which is “... licenced under the Creative Commons Attribution 4.0 International licence. In essence you are free to copy, distribute, and adapt the work, as long as you attribute the work to LINZ and abide by the other licence terms.”  Text was added to figure caption. “Map was created using QGIS software [44] with the use of New Zealand Transverse Mercator 2000 (NZTM2000) projection (LINZ; Land Information New Zealand).” |
|  | To enhance the reproducibility of your results, we recommend that if applicable you deposit your laboratory protocols in protocols.io, where a protocol can be assigned its own identifier (DOI) such that it can be cited independently in the future. For instructions see: http://journals.plos.org/plosone/s/submission-guidelines#loc-laboratory-protocols |  |  | The DNA extraction method has been published previously and so it is not appropriate for us to add it to protocols.io. Other methods are standard and it seems not worthwhile to add to this site. |
|  |  |  |  |  |
|  | Reviewer Comments |  |  |  |
|  | Reviewer 1 |  |  |  |
| 1 | Title: I would shorten it by cutting the words on the roles of these foundation species because their role is already assesses elsewhere and the paper does not contribute to the topic (systematics, not ecology) |  |  | The tile has been shortened. “Foundation species” has been added to the list of key words as we fell this is important definition of coralline algae relevant to ecological readers. |
| 2 | Corallines are not only stabilise coral reef structures but also are the major framework builders in the algal reefs of the temperate latitudes. This must clearly stated in the sentence, along with appropriate reference (i.e. Ballesteros 2006, Bracchi et al 2017 Continental Shelf Research 144:10-20; Bracchi et al. 2019) | 67 | 67 | This sentence has been changed to “They also are major builders and stabilisers of reef framework in temperate and tropical regions and play….”. The references Bracchi et al 2017 has been added. |
| 3 | in the recovery (swap of words) | 67 | 68 | Fixed |
| 4 | a list of dozens of citations is far too much. I suggest shorten it to a smaller group of references by selecting examples of the contribution of DIFFERENT groups of authors and diverse geographic areas wordwide. Please add reference to Kato works in Japan (for example Kato et al 2011 J of Phycology) and Basso work in the Red Sea/Indian Ocean (Basso et al 2015 Phytotaxa). | 78 | 79 | This number of citations has been reduced and examples of the contribution of different groups of authors and diverse geographic areas worldwide have been included in the references including work by Kato and Basso. A number of the citations listed here have been referred to elsewhere in the text and all of these citations have been retained. |
| 5 | Please add reference to the revision of L. kaiseri (Basso et al 2015 Phytotaxa) | 93 |  | This reference would not make sense here. The reference currently cited is backing up the statement that L kaiseri has wide distribution, which is shown in the Hernandez-Kantun et al 2016 paper. The Basso 2015 paper states the distribution of L kaiseri beyond the red sea is unknown. |
| 6 | Please declare which is the higher ranks taxonomic framework for your work. Hapalidiales, Sporolithales etc are used but no reference to high rank taxonomy is provided, and this also has been changing recently, therefore it is needed to specify. | Lines 173 and following | 179 | There are a number of places where the recent changes to the systematics of this group are referred to.  The sentence has been modified to:  “….two separate datasets of taxa belonging to the Corallinophycidae: (1) members of the order Corallinales, and (2) members of the orders Hapalidiales and Sporolithales. “ |
| 7 | These lines are intriguing but unclear. Has something to do with Corallinapetra (ref 16?)? Why do not declaring explicitly? I suggest expanding this in the discussion | 265-269 | 271-278 | This paragraph has been reworded to improve clarity. |
| 8 | Not apparent from morphological features. This is the most important issue in the manuscript. The paper does not discuss the morphological characters at all. 1) They are considered as a whole not useful a priori; 2) No coralline identification has never been possible in the field; 3) Badly conducted morphological analyses, such those relying on few badly oriented SEM pictures, will never provide any result. New morphological characters could be found if a serious morphological approach would be performed along with the genetics, which is the approach that I would recommend to follow and that I suggest to declare as recommended in the conclusions of this paper. | 396. |  | Throughout this manuscript there are many references to the issues about the use of morphology and anatomy for distinguishing these algae.  The purpose of this paper was to use genetic approaches to assess diversity and compare this to previous understandings.  We point to the need for further research to determine characters by which genera and species will be able to be distinguished. (e.g. lines 533-536) |
| 9 | The chapter title is too long | 431 | 445 | Changed to “Consequences of high diversity” |
| 10 | Understanding the nearshore community is not an issue tackled here, please remove. | 467 | 479 | This sentence is referring to the work by Hind et al 2019 paper and not our work. Sentence has been clarified to “This work by Hind et al. ….” |
| 11 | The reference 16 is used in a positive sense previously (see Lines 265-269), while here it is said that identification is incorrect. I understand that this probably referes to some further work in preparation, but you have to try to be a little bit clearer. | 472 | 485 | Reference 17 refers to the Corallinapetra paper by Nelson et al. 2015. These references are used here at the start of the section on “Consequences for taxonomy of New Zealand coralline algae”, to indicate that many of the names used for genera and species in NZ we now know were misapplied.  Reference to Broom et al. (48) has also been inserted here. |
| 12 | Please refer here explicitly to the need of further detailed morphological description based on groups emerging from molecular taxonomy. | 512 |  | This is referred to in lines 533-536. Stating the need for morpho-anatomical characterisation. |
|  |  |  |  |  |
|  | **Reviewer 2** |  |  |  |
| 1 | Remove the ':" in the title | 3 | 3 | The title has been changed. See reviewer 1 comments. |
| 2 | in the list of new orders the reference to the Sporolithales is missing. Le Gall, L., Payri, C.E., Bittner, C.E., & Saunders, G.W. (2010). Multigene polygenetic analyses support recognition of the Sporolithales, ord. nov. Molecular Phylogenetics and Evolution 54(1): 302-305. | 74 | 75 | This reference has now been added. Reference 18 |
| 3 | Although this is a regional study..." it sounds like the authors try to justify themself... I think that the number of species they uncovered is even more striking giving the regional scale! | 101 |  | We agree the number of species discovered is striking. In this statement we are noting the study has relevance beyond the study region. |
| 4 | The inclusion of type only sequence from GenBank is both a good and not so good idea in my mind. In a taxonomic point of view, I fully understand this strategy; however, in a evolutionary point of view, a phylogeny should include a balanced sampling of all the taxa in the studied group, no matter where they are from... I know that there are a wealth of coralline sequences in GenBank with some rather poorly annotated but I still think that it would provide valuable information to densify a bit the taxa sampling for the phylogenetic inferences of this study. An intermediate strategy may consist in including specimens for which both psbA and rbcL are available... In addition of improving the phylogenies this strategy would allow to assess the distribution of the taxa encountered in New Zealand (are they endemic or not?) | 207 |  | The purpose of these analyses was to anchor our phylogenies with reliably identified genera and species and thus our decision to only include sequences from type material available in GenBank.  As the reviewer points out it would be interesting and valuable to determine if there is any other material like ours from other regions, but this was not the purpose of the study.  We have taken a conservative view because we are very conscious of the impacts of misapplied names holding back progress in coralline systematics. We did not want to add to the confusion by including named sequences from GenBank for which we cannot check identification. |
| 5 | In my previous experience, with short markers as psbA and rbcL, I tend to find that the partition by codon only (without spliting the dataset by gene) is the best partition. Did this option was investigated with a program such as partition finder? | 213 | 218 | We used ModeFinder implemented in IQ-Tree to estimate optimal partitioning strategies and associated models. We have reworded the text to make this clearer (218-221). ModelFinder starts with a fully partitioned model and merges partitions until the model fit does not increase further, so the possibility of split by codon but not by gene would have been tested by this algorithm. |
| 6 | It may worth to mention whether the 16 species look like Lithophyllum or Amphiroa based on gross morphology.. | 297 | 305 | Great suggestion. This sentence has been modified to include the statement “...,despite a number of these being initially assigned as Lithophyllum spp. based on a select number of morpho-anatomical features.” |
| 7 | Same here, it may worth to mention if in the field they were identify as putative Mesophyllum... | 322 | 331 | This sentence has been modified to include. ”… , despite initially being assigned to this genus based on morpho-anatomical features, …” |
| 8 | I am not sure that it makes sense to have decimal for the estimation of species diversity | 347 |  | We agree that having species estimation as a decimal does not make sense. However, the way the species are estimated is a purely statistical method and returns results that are not whole integers, which we feel is important to retain in the results. In Table 1 and elsewhere in the text (e.g. 1^st^ paragraph of the discussion) these have been rounded down to the nearest integer (this is stated in the table text). |
| 9 | In this paragraph of discussion dedicated to "species diversity", it would be a good addition to refer to the global distribution of seaweeds such as Keith et al (2014) or Kerswell et al (2006)  Keith S. A., Kerswell A. P. & Connolly S. R. 2014 - Global diversity of marine macroalgae: environmental conditions explain less variation in the tropics: Global diversity of marine macroalgae. Global Ecology and Biogeography 23 (5) : 517–529.  Kerswell A. P. 2006 - Global biodiversity patterns of benthic marine algae. Ecology 87 (10) : 2479–2488. | 399 | 414 | A sentence has been added to this section in the discussion referring to how the global distribution of seaweeds tends to peak at mid-high latitudes. Bolton 1994, Keith et al. 2014 and Kerswell 2006 have been added to support this statement. |
| 10 | This paragraph is excellent! | 431 |  | Thank you! |
| 11 | fig. 2 & fig. 3 With so few annotation, it is very difficult to get oriented in these trees. Would it be possible to add the families or the delimited taxa with a number which refer to a table? | Figs 2 & 3 | Figs 2 & 3 | The purpose of these figures was to show the consistency of the species delimitation methods.  The reason for so few annotations is that there are so few names to use as pointed out in the text. We experimented with adding species letter codes that have been used to define species in table S3 but this resulted in a messy and illegible figure.  Our reluctance to include families was because the foundation for their recognition is contingent on good generic boundaries – and currently many of these are poorly defined. We would be most reluctant to attempt this.  A couple more labels have been added to fig 2 & 3 phylogenetic trees. These include the genera Lithothamnion, Heydrichia & Sporolithon in Fig 3 and Amphiroa in Fig 2. |
| 12 | fig. 4 Why no Rhodogorganales are included? This is the sister taxa to the Sporolithales and therefore the best outgroup! | Fig 4 |  | Rhodogorgonales would be an appropriate choice – but we choose to use representatives of each of our three orders as outgroups for one another. We could reanalyse but do not consider this will result in any significant differences.  We do include the genus Corallinapetra in all analyses – which is also closely related to Sporolithales (and is currently being proposed as the type of a new order of coralline algae). |
| 13 | fig. 5 Neogoniolithon has an extremely long branch (as usual!). It may worth to try to remove it or to break the branch by adding some other species... | Fig 5 |  | As the reviewer notes, Neogoniolithon is consistently resolved on long branches in analyses such as these. Earlier analyses performed with and without Neogoniolithon showed that its inclusion made little or no difference to the topology. We prefer to leave this taxon in for completeness. |
|  |  |  |  |  |
|  |  |  |  |  |
