# Supplementary material for: High diversity of coralline algae in New Zealand revealed: Knowledge gaps and implications for future research
Source: PLoS One. 2019 Dec 2;14(12):e0225645. doi: 10.1371/journal.pone.0225645 (PMC6886753; doi:10.1371/journal.pone.0225645)
Supplement: Attachment — Submitted filename: Response to Reviewers.docx [file pone.0225645.s007.docx]

**RESPONSE LETTER**

Manuscript ID number: PONE-D-19-23390

Manuscript title: High diversity of coralline algae in New Zealand revealed: knowledge gaps and implications for future research

Author(s): Twist B, Neill K, Bilewitch J, Jeong SY, Sutherland J & Nelson W

Dear Tzen-Yuh Chiang,

We are submitting a revised version of manuscript PONE-D-19-23390, in which we have made changes in response to the comments we received (pasted below).

All changes that were suggested by reviewer 1 have been made. These include adding authorities to orders and species of corallines mentioned in the manuscript and removing the sentence relating to Sporolithon in the fossil record (lines 509-510). These changes are shown through the track change feature in MS word. A ‘clean’ version of this manuscript has also been submitted with all track changes accepted.

We would like to thank the reviewers for their constructive feedback and their time in reviewing the manuscript.

Sincerely,

Brenton and Co-authors

______________________

Reviewer #1: Please add authors and adequate reference to previous taxonomic work throughout the manuscript. In particular:

Line 55 Add authorship of the three orders the first time you mention them

Line 94-95 Add authorship of these species the first time you mention in the manuscript: Lithophyllum kaiseri, Hydrolithon boergesenii, Sporolithon indopacificum

Line 97 add authorship for Phymatolithon the first time you mention in the manuscript

Line 213 Add authorship for Corallinapetra novaezelandiae the first time you mention in the manuscript

Line 271 Add authorship for Sporolithon, Heydrichia

Line 290 add authorship for Mastophora pacifica

Line 294 add authorship for Jania sagittata, Jania sphaeroramosa and to the other species mentioned on this same paragraph and in

Lines 509-510. The distribution of Sporolithon has been probably ruled by many other environmental controls in addition to temperature. It seems a deep genus in the tropics, but shallower in New Zealand as in the Red Sea, toward the limits of its distribution (Basso et al. 2009, Palaios), but it’s again deep in the Mediterranean. The maximum Late Cretaceous temperature is believed to be comparable to that of the PETM, but Sporolithon did not become abundant again during PETM. The picture is much more complicate than just temperature, and there is coralline evolution in between and increasing competition. I suggest to remove the comment about Sporolithon and the fossil record and the related reference.

Reviewer #2: I was very pleased to read the revised version of this manuscript. The authors addressed all my comments and I consider this new version ready to be published.
